# Supplementary figures and images for: Analyzing the performance of deep learning splice prediction algorithms
Source: PLoS One. 2026 May 13;21(5):e0348885. doi: 10.1371/journal.pone.0348885 (PMC13170886; doi:10.1371/journal.pone.0348885)

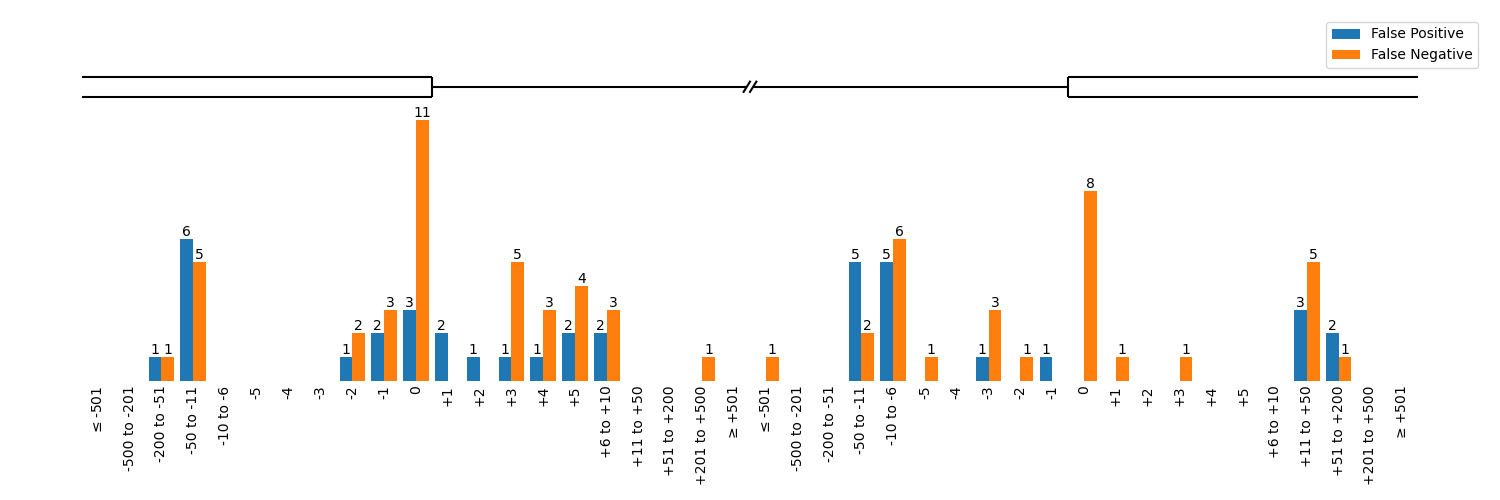

Supplement: S1 File — The data used in the CI-SpliceAI benchmark analysis are available in CI-SpliceAI__Comparison at https://github.com/YStrauch/CI-SpliceAI__Comparison. (ZIP) [file pone.0348885.s001.zip › CI-SpliceAI__Comparison-master/analysis/predictions/cis-fp-fn.png]

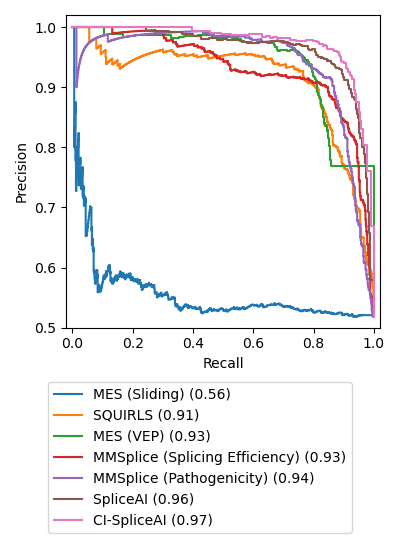

Supplement: S1 File — The data used in the CI-SpliceAI benchmark analysis are available in CI-SpliceAI__Comparison at https://github.com/YStrauch/CI-SpliceAI__Comparison. (ZIP) [file pone.0348885.s001.zip › CI-SpliceAI__Comparison-master/analysis/predictions/pr-auc.png]

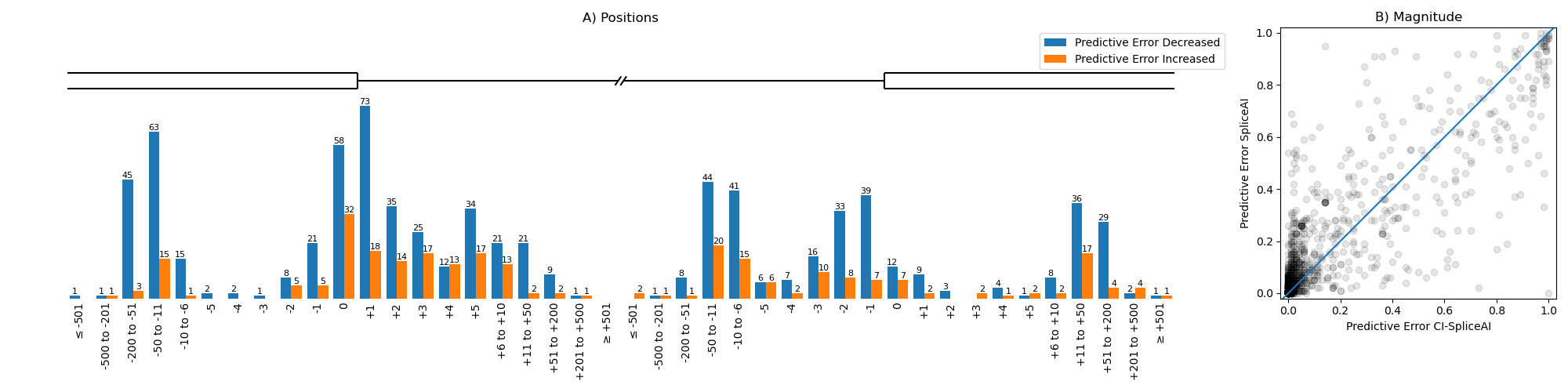

Supplement: S1 File — The data used in the CI-SpliceAI benchmark analysis are available in CI-SpliceAI__Comparison at https://github.com/YStrauch/CI-SpliceAI__Comparison. (ZIP) [file pone.0348885.s001.zip › CI-SpliceAI__Comparison-master/analysis/predictions/predictive-errors.png]

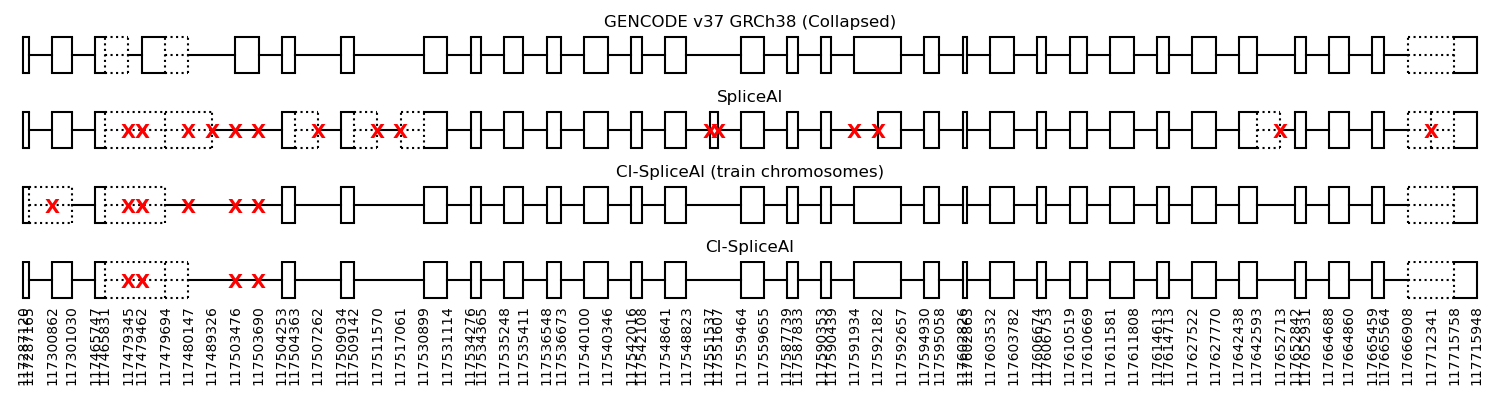

Supplement: S1 File — The data used in the CI-SpliceAI benchmark analysis are available in CI-SpliceAI__Comparison at https://github.com/YStrauch/CI-SpliceAI__Comparison. (ZIP) [file pone.0348885.s001.zip › CI-SpliceAI__Comparison-master/analysis/splicing/CFTR.png]

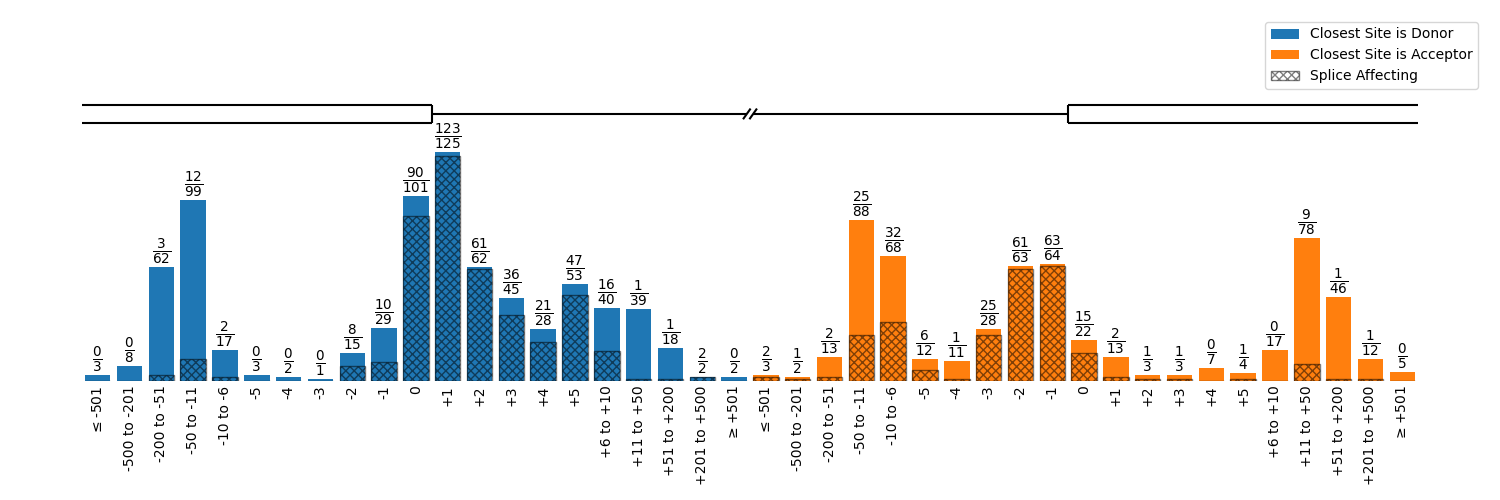

Supplement: S1 File — The data used in the CI-SpliceAI benchmark analysis are available in CI-SpliceAI__Comparison at https://github.com/YStrauch/CI-SpliceAI__Comparison. (ZIP) [file pone.0348885.s001.zip › CI-SpliceAI__Comparison-master/analysis/variants/distance-label.png]

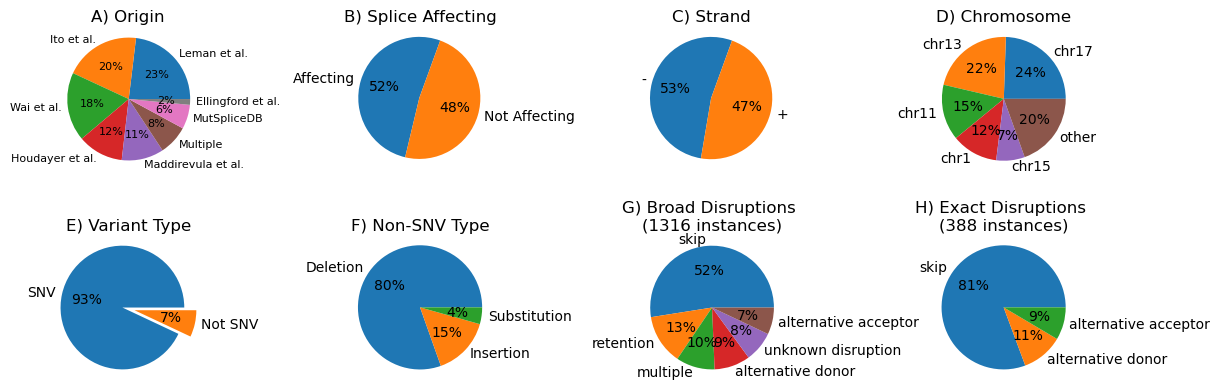

Supplement: S1 File — The data used in the CI-SpliceAI benchmark analysis are available in CI-SpliceAI__Comparison at https://github.com/YStrauch/CI-SpliceAI__Comparison. (ZIP) [file pone.0348885.s001.zip › CI-SpliceAI__Comparison-master/analysis/variants/pies.png]
