# Supplementary material for: Analyzing the performance of deep learning splice prediction algorithms
Source: PLoS One. 2026 May 13;21(5):e0348885. doi: 10.1371/journal.pone.0348885 (PMC13170886; doi:10.1371/journal.pone.0348885)
Supplement: S5 File — The Barbosa benchmark data are available at https://github.com/PedroBarbosa/DeepIntronic_Benchmark. (ZIP) [file pone.0348885.s005.zip › Barbosa_data/splicing_pathogenic_manual_curation/2_interpretability/squirls/SQUIRLS_report.html]

Squirls - Super-Quick Information Content and Random Forest Learning for Splice Variants


# *Squirls* analysis results for

*Squirls* is a desktop Java application that performs prediction
of deleteriousness of genomic variants with respect to mRNA splicing.
Variants are prioritized based on interpretable splicing features using
a machine learning algorithm. The functional annotation code is performed
using the Jannovar
library.

See the comprehensive
documentation learn how to setup and run *Squirls*, and how
to interpret the results.

## Analysis input

### Analyzed variants

*Squirls* analyzed variants stored in the VCF file located at:

SQUIRLS\_correct\_preds.vcf

### Transcript definitions

Squirls uses
Jannovar
library to perform functional annotation of variants
with respect to genes and transcripts.
The analysis used transcript definitions provided by *ENSEMBL*.

### Number of reported variants

After the analysis, the variants are sorted by predicted
pathogenicity and the variant with the highest predicted
pathogenicity is placed on the top of the list.
This report presents the top
**121**
most pathogenic variants.

## Statistics

|  |  |
| --- | --- |
| Number of variants in the VCF file | 121 |
| Number of distinct *ALT* alleles in the VCF file | 121 |
| Number of *ALT* alleles annotated by Squirls | 121 |
| Number of reported variants | 121 |

## Prioritised Variants

**6:6,225,105 C>T**

***F13A1***

Squirls score:
**1.000**

The variant overlaps with 1
transcript:

| Transcript Accession | CDS Change | Variant Effect | Squirls Score |
| --- | --- | --- | --- |
| *ENST00000264870.3* | c.799-12G>A | CODING\_TRANSCRIPT\_INTRON\_VARIANT | 1.000 |

Squirls features

| Feature | Value |
| --- | --- |
| *ΔRi* canonical acceptor | 0.14 |
| *ΔRi* cryptic acceptor | 8.05 |
| Creates AG in *AGEZ* | Yes |
| Acceptor offset | -12 |
| Exon length | 175 |
| ESRSeq | -1.89 |
| SMS | -1.52 |
| phyloP | 0.05 |

Cryptic acceptor

Using cryptic acceptor site at *6:6,225,107*
would lead to addition
of 10
bases
to the coding sequence.

The variant creates *AG* dinucleotide in the
AG exclusion zone
of the canonical acceptor site.

Canonical acceptor site

G
C
A
T
G
A
C
T
G
A
C
T
G
A
C
T
G
A
C
T
G
A
C
T
G
A
C
T
G
A
C
T
G
A
C
T
A
G
C
T
A
G
C
T
A
G
C
T
A
G
C
T
A
G
C
T
A
G
C
T
A
G
C
T
A
G
C
T
G
A
C
T
G
A
C
T
G
A
C
T
G
A
C
T
G
A
C
T
G
A
T
C
C
T
G
A
A
T
C
G
T
C
A
G
C
G
A
T
-25
.
.
.
.
-20
.
.
.
.
-15
.
.
.
.
.
-9
-8
-7
-6
-5
-4
-3
-2
-1
1
2
t
c
t
c
a
c
t
t
c
t
c
a
c
g
g
a
c
t
c
a
t
t
t
a
g
g
t
a

Predicted cryptic acceptor site

G
C
A
T
G
A
C
T
G
A
C
T
G
A
C
T
G
A
C
T
G
A
C
T
G
A
C
T
G
A
C
T
G
A
C
T
A
G
C
T
A
G
C
T
A
G
C
T
A
G
C
T
A
G
C
T
A
G
C
T
A
G
C
T
A
G
C
T
G
A
C
T
G
A
C
T
G
A
C
T
G
A
C
T
G
A
C
T
G
A
T
C
C
T
G
A
A
T
C
G
T
C
A
G
C
G
A
T
-35
.
.
.
.
.
-30
.
.
.
.
.
-25
.
.
.
.
.
-20
.
.
.
.
.
-15
.
.
.
.
.
-10
.
.
c
t
t
t
c
t
t
c
c
t
t
c
t
c
a
c
t
t
c
t
c
a
c
g
g
a
c
a

**8:75,272,349 A>G**

***GDAP1***

Squirls score:
**1.000**

The variant overlaps with 7
transcripts:

| Transcript Accession | CDS Change | Variant Effect | Squirls Score |
| --- | --- | --- | --- |
| *ENST00000220822.7* | c.311-23A>G | CODING\_TRANSCRIPT\_INTRON\_VARIANT | 1.000 |
| *ENST00000434412.2* | c.107-23A>G | CODING\_TRANSCRIPT\_INTRON\_VARIANT | 1.000 |
| *ENST00000520797.1* | n.76-23A>G | NON\_CODING\_TRANSCRIPT\_INTRON\_VARIANT | 1.000 |
| *ENST00000521096.1* | n.117-23A>G | NON\_CODING\_TRANSCRIPT\_INTRON\_VARIANT | 1.000 |
| *ENST00000522568.1* | c.166-23A>G | CODING\_TRANSCRIPT\_INTRON\_VARIANT | 1.000 |
| *ENST00000523640.1* | n.146-23A>G | NON\_CODING\_TRANSCRIPT\_INTRON\_VARIANT | 1.000 |
| *ENST00000524366.1* | n.329-1770A>G | NON\_CODING\_TRANSCRIPT\_INTRON\_VARIANT | 0.007 |

Squirls features

| Feature | Value |
| --- | --- |
| *ΔRi* canonical acceptor | 0.63 |
| *ΔRi* cryptic acceptor | 3.03 |
| Creates AG in *AGEZ* | Yes |
| Acceptor offset | -23 |
| Exon length | 112 |
| ESRSeq | -0.71 |
| SMS | -0.51 |
| phyloP | 1.39 |

Cryptic acceptor

Using cryptic acceptor site at *8:75,272,350*
would lead to addition
of 22
bases
to the coding sequence.

The variant creates *AG* dinucleotide in the
AG exclusion zone
of the canonical acceptor site.

Canonical acceptor site

G
C
A
T
G
A
C
T
G
A
C
T
G
A
C
T
G
A
C
T
G
A
C
T
G
A
C
T
G
A
C
T
G
A
C
T
A
G
C
T
A
G
C
T
A
G
C
T
A
G
C
T
A
G
C
T
A
G
C
T
A
G
C
T
A
G
C
T
G
A
C
T
G
A
C
T
G
A
C
T
G
A
C
T
G
A
C
T
G
A
T
C
C
T
G
A
A
T
C
G
T
C
A
G
C
G
A
T
-25
.
.
.
.
-20
.
.
.
.
-15
.
.
.
.
.
-9
-8
-7
-6
-5
-4
-3
-2
-1
1
2
c
a
a
t
a
t
t
t
g
t
g
t
g
t
g
t
g
t
a
t
t
t
t
a
g
a
a
g

Predicted cryptic acceptor site

G
C
A
T
G
A
C
T
G
A
C
T
G
A
C
T
G
A
C
T
G
A
C
T
G
A
C
T
G
A
C
T
G
A
C
T
A
G
C
T
A
G
C
T
A
G
C
T
A
G
C
T
A
G
C
T
A
G
C
T
A
G
C
T
A
G
C
T
G
A
C
T
G
A
C
T
G
A
C
T
G
A
C
T
G
A
C
T
G
A
T
C
C
T
G
A
A
T
C
G
T
C
A
G
C
G
A
T
-47
.
.
.
.
.
-42
.
.
.
.
.
-37
.
.
.
.
.
-32
.
.
.
.
.
-27
.
.
.
.
.
-22
.
.
a
a
c
t
c
a
t
g
t
g
t
a
a
c
t
t
t
t
t
c
t
t
c
a
a
t
a
g

**11:47,368,616 C>T**

***MYBPC3***

Squirls score:
**0.999**

The variant overlaps with 4
transcripts:

| Transcript Accession | CDS Change | Variant Effect | Squirls Score |
| --- | --- | --- | --- |
| *ENST00000256993.4* | c.905+361G>A | CODING\_TRANSCRIPT\_INTRON\_VARIANT | 0.008 |
| *ENST00000399249.2* | c.905+361G>A | CODING\_TRANSCRIPT\_INTRON\_VARIANT | 0.008 |
| *ENST00000544791.1* | c.906-36G>A | CODING\_TRANSCRIPT\_INTRON\_VARIANT | 0.999 |
| *ENST00000545968.1* | c.906-36G>A | CODING\_TRANSCRIPT\_INTRON\_VARIANT | 0.999 |

Squirls features

| Feature | Value |
| --- | --- |
| *ΔRi* canonical acceptor | 0.00 |
| *ΔRi* cryptic acceptor | 9.01 |
| Creates AG in *AGEZ* | Yes |
| Acceptor offset | -36 |
| Exon length | -1 |
| ESRSeq | -2.52 |
| SMS | 0.12 |
| phyloP | -0.26 |

Cryptic acceptor

Using cryptic acceptor site at *11:47,368,618*
would lead to addition
of 34
bases
to the coding sequence.

The variant creates *AG* dinucleotide in the
AG exclusion zone
of the canonical acceptor site.

Canonical acceptor site

G
C
A
T
G
A
C
T
G
A
C
T
G
A
C
T
G
A
C
T
G
A
C
T
G
A
C
T
G
A
C
T
G
A
C
T
A
G
C
T
A
G
C
T
A
G
C
T
A
G
C
T
A
G
C
T
A
G
C
T
A
G
C
T
A
G
C
T
G
A
C
T
G
A
C
T
G
A
C
T
G
A
C
T
G
A
C
T
G
A
T
C
C
T
G
A
A
T
C
G
T
C
A
G
C
G
A
T
-25
.
.
.
.
-20
.
.
.
.
-15
.
.
.
.
.
-9
-8
-7
-6
-5
-4
-3
-2
-1
1
2
t
a
t
g
g
c
t
g
c
t
g
c
t
g
c
t
g
t
g
g
c
c
c
a
g
a
g

Predicted cryptic acceptor site

G
C
A
T
G
A
C
T
G
A
C
T
G
A
C
T
G
A
C
T
G
A
C
T
G
A
C
T
G
A
C
T
G
A
C
T
A
G
C
T
A
G
C
T
A
G
C
T
A
G
C
T
A
G
C
T
A
G
C
T
A
G
C
T
A
G
C
T
G
A
C
T
G
A
C
T
G
A
C
T
G
A
C
T
G
A
C
T
G
A
T
C
C
T
G
A
A
T
C
G
T
C
A
G
C
G
A
T
-59
.
.
.
.
.
-54
.
.
.
.
.
-49
.
.
.
.
.
-44
.
.
.
.
.
-39
.
.
.
.
.
-34
.
.
t
g
c
c
c
t
c
t
c
t
c
t
c
c
c
t
c
t
c
c
c
c
c
g
g
c
c
a

**21:34,957,110 C>T**

***DONSON***

Squirls score:
**0.988**

The variant overlaps with 11
transcripts:

| Transcript Accession | CDS Change | Variant Effect | Squirls Score |
| --- | --- | --- | --- |
| *ENST00000303071.5* | c.607-36G>A | CODING\_TRANSCRIPT\_INTRON\_VARIANT | 0.988 |
| *ENST00000303113.6* | c.607-78G>A | CODING\_TRANSCRIPT\_INTRON\_VARIANT | 0.008 |
| *ENST00000417871.1* | c.\*75-36G>A | THREE\_PRIME\_UTR\_INTRON\_VARIANT | 0.988 |
| *ENST00000429238.1* | c.491-36G>A | CODING\_TRANSCRIPT\_INTRON\_VARIANT | 0.988 |
| *ENST00000432378.1* | c.607-36G>A | CODING\_TRANSCRIPT\_INTRON\_VARIANT | 0.988 |
| *ENST00000437395.1* | c.519-36G>A | CODING\_TRANSCRIPT\_INTRON\_VARIANT | 0.988 |
| *ENST00000440810.1* | c.182-36G>A | CODING\_TRANSCRIPT\_INTRON\_VARIANT | 0.988 |
| *ENST00000442660.1* | c.286-36G>A | CODING\_TRANSCRIPT\_INTRON\_VARIANT | 0.988 |
| *ENST00000444517.1* | c.286-36G>A | CODING\_TRANSCRIPT\_INTRON\_VARIANT | 0.988 |
| *ENST00000453626.1* | c.607-36G>A | CODING\_TRANSCRIPT\_INTRON\_VARIANT | 0.988 |
| *ENST00000457359.1* | c.607-1138G>A | CODING\_TRANSCRIPT\_INTRON\_VARIANT | 0.006 |

Squirls features

| Feature | Value |
| --- | --- |
| *ΔRi* canonical acceptor | 0.00 |
| *ΔRi* cryptic acceptor | 3.42 |
| Creates AG in *AGEZ* | Yes |
| Acceptor offset | -36 |
| Exon length | -1 |
| ESRSeq | -1.18 |
| SMS | -2.22 |
| phyloP | -0.17 |

Cryptic acceptor

Using cryptic acceptor site at *21:34,957,112*
would lead to addition
of 34
bases
to the coding sequence.

The variant creates *AG* dinucleotide in the
AG exclusion zone
of the canonical acceptor site.

Canonical acceptor site

G
C
A
T
G
A
C
T
G
A
C
T
G
A
C
T
G
A
C
T
G
A
C
T
G
A
C
T
G
A
C
T
G
A
C
T
A
G
C
T
A
G
C
T
A
G
C
T
A
G
C
T
A
G
C
T
A
G
C
T
A
G
C
T
A
G
C
T
G
A
C
T
G
A
C
T
G
A
C
T
G
A
C
T
G
A
C
T
G
A
T
C
C
T
G
A
A
T
C
G
T
C
A
G
C
G
A
T
-25
.
.
.
.
-20
.
.
.
.
-15
.
.
.
.
.
-9
-8
-7
-6
-5
-4
-3
-2
-1
1
2
g
c
c
c
a
c
t
g
t
c
a
c
c
t
t
t
g
g
a
t
t
t
t
a
g
g
a

Predicted cryptic acceptor site

G
C
A
T
G
A
C
T
G
A
C
T
G
A
C
T
G
A
C
T
G
A
C
T
G
A
C
T
G
A
C
T
G
A
C
T
A
G
C
T
A
G
C
T
A
G
C
T
A
G
C
T
A
G
C
T
A
G
C
T
A
G
C
T
A
G
C
T
G
A
C
T
G
A
C
T
G
A
C
T
G
A
C
T
G
A
C
T
G
A
T
C
C
T
G
A
A
T
C
G
T
C
A
G
C
G
A
T
-59
.
.
.
.
.
-54
.
.
.
.
.
-49
.
.
.
.
.
-44
.
.
.
.
.
-39
.
.
.
.
.
-34
.
.
a
a
t
a
a
a
a
t
t
c
t
c
t
a
t
t
t
c
t
c
a
a
t
g
g
g
c
a

**12:48,393,958 G>C**

***COL2A1***

Squirls score:
**0.977**

The variant overlaps with 4
transcripts:

| Transcript Accession | CDS Change | Variant Effect | Squirls Score |
| --- | --- | --- | --- |
| *ENST00000337299.6* | c.86-1744C>G | CODING\_TRANSCRIPT\_INTRON\_VARIANT | 0.006 |
| *ENST00000380518.3* | c.86-50C>G | CODING\_TRANSCRIPT\_INTRON\_VARIANT | 0.977 |
| *ENST00000474996.2* | n.324-50C>G | NON\_CODING\_TRANSCRIPT\_INTRON\_VARIANT | 0.977 |
| *ENST00000490609.1* | n.251-50C>G | NON\_CODING\_TRANSCRIPT\_INTRON\_VARIANT | 0.977 |

Squirls features

| Feature | Value |
| --- | --- |
| *ΔRi* canonical acceptor | 0.00 |
| *ΔRi* cryptic acceptor | 5.34 |
| Creates AG in *AGEZ* | Yes |
| Acceptor offset | -50 |
| Exon length | -1 |
| ESRSeq | -0.34 |
| SMS | 0.72 |
| phyloP | 0.80 |

Cryptic acceptor

Using cryptic acceptor site at *12:48,393,959*
would lead to addition
of 49
bases
to the coding sequence.

The variant creates *AG* dinucleotide in the
AG exclusion zone
of the canonical acceptor site.

Canonical acceptor site

G
C
A
T
G
A
C
T
G
A
C
T
G
A
C
T
G
A
C
T
G
A
C
T
G
A
C
T
G
A
C
T
G
A
C
T
A
G
C
T
A
G
C
T
A
G
C
T
A
G
C
T
A
G
C
T
A
G
C
T
A
G
C
T
A
G
C
T
G
A
C
T
G
A
C
T
G
A
C
T
G
A
C
T
G
A
C
T
G
A
T
C
C
T
G
A
A
T
C
G
T
C
A
G
C
G
A
T
-25
.
.
.
.
-20
.
.
.
.
-15
.
.
.
.
.
-9
-8
-7
-6
-5
-4
-3
-2
-1
1
2
t
t
c
t
c
c
c
t
c
c
c
c
a
c
c
c
t
t
g
g
t
g
c
a
g
a
g

Predicted cryptic acceptor site

G
C
A
T
G
A
C
T
G
A
C
T
G
A
C
T
G
A
C
T
G
A
C
T
G
A
C
T
G
A
C
T
G
A
C
T
A
G
C
T
A
G
C
T
A
G
C
T
A
G
C
T
A
G
C
T
A
G
C
T
A
G
C
T
A
G
C
T
G
A
C
T
G
A
C
T
G
A
C
T
G
A
C
T
G
A
C
T
G
A
T
C
C
T
G
A
A
T
C
G
T
C
A
G
C
G
A
T
-74
.
.
.
.
.
-69
.
.
.
.
.
-64
.
.
.
.
.
-59
.
.
.
.
.
-54
.
.
.
.
.
-49
.
.
t
c
t
c
t
t
t
t
t
c
t
c
t
c
t
g
g
c
t
c
c
c
c
a
c
c
c
g

**13:52,534,477 A>T**

***ATP7B***

Squirls score:
**0.751**

The variant overlaps with 9
transcripts:

| Transcript Accession | CDS Change | Variant Effect | Squirls Score |
| --- | --- | --- | --- |
| *ENST00000242839.4* | c.1947-19T>A | CODING\_TRANSCRIPT\_INTRON\_VARIANT | 0.751 |
| *ENST00000344297.5* | c.1870-2734T>A | CODING\_TRANSCRIPT\_INTRON\_VARIANT | 0.008 |
| *ENST00000400366.3* | c.1614-19T>A | CODING\_TRANSCRIPT\_INTRON\_VARIANT | 0.751 |
| *ENST00000400370.3* | c.1286-10180T>A | CODING\_TRANSCRIPT\_INTRON\_VARIANT | 0.008 |
| *ENST00000417240.2* | c.-64+1496T>A | FIVE\_PRIME\_UTR\_INTRON\_VARIANT | 0.009 |
| *ENST00000418097.2* | c.1947-19T>A | CODING\_TRANSCRIPT\_INTRON\_VARIANT | 0.751 |
| *ENST00000448424.2* | c.1947-19T>A | CODING\_TRANSCRIPT\_INTRON\_VARIANT | 0.751 |
| *ENST00000482841.1* | n.1426-2734T>A | NON\_CODING\_TRANSCRIPT\_INTRON\_VARIANT | 0.008 |
| *ENST00000542656.1* | c.1448-1797T>A | CODING\_TRANSCRIPT\_INTRON\_VARIANT | 0.007 |

Squirls features

| Feature | Value |
| --- | --- |
| *ΔRi* canonical acceptor | 1.18 |
| *ΔRi* cryptic acceptor | 0.00 |
| Creates AG in *AGEZ* | Yes |
| Acceptor offset | -19 |
| Exon length | 175 |
| ESRSeq | -1.91 |
| SMS | -2.33 |
| phyloP | 0.78 |

Canonical acceptor

Canonical acceptor site

G
C
A
T
G
A
C
T
G
A
C
T
G
A
C
T
G
A
C
T
G
A
C
T
G
A
C
T
G
A
C
T
G
A
C
T
A
G
C
T
A
G
C
T
A
G
C
T
A
G
C
T
A
G
C
T
A
G
C
T
A
G
C
T
A
G
C
T
G
A
C
T
G
A
C
T
G
A
C
T
G
A
C
T
G
A
C
T
G
A
T
C
C
T
G
A
A
T
C
G
T
C
A
G
C
G
A
T
-25
.
.
.
.
-20
.
.
.
.
-15
.
.
.
.
.
-9
-8
-7
-6
-5
-4
-3
-2
-1
1
2
c
t
g
t
g
t
t
g
c
t
g
c
a
t
t
t
g
c
t
t
t
c
c
a
g
g
t
a

*ΔRi* score distribution

-10


-5


0


5


10

ΔRi
: 1.18

**1:94,546,299 T>C**

***ABCA4***

Squirls score:
**0.733**

The variant overlaps with 2
transcripts:

| Transcript Accession | CDS Change | Variant Effect | Squirls Score |
| --- | --- | --- | --- |
| *ENST00000370225.3* | c.859-25A>G | CODING\_TRANSCRIPT\_INTRON\_VARIANT | 0.733 |
| *ENST00000535735.1* | c.859-25A>G | CODING\_TRANSCRIPT\_INTRON\_VARIANT | 0.733 |

Squirls features

| Feature | Value |
| --- | --- |
| *ΔRi* canonical acceptor | 0.64 |
| *ΔRi* cryptic acceptor | 0.00 |
| Creates AG in *AGEZ* | Yes |
| Acceptor offset | -25 |
| Exon length | 241 |
| ESRSeq | -1.25 |
| SMS | -0.56 |
| phyloP | 2.56 |

Canonical acceptor

Canonical acceptor site

G
C
A
T
G
A
C
T
G
A
C
T
G
A
C
T
G
A
C
T
G
A
C
T
G
A
C
T
G
A
C
T
G
A
C
T
A
G
C
T
A
G
C
T
A
G
C
T
A
G
C
T
A
G
C
T
A
G
C
T
A
G
C
T
A
G
C
T
G
A
C
T
G
A
C
T
G
A
C
T
G
A
C
T
G
A
C
T
G
A
T
C
C
T
G
A
A
T
C
G
T
C
A
G
C
G
A
T
-25
.
.
.
.
-20
.
.
.
.
-15
.
.
.
.
.
-9
-8
-7
-6
-5
-4
-3
-2
-1
1
2
a
c
c
t
g
a
a
g
a
c
t
t
t
g
c
c
t
t
g
c
c
c
t
a
g
t
t
g

*ΔRi* score distribution

-10


-5


0


5


10

ΔRi
: 0.64

**11:57,365,567 G>T**

***SERPING1***

Squirls score:
**0.716**

The variant overlaps with 7
transcripts:

| Transcript Accession | CDS Change | Variant Effect | Squirls Score |
| --- | --- | --- | --- |
| *ENST00000278407.4* | c.-22-155G>T | FIVE\_PRIME\_UTR\_INTRON\_VARIANT | 0.663 |
| *ENST00000340687.6* | c.-22-155G>T | FIVE\_PRIME\_UTR\_INTRON\_VARIANT | 0.663 |
| *ENST00000378323.4* | c.-22-155G>T | FIVE\_PRIME\_UTR\_INTRON\_VARIANT | 0.663 |
| *ENST00000378324.2* | c.-106+372G>T | FIVE\_PRIME\_UTR\_INTRON\_VARIANT | 0.419 |
| *ENST00000403558.1* | c.-75G>T | FIVE\_PRIME\_UTR\_EXON\_VARIANT | 0.367 |
| *ENST00000405496.1* | c.-22-155G>T | FIVE\_PRIME\_UTR\_INTRON\_VARIANT | 0.716 |
| *ENST00000457869.1* | c.-75G>T | FIVE\_PRIME\_UTR\_EXON\_VARIANT | 0.338 |

Squirls features

| Feature | Value |
| --- | --- |
| *Ri wt* donor | 5.00 |
| *ΔRi* canonical donor | 0.00 |
| *ΔRi wt* closest donor | 0.00 |
| Donor offset | -228 |
| max *Ri* cryptic donor window | 7.95 |
| *ΔRi* cryptic donor | 2.95 |
| phyloP | 5.07 |

Cryptic donor

Using cryptic donor site at *11:57,365,566*
would lead to addition
of 562
bases
to the coding sequence.

Canonical donor site

T
G
A
C
C
G
T
A
C
T
A
G
C
T
A
G
A
G
C
T
C
T
G
A
C
T
G
A
C
T
A
G
C
A
G
T
-3

-2

-1

1

2

3

4

5

6

g
c
g
g
t
c
a
g
t

Predicted cryptic donor site

T
G
A
C
C
G
T
A
C
T
A
G
C
T
A
G
A
G
C
T
C
T
G
A
C
T
G
A
C
T
A
G
C
A
G
T
560

.

.

.

563

.

.

.

.

.

568

.

g
a
g
g
g
a
g
g
a
t

**X:32,366,860 A>C**

***DMD***

Squirls score:
**0.658**

The variant overlaps with 3
transcripts:

| Transcript Accession | CDS Change | Variant Effect | Squirls Score |
| --- | --- | --- | --- |
| *ENST00000357033.4* | c.5326-215T>G | CODING\_TRANSCRIPT\_INTRON\_VARIANT | 0.658 |
| *ENST00000378677.2* | c.5314-215T>G | CODING\_TRANSCRIPT\_INTRON\_VARIANT | 0.658 |
| *ENST00000488902.1* | n.336-131680T>G | NON\_CODING\_TRANSCRIPT\_INTRON\_VARIANT | 0.296 |

Squirls features

| Feature | Value |
| --- | --- |
| *Ri wt* donor | 8.79 |
| *ΔRi* canonical donor | 0.00 |
| *ΔRi wt* closest donor | 0.00 |
| Donor offset | -338 |
| max *Ri* cryptic donor window | 10.24 |
| *ΔRi* cryptic donor | 1.46 |
| phyloP | 3.57 |

Cryptic donor

Using cryptic donor site at *X:32,366,861*
would lead to addition
of 14046
bases
to the coding sequence.

Canonical donor site

T
G
A
C
C
G
T
A
C
T
A
G
C
T
A
G
A
G
C
T
C
T
G
A
C
T
G
A
C
T
A
G
C
A
G
T
-3

-2

-1

1

2

3

4

5

6

a
a
g
g
t
a
g
g
a

Predicted cryptic donor site

T
G
A
C
C
G
T
A
C
T
A
G
C
T
A
G
A
G
C
T
C
T
G
A
C
T
G
A
C
T
A
G
C
A
G
T
14044

.

.

.

14047

.

.

.

.

.

14052

.

c
a
t
g
t
a
t
g
t
g

**1:216,064,540 T>C**

***USH2A***

Squirls score:
**0.529**

The variant overlaps with 2
transcripts:

| Transcript Accession | CDS Change | Variant Effect | Squirls Score |
| --- | --- | --- | --- |
| *ENST00000307340.3* | c.7595-2144A>G | CODING\_TRANSCRIPT\_INTRON\_VARIANT | 0.529 |
| *ENST00000366943.2* | c.7595-2144A>G | CODING\_TRANSCRIPT\_INTRON\_VARIANT | 0.529 |

Squirls features

| Feature | Value |
| --- | --- |
| *Ri wt* donor | 7.05 |
| *ΔRi* canonical donor | 0.00 |
| *ΔRi wt* closest donor | 0.00 |
| Donor offset | -2773 |
| max *Ri* cryptic donor window | 11.27 |
| *ΔRi* cryptic donor | 4.23 |
| phyloP | 1.95 |

Cryptic donor

Using cryptic donor site at *1:216,064,540*
would lead to addition
of 8877
bases
to the coding sequence.

Canonical donor site

T
G
A
C
C
G
T
A
C
T
A
G
C
T
A
G
A
G
C
T
C
T
G
A
C
T
G
A
C
T
A
G
C
A
G
T
-3

-2

-1

1

2

3

4

5

6

a
c
a
g
t
a
a
g
t

Predicted cryptic donor site

T
G
A
C
C
G
T
A
C
T
A
G
C
T
A
G
A
G
C
T
C
T
G
A
C
T
G
A
C
T
A
G
C
A
G
T
8875

.

.

.

8878

.

.

.

.

.

8883

.

a
a
g
a
t
a
a
g
a
g

**11:66,618,867 G>A**

***PC***

Squirls score:
**0.437**

The variant overlaps with 4
transcripts:

| Transcript Accession | CDS Change | Variant Effect | Squirls Score |
| --- | --- | --- | --- |
| *ENST00000393955.2* | c.1983-116C>T | CODING\_TRANSCRIPT\_INTRON\_VARIANT | 0.437 |
| *ENST00000393958.2* | c.1983-116C>T | CODING\_TRANSCRIPT\_INTRON\_VARIANT | 0.437 |
| *ENST00000393960.1* | c.1983-116C>T | CODING\_TRANSCRIPT\_INTRON\_VARIANT | 0.437 |
| *ENST00000530259.1* | n.596-116C>T | NON\_CODING\_TRANSCRIPT\_INTRON\_VARIANT | 0.437 |

Squirls features

| Feature | Value |
| --- | --- |
| *Ri wt* donor | 7.28 |
| *ΔRi* canonical donor | 0.00 |
| *ΔRi wt* closest donor | 0.00 |
| Donor offset | -357 |
| max *Ri* cryptic donor window | 10.57 |
| *ΔRi* cryptic donor | 3.29 |
| phyloP | 0.83 |

Cryptic donor

Using cryptic donor site at *11:66,618,866*
would lead to addition
of 393
bases
to the coding sequence.

Canonical donor site

T
G
A
C
C
G
T
A
C
T
A
G
C
T
A
G
A
G
C
T
C
T
G
A
C
T
G
A
C
T
A
G
C
A
G
T
-3

-2

-1

1

2

3

4

5

6

c
a
a
g
t
g
a
g
c

Predicted cryptic donor site

T
G
A
C
C
G
T
A
C
T
A
G
C
T
A
G
A
G
C
T
C
T
G
A
C
T
G
A
C
T
A
G
C
A
G
T
391

.

.

.

394

.

.

.

.

.

399

.

c
a
g
g
c
g
a
g
a
t

**X:31,899,369 T>C**

***DMD***

Squirls score:
**0.412**

The variant overlaps with 7
transcripts:

| Transcript Accession | CDS Change | Variant Effect | Squirls Score |
| --- | --- | --- | --- |
| *ENST00000343523.2* | c.-468-5879A>G | FIVE\_PRIME\_UTR\_INTRON\_VARIANT | 0.412 |
| *ENST00000357033.4* | c.6913-5879A>G | CODING\_TRANSCRIPT\_INTRON\_VARIANT | 0.412 |
| *ENST00000359836.1* | c.-468-5879A>G | FIVE\_PRIME\_UTR\_INTRON\_VARIANT | 0.412 |
| *ENST00000378677.2* | c.6901-5879A>G | CODING\_TRANSCRIPT\_INTRON\_VARIANT | 0.412 |
| *ENST00000378707.3* | c.-468-5879A>G | FIVE\_PRIME\_UTR\_INTRON\_VARIANT | 0.412 |
| *ENST00000474231.1* | c.-468-5879A>G | FIVE\_PRIME\_UTR\_INTRON\_VARIANT | 0.412 |
| *ENST00000541735.1* | c.-468-5879A>G | FIVE\_PRIME\_UTR\_INTRON\_VARIANT | 0.412 |

Squirls features

| Feature | Value |
| --- | --- |
| *Ri wt* donor | 7.10 |
| *ΔRi* canonical donor | 0.00 |
| *ΔRi wt* closest donor | 0.00 |
| Donor offset | -6065 |
| max *Ri* cryptic donor window | 10.49 |
| *ΔRi* cryptic donor | 3.40 |
| phyloP | 0.29 |

Cryptic donor

Using cryptic donor site at *X:31,899,369*
would lead to addition
of 48344
bases
to the coding sequence.

Canonical donor site

T
G
A
C
C
G
T
A
C
T
A
G
C
T
A
G
A
G
C
T
C
T
G
A
C
T
G
A
C
T
A
G
C
A
G
T
-3

-2

-1

1

2

3

4

5

6

a
a
g
g
t
t
a
g
a

Predicted cryptic donor site

T
G
A
C
C
G
T
A
C
T
A
G
C
T
A
G
A
G
C
T
C
T
G
A
C
T
G
A
C
T
A
G
C
A
G
T
48342

.

.

.

48345

.

.

.

.

.

48350

.

a
t
g
a
t
a
a
g
t
g

**1:98,045,449 G>C**

***DPYD***

Squirls score:
**0.394**

The variant overlaps with 1
transcript:

| Transcript Accession | CDS Change | Variant Effect | Squirls Score |
| --- | --- | --- | --- |
| *ENST00000370192.3* | c.1129-5923C>G | CODING\_TRANSCRIPT\_INTRON\_VARIANT | 0.394 |

Squirls features

| Feature | Value |
| --- | --- |
| *Ri wt* donor | 7.35 |
| *ΔRi* canonical donor | 0.00 |
| *ΔRi wt* closest donor | 0.00 |
| Donor offset | -6134 |
| max *Ri* cryptic donor window | 10.46 |
| *ΔRi* cryptic donor | 3.10 |
| phyloP | 0.09 |

Cryptic donor

Using cryptic donor site at *1:98,045,449*
would lead to addition
of 13325
bases
to the coding sequence.

Canonical donor site

T
G
A
C
C
G
T
A
C
T
A
G
C
T
A
G
A
G
C
T
C
T
G
A
C
T
G
A
C
T
A
G
C
A
G
T
-3

-2

-1

1

2

3

4

5

6

g
a
g
g
t
a
a
a
a

Predicted cryptic donor site

T
G
A
C
C
G
T
A
C
T
A
G
C
T
A
G
A
G
C
T
C
T
G
A
C
T
G
A
C
T
A
G
C
A
G
T
13323

.

.

.

13326

.

.

.

.

.

13331

.

a
a
g
c
t
g
a
g
a
g

**6:76,593,963 T>G**

***MYO6***

Squirls score:
**0.381**

The variant overlaps with 4
transcripts:

| Transcript Accession | CDS Change | Variant Effect | Squirls Score |
| --- | --- | --- | --- |
| *ENST00000369975.1* | c.2417-1758T>G | CODING\_TRANSCRIPT\_INTRON\_VARIANT | 0.381 |
| *ENST00000369977.3* | c.2417-1758T>G | CODING\_TRANSCRIPT\_INTRON\_VARIANT | 0.381 |
| *ENST00000369981.3* | c.2417-1758T>G | CODING\_TRANSCRIPT\_INTRON\_VARIANT | 0.381 |
| *ENST00000369985.4* | c.2417-1758T>G | CODING\_TRANSCRIPT\_INTRON\_VARIANT | 0.381 |

Squirls features

| Feature | Value |
| --- | --- |
| *Ri wt* donor | 6.66 |
| *ΔRi* canonical donor | 0.00 |
| *ΔRi wt* closest donor | 0.00 |
| Donor offset | -1849 |
| max *Ri* cryptic donor window | 12.69 |
| *ΔRi* cryptic donor | 6.03 |
| phyloP | 0.12 |

Cryptic donor

Using cryptic donor site at *6:76,593,963*
would lead to addition
of 2428
bases
to the coding sequence.

Canonical donor site

T
G
A
C
C
G
T
A
C
T
A
G
C
T
A
G
A
G
C
T
C
T
G
A
C
T
G
A
C
T
A
G
C
A
G
T
-3

-2

-1

1

2

3

4

5

6

a
a
t
g
t
a
g
g
t

Predicted cryptic donor site

T
G
A
C
C
G
T
A
C
T
A
G
C
T
A
G
A
G
C
T
C
T
G
A
C
T
G
A
C
T
A
G
C
A
G
T
2426

.

.

.

2429

.

.

.

.

.

2434

.

a
a
g
t
t
a
a
g
t
g

**X:37,657,051 A>G**

***CYBB***

Squirls score:
**0.367**

The variant overlaps with 4
transcripts:

| Transcript Accession | CDS Change | Variant Effect | Squirls Score |
| --- | --- | --- | --- |
| *ENST00000378588.4* | c.675-1157A>G | CODING\_TRANSCRIPT\_INTRON\_VARIANT | 0.367 |
| *ENST00000465127.1* | c.171+371798A>G | CODING\_TRANSCRIPT\_INTRON\_VARIANT | 0.079 |
| *ENST00000536160.1* | c.-127-1157A>G | FIVE\_PRIME\_UTR\_INTRON\_VARIANT | 0.117 |
| *ENST00000545017.1* | c.579-1157A>G | CODING\_TRANSCRIPT\_INTRON\_VARIANT | 0.367 |

Squirls features

| Feature | Value |
| --- | --- |
| *Ri wt* donor | 4.94 |
| *ΔRi* canonical donor | 0.00 |
| *ΔRi wt* closest donor | 0.00 |
| Donor offset | -1287 |
| max *Ri* cryptic donor window | 9.28 |
| *ΔRi* cryptic donor | 4.34 |
| phyloP | 0.99 |

Cryptic donor

Using cryptic donor site at *X:37,657,052*
would lead to addition
of 1658
bases
to the coding sequence.

Canonical donor site

T
G
A
C
C
G
T
A
C
T
A
G
C
T
A
G
A
G
C
T
C
T
G
A
C
T
G
A
C
T
A
G
C
A
G
T
-3

-2

-1

1

2

3

4

5

6

t
g
a
g
t
g
a
g
t

Predicted cryptic donor site

T
G
A
C
C
G
T
A
C
T
A
G
C
T
A
G
A
G
C
T
C
T
G
A
C
T
G
A
C
T
A
G
C
A
G
T
1656

.

.

.

1659

.

.

.

.

.

1664

.

c
t
a
g
t
a
a
g
g
g

**10:126,100,239 G>C**

***OAT***

Squirls score:
**0.356**

The variant overlaps with 5
transcripts:

| Transcript Accession | CDS Change | Variant Effect | Squirls Score |
| --- | --- | --- | --- |
| *ENST00000368845.5* | c.199+303C>G | CODING\_TRANSCRIPT\_INTRON\_VARIANT | 0.031 |
| *ENST00000476917.1* | n.264+303C>G | NON\_CODING\_TRANSCRIPT\_INTRON\_VARIANT | 0.031 |
| *ENST00000490096.1* | n.435+303C>G | NON\_CODING\_TRANSCRIPT\_INTRON\_VARIANT | 0.031 |
| *ENST00000492376.1* | n.547+303C>G | NON\_CODING\_TRANSCRIPT\_INTRON\_VARIANT | 0.031 |
| *ENST00000539214.1* | c.-215-2705C>G | FIVE\_PRIME\_UTR\_INTRON\_VARIANT | 0.356 |

Squirls features

| Feature | Value |
| --- | --- |
| *Ri wt* donor | 4.66 |
| *ΔRi* canonical donor | 0.00 |
| *ΔRi wt* closest donor | 0.00 |
| Donor offset | -2930 |
| max *Ri* cryptic donor window | 9.52 |
| *ΔRi* cryptic donor | 4.86 |
| phyloP | -0.05 |

Cryptic donor

Using cryptic donor site at *10:126,100,239*
would lead to addition
of 7203
bases
to the coding sequence.

Canonical donor site

T
G
A
C
C
G
T
A
C
T
A
G
C
T
A
G
A
G
C
T
C
T
G
A
C
T
G
A
C
T
A
G
C
A
G
T
-3

-2

-1

1

2

3

4

5

6

c
a
g
g
t
a
c
c
g

Predicted cryptic donor site

T
G
A
C
C
G
T
A
C
T
A
G
C
T
A
G
A
G
C
T
C
T
G
A
C
T
G
A
C
T
A
G
C
A
G
T
7201

.

.

.

7204

.

.

.

.

.

7209

.

c
a
g
c
t
a
a
t
t
g

**2:71,900,503 C>T**

***DYSF***

Squirls score:
**0.354**

The variant overlaps with 12
transcripts:

| Transcript Accession | CDS Change | Variant Effect | Squirls Score |
| --- | --- | --- | --- |
| *ENST00000258104.3* | c.5668-824C>T | CODING\_TRANSCRIPT\_INTRON\_VARIANT | 0.354 |
| *ENST00000394120.2* | c.5671-824C>T | CODING\_TRANSCRIPT\_INTRON\_VARIANT | 0.354 |
| *ENST00000409366.1* | c.5734-824C>T | CODING\_TRANSCRIPT\_INTRON\_VARIANT | 0.354 |
| *ENST00000409582.3* | c.5782-824C>T | CODING\_TRANSCRIPT\_INTRON\_VARIANT | 0.354 |
| *ENST00000409651.1* | c.5764-824C>T | CODING\_TRANSCRIPT\_INTRON\_VARIANT | 0.354 |
| *ENST00000409744.1* | c.5692-824C>T | CODING\_TRANSCRIPT\_INTRON\_VARIANT | 0.354 |
| *ENST00000409762.1* | c.5719-824C>T | CODING\_TRANSCRIPT\_INTRON\_VARIANT | 0.354 |
| *ENST00000410020.3* | c.5785-824C>T | CODING\_TRANSCRIPT\_INTRON\_VARIANT | 0.354 |
| *ENST00000410041.1* | c.5722-824C>T | CODING\_TRANSCRIPT\_INTRON\_VARIANT | 0.354 |
| *ENST00000413539.2* | c.5761-824C>T | CODING\_TRANSCRIPT\_INTRON\_VARIANT | 0.354 |
| *ENST00000429174.2* | c.5731-824C>T | CODING\_TRANSCRIPT\_INTRON\_VARIANT | 0.354 |
| *ENST00000479049.2* | n.2553-824C>T | NON\_CODING\_TRANSCRIPT\_INTRON\_VARIANT | 0.354 |

Squirls features

| Feature | Value |
| --- | --- |
| *Ri wt* donor | 8.42 |
| *ΔRi* canonical donor | 0.00 |
| *ΔRi wt* closest donor | 0.00 |
| Donor offset | -924 |
| max *Ri* cryptic donor window | 10.33 |
| *ΔRi* cryptic donor | 1.91 |
| phyloP | -0.82 |

Cryptic donor

Using cryptic donor site at *2:71,900,502*
would lead to addition
of 3626
bases
to the coding sequence.

Canonical donor site

T
G
A
C
C
G
T
A
C
T
A
G
C
T
A
G
A
G
C
T
C
T
G
A
C
T
G
A
C
T
A
G
C
A
G
T
-3

-2

-1

1

2

3

4

5

6

a
a
g
g
t
c
a
g
t

Predicted cryptic donor site

T
G
A
C
C
G
T
A
C
T
A
G
C
T
A
G
A
G
C
T
C
T
G
A
C
T
G
A
C
T
A
G
C
A
G
T
3624

.

.

.

3627

.

.

.

.

.

3632

.

c
a
g
g
c
g
a
g
c
t

**X:32,460,274 G>T**

***DMD***

Squirls score:
**0.354**

The variant overlaps with 5
transcripts:

| Transcript Accession | CDS Change | Variant Effect | Squirls Score |
| --- | --- | --- | --- |
| *ENST00000357033.4* | c.3787-843C>A | CODING\_TRANSCRIPT\_INTRON\_VARIANT | 0.354 |
| *ENST00000378677.2* | c.3775-843C>A | CODING\_TRANSCRIPT\_INTRON\_VARIANT | 0.354 |
| *ENST00000420596.1* | c.94-76958C>A | CODING\_TRANSCRIPT\_INTRON\_VARIANT | 0.254 |
| *ENST00000448370.1* | c.94-77447C>A | CODING\_TRANSCRIPT\_INTRON\_VARIANT | 0.314 |
| *ENST00000488902.1* | n.336-225094C>A | NON\_CODING\_TRANSCRIPT\_INTRON\_VARIANT | 0.254 |

Squirls features

| Feature | Value |
| --- | --- |
| *Ri wt* donor | 4.53 |
| *ΔRi* canonical donor | 0.00 |
| *ΔRi wt* closest donor | 0.00 |
| Donor offset | -978 |
| max *Ri* cryptic donor window | 10.10 |
| *ΔRi* cryptic donor | 5.57 |
| phyloP | -0.70 |

Cryptic donor

Using cryptic donor site at *X:32,460,272*
would lead to addition
of 6297
bases
to the coding sequence.

Canonical donor site

T
G
A
C
C
G
T
A
C
T
A
G
C
T
A
G
A
G
C
T
C
T
G
A
C
T
G
A
C
T
A
G
C
A
G
T
-3

-2

-1

1

2

3

4

5

6

g
a
a
g
t
c
a
g
t

Predicted cryptic donor site

T
G
A
C
C
G
T
A
C
T
A
G
C
T
A
G
A
G
C
T
C
T
G
A
C
T
G
A
C
T
A
G
C
A
G
T
6295

.

.

.

6298

.

.

.

.

.

6303

.

a
c
g
g
t
c
a
g
t
a

**11:17,465,872 T>C**

***ABCC8***

Squirls score:
**0.350**

The variant overlaps with 4
transcripts:

| Transcript Accession | CDS Change | Variant Effect | Squirls Score |
| --- | --- | --- | --- |
| *ENST00000302539.4* | c.1333-1013A>G | CODING\_TRANSCRIPT\_INTRON\_VARIANT | 0.350 |
| *ENST00000389817.3* | c.1333-1013A>G | CODING\_TRANSCRIPT\_INTRON\_VARIANT | 0.350 |
| *ENST00000527905.1* | c.1333-1013A>G | CODING\_TRANSCRIPT\_INTRON\_VARIANT | 0.350 |
| *ENST00000532728.1* | n.1364-1013A>G | NON\_CODING\_TRANSCRIPT\_INTRON\_VARIANT | 0.350 |

Squirls features

| Feature | Value |
| --- | --- |
| *Ri wt* donor | 4.99 |
| *ΔRi* canonical donor | 0.00 |
| *ΔRi wt* closest donor | 0.00 |
| Donor offset | -1148 |
| max *Ri* cryptic donor window | 11.85 |
| *ΔRi* cryptic donor | 6.87 |
| phyloP | -1.05 |

Cryptic donor

Using cryptic donor site at *11:17,465,872*
would lead to addition
of 4191
bases
to the coding sequence.

Canonical donor site

T
G
A
C
C
G
T
A
C
T
A
G
C
T
A
G
A
G
C
T
C
T
G
A
C
T
G
A
C
T
A
G
C
A
G
T
-3

-2

-1

1

2

3

4

5

6

c
a
g
g
t
a
c
t
a

Predicted cryptic donor site

T
G
A
C
C
G
T
A
C
T
A
G
C
T
A
G
A
G
C
T
C
T
G
A
C
T
G
A
C
T
A
G
C
A
G
T
4189

.

.

.

4192

.

.

.

.

.

4197

.

g
a
g
a
t
a
a
g
t
g

**19:1,386,643 C>G**

***NDUFS7***

Squirls score:
**0.333**

The variant overlaps with 12
transcripts:

| Transcript Accession | CDS Change | Variant Effect | Squirls Score |
| --- | --- | --- | --- |
| *ENST00000233627.9* | c.17-1167C>G | CODING\_TRANSCRIPT\_INTRON\_VARIANT | 0.270 |
| *ENST00000313408.7* | c.17-1167C>G | CODING\_TRANSCRIPT\_INTRON\_VARIANT | 0.270 |
| *ENST00000414651.2* | c.17-1167C>G | CODING\_TRANSCRIPT\_INTRON\_VARIANT | 0.270 |
| *ENST00000436115.2* | n.40-1167C>G | NON\_CODING\_TRANSCRIPT\_INTRON\_VARIANT | 0.270 |
| *ENST00000534853.1* | c.12-1167C>G | CODING\_TRANSCRIPT\_INTRON\_VARIANT | 0.229 |
| *ENST00000538662.1* | n.44-1167C>G | NON\_CODING\_TRANSCRIPT\_INTRON\_VARIANT | 0.270 |
| *ENST00000538929.1* | n.107-1167C>G | NON\_CODING\_TRANSCRIPT\_INTRON\_VARIANT | 0.333 |
| *ENST00000539480.1* | c.17-1167C>G | CODING\_TRANSCRIPT\_INTRON\_VARIANT | 0.270 |
| *ENST00000543289.1* | n.268-1167C>G | NON\_CODING\_TRANSCRIPT\_INTRON\_VARIANT | 0.333 |
| *ENST00000545446.1* | n.60-1167C>G | NON\_CODING\_TRANSCRIPT\_INTRON\_VARIANT | 0.270 |
| *ENST00000546172.3* | c.\*12+5C>G | SPLICE\_REGION\_VARIANT | 0.008 |
| *ENST00000546283.1* | c.17-1167C>G | CODING\_TRANSCRIPT\_INTRON\_VARIANT | 0.270 |

Squirls features

| Feature | Value |
| --- | --- |
| *Ri wt* donor | 7.73 |
| *ΔRi* canonical donor | 0.00 |
| *ΔRi wt* closest donor | 0.00 |
| Donor offset | -1204 |
| max *Ri* cryptic donor window | 9.69 |
| *ΔRi* cryptic donor | 1.96 |
| phyloP | -1.56 |

Cryptic donor

Using cryptic donor site at *19:1,386,639*
would lead to addition
of 2484
bases
to the coding sequence.

Canonical donor site

T
G
A
C
C
G
T
A
C
T
A
G
C
T
A
G
A
G
C
T
C
T
G
A
C
T
G
A
C
T
A
G
C
A
G
T
-3

-2

-1

1

2

3

4

5

6

c
c
g
g
t
a
g
g
t

Predicted cryptic donor site

T
G
A
C
C
G
T
A
C
T
A
G
C
T
A
G
A
G
C
T
C
T
G
A
C
T
G
A
C
T
A
G
C
A
G
T
2482

.

.

.

2485

.

.

.

.

.

2490

.

c
a
g
g
t
a
c
c
t
g

**7:117,280,015 C>T**

***CFTR***

Squirls score:
**0.310**

The variant overlaps with 3
transcripts:

| Transcript Accession | CDS Change | Variant Effect | Squirls Score |
| --- | --- | --- | --- |
| *ENST00000003084.6* | c.3718-2477C>T | CODING\_TRANSCRIPT\_INTRON\_VARIANT | 0.310 |
| *ENST00000426809.1* | c.3628-2477C>T | CODING\_TRANSCRIPT\_INTRON\_VARIANT | 0.310 |
| *ENST00000454343.1* | c.3535-2477C>T | CODING\_TRANSCRIPT\_INTRON\_VARIANT | 0.310 |

Squirls features

| Feature | Value |
| --- | --- |
| *Ri wt* donor | 8.01 |
| *ΔRi* canonical donor | 0.00 |
| *ΔRi wt* closest donor | 0.00 |
| Donor offset | -2633 |
| max *Ri* cryptic donor window | 9.67 |
| *ΔRi* cryptic donor | 1.67 |
| phyloP | -0.59 |

Cryptic donor

Using cryptic donor site at *7:117,280,014*
would lead to addition
of 12190
bases
to the coding sequence.

Canonical donor site

T
G
A
C
C
G
T
A
C
T
A
G
C
T
A
G
A
G
C
T
C
T
G
A
C
T
G
A
C
T
A
G
C
A
G
T
-3

-2

-1

1

2

3

4

5

6

a
g
g
g
t
g
a
g
a

Predicted cryptic donor site

T
G
A
C
C
G
T
A
C
T
A
G
C
T
A
G
A
G
C
T
C
T
G
A
C
T
G
A
C
T
A
G
C
A
G
T
12188

.

.

.

12191

.

.

.

.

.

12196

.

a
t
g
g
c
g
a
g
t
t

**X:32,477,825 C>A**

***DMD***

Squirls score:
**0.310**

The variant overlaps with 5
transcripts:

| Transcript Accession | CDS Change | Variant Effect | Squirls Score |
| --- | --- | --- | --- |
| *ENST00000357033.4* | c.3432+3731G>T | CODING\_TRANSCRIPT\_INTRON\_VARIANT | 0.154 |
| *ENST00000378677.2* | c.3420+3731G>T | CODING\_TRANSCRIPT\_INTRON\_VARIANT | 0.154 |
| *ENST00000420596.1* | c.94-94509G>T | CODING\_TRANSCRIPT\_INTRON\_VARIANT | 0.310 |
| *ENST00000448370.1* | c.94-94998G>T | CODING\_TRANSCRIPT\_INTRON\_VARIANT | 0.309 |
| *ENST00000488902.1* | n.336-242645G>T | NON\_CODING\_TRANSCRIPT\_INTRON\_VARIANT | 0.310 |

Squirls features

| Feature | Value |
| --- | --- |
| *Ri wt* donor | 11.27 |
| *ΔRi* canonical donor | 0.00 |
| *ΔRi wt* closest donor | 0.00 |
| Donor offset | -242793 |
| max *Ri* cryptic donor window | 10.44 |
| *ΔRi* cryptic donor | -0.84 |
| phyloP | -0.15 |

Cryptic donor

Using cryptic donor site at *X:32,477,824*
would lead to addition
of 560430
bases
to the coding sequence.

Canonical donor site

T
G
A
C
C
G
T
A
C
T
A
G
C
T
A
G
A
G
C
T
C
T
G
A
C
T
G
A
C
T
A
G
C
A
G
T
-3

-2

-1

1

2

3

4

5

6

a
a
g
g
t
a
a
g
a

Predicted cryptic donor site

T
G
A
C
C
G
T
A
C
T
A
G
C
T
A
G
A
G
C
T
C
T
G
A
C
T
G
A
C
T
A
G
C
A
G
T
560428

.

.

.

560431

.

.

.

.

.

560436

.

g
a
g
g
g
a
a
g
a
t

**16:8,926,102 C>T**

***PMM2***

Squirls score:
**0.303**

The variant overlaps with 11
transcripts:

| Transcript Accession | CDS Change | Variant Effect | Squirls Score |
| --- | --- | --- | --- |
| *ENST00000268261.4* | c.640-15479C>T | CODING\_TRANSCRIPT\_INTRON\_VARIANT | 0.303 |
| *ENST00000537352.1* | c.265-15479C>T | CODING\_TRANSCRIPT\_INTRON\_VARIANT | 0.303 |
| *ENST00000539622.1* | c.391-15479C>T | CODING\_TRANSCRIPT\_INTRON\_VARIANT | 0.303 |
| *ENST00000562318.1* | c.\*362-15479C>T | THREE\_PRIME\_UTR\_INTRON\_VARIANT | 0.303 |
| *ENST00000565221.1* | c.\*258-15479C>T | THREE\_PRIME\_UTR\_INTRON\_VARIANT | 0.303 |
| *ENST00000566540.1* | c.\*262-15479C>T | THREE\_PRIME\_UTR\_INTRON\_VARIANT | 0.303 |
| *ENST00000566604.1* | c.\*180-15479C>T | THREE\_PRIME\_UTR\_INTRON\_VARIANT | 0.303 |
| *ENST00000566983.1* | c.559-15479C>T | CODING\_TRANSCRIPT\_INTRON\_VARIANT | 0.303 |
| *ENST00000567697.1* | n.3808-15479C>T | NON\_CODING\_TRANSCRIPT\_INTRON\_VARIANT | 0.303 |
| *ENST00000569958.1* | c.367-15479C>T | CODING\_TRANSCRIPT\_INTRON\_VARIANT | 0.303 |
| *ENST00000570076.1* | c.\*98-15479C>T | THREE\_PRIME\_UTR\_INTRON\_VARIANT | 0.303 |

Squirls features

| Feature | Value |
| --- | --- |
| *Ri wt* donor | 7.16 |
| *ΔRi* canonical donor | 0.00 |
| *ΔRi wt* closest donor | 0.00 |
| Donor offset | -15880 |
| max *Ri* cryptic donor window | 9.37 |
| *ΔRi* cryptic donor | 2.21 |
| phyloP | -0.05 |

Cryptic donor

Using cryptic donor site at *16:8,926,101*
would lead to addition
of 19138
bases
to the coding sequence.

Canonical donor site

T
G
A
C
C
G
T
A
C
T
A
G
C
T
A
G
A
G
C
T
C
T
G
A
C
T
G
A
C
T
A
G
C
A
G
T
-3

-2

-1

1

2

3

4

5

6

c
c
a
g
t
a
a
g
t

Predicted cryptic donor site

T
G
A
C
C
G
T
A
C
T
A
G
C
T
A
G
A
G
C
T
C
T
G
A
C
T
G
A
C
T
A
G
C
A
G
T
19136

.

.

.

19139

.

.

.

.

.

19144

.

g
a
g
g
c
a
g
g
t
t

**15:91,337,139 A>G**

***BLM***

Squirls score:
**0.286**

The variant overlaps with 6
transcripts:

| Transcript Accession | CDS Change | Variant Effect | Squirls Score |
| --- | --- | --- | --- |
| *ENST00000355112.3* | c.3020-258A>G | CODING\_TRANSCRIPT\_INTRON\_VARIANT | 0.286 |
| *ENST00000558825.1* | n.109A>G | NON\_CODING\_TRANSCRIPT\_EXON\_VARIANT | 0.189 |
| *ENST00000559724.1* | c.\*1944-258A>G | THREE\_PRIME\_UTR\_INTRON\_VARIANT | 0.286 |
| *ENST00000560136.1* | n.1046-258A>G | NON\_CODING\_TRANSCRIPT\_INTRON\_VARIANT | 0.286 |
| *ENST00000560509.1* | c.3020-258A>G | CODING\_TRANSCRIPT\_INTRON\_VARIANT | 0.286 |
| *ENST00000560559.1* | n.557-258A>G | NON\_CODING\_TRANSCRIPT\_INTRON\_VARIANT | 0.286 |

Squirls features

| Feature | Value |
| --- | --- |
| *Ri wt* donor | 4.10 |
| *ΔRi* canonical donor | 0.00 |
| *ΔRi wt* closest donor | 0.00 |
| Donor offset | -449 |
| max *Ri* cryptic donor window | 9.14 |
| *ΔRi* cryptic donor | 5.04 |
| phyloP | -0.48 |

Cryptic donor

Using cryptic donor site at *15:91,337,139*
would lead to addition
of 3065
bases
to the coding sequence.

Canonical donor site

T
G
A
C
C
G
T
A
C
T
A
G
C
T
A
G
A
G
C
T
C
T
G
A
C
T
G
A
C
T
A
G
C
A
G
T
-3

-2

-1

1

2

3

4

5

6

t
g
a
g
t
a
a
g
c

Predicted cryptic donor site

T
G
A
C
C
G
T
A
C
T
A
G
C
T
A
G
A
G
C
T
C
T
G
A
C
T
G
A
C
T
A
G
C
A
G
T
3063

.

.

.

3066

.

.

.

.

.

3071

.

a
a
t
a
t
a
a
g
t
g

**1:94,526,934 T>C**

***ABCA4***

Squirls score:
**0.285**

The variant overlaps with 3
transcripts:

| Transcript Accession | CDS Change | Variant Effect | Squirls Score |
| --- | --- | --- | --- |
| *ENST00000370225.3* | c.1938-619A>G | CODING\_TRANSCRIPT\_INTRON\_VARIANT | 0.285 |
| *ENST00000472033.1* | n.57+5A>G | SPLICE\_REGION\_VARIANT | 0.013 |
| *ENST00000535735.1* | c.1938-619A>G | CODING\_TRANSCRIPT\_INTRON\_VARIANT | 0.285 |

Squirls features

| Feature | Value |
| --- | --- |
| *Ri wt* donor | 3.39 |
| *ΔRi* canonical donor | 0.00 |
| *ΔRi wt* closest donor | 0.00 |
| Donor offset | -842 |
| max *Ri* cryptic donor window | 9.50 |
| *ΔRi* cryptic donor | 6.11 |
| phyloP | -2.57 |

Cryptic donor

Using cryptic donor site at *1:94,526,930*
would lead to addition
of 1195
bases
to the coding sequence.

Canonical donor site

T
G
A
C
C
G
T
A
C
T
A
G
C
T
A
G
A
G
C
T
C
T
G
A
C
T
G
A
C
T
A
G
C
A
G
T
-3

-2

-1

1

2

3

4

5

6

t
t
c
g
t
g
a
g
t

Predicted cryptic donor site

T
G
A
C
C
G
T
A
C
T
A
G
C
T
A
G
A
G
C
T
C
T
G
A
C
T
G
A
C
T
A
G
C
A
G
T
1193

.

.

.

1196

.

.

.

.

.

1201

.

c
a
g
g
t
g
g
a
t
g

**5:112,115,546 G>A**

***APC***

Squirls score:
**0.283**

The variant overlaps with 6
transcripts:

| Transcript Accession | CDS Change | Variant Effect | Squirls Score |
| --- | --- | --- | --- |
| *ENST00000257430.4* | c.532-941G>A | CODING\_TRANSCRIPT\_INTRON\_VARIANT | 0.283 |
| *ENST00000457016.1* | c.532-941G>A | CODING\_TRANSCRIPT\_INTRON\_VARIANT | 0.283 |
| *ENST00000507379.1* | c.562-941G>A | CODING\_TRANSCRIPT\_INTRON\_VARIANT | 0.283 |
| *ENST00000508376.2* | c.532-941G>A | CODING\_TRANSCRIPT\_INTRON\_VARIANT | 0.283 |
| *ENST00000508624.1* | c.532-941G>A | CODING\_TRANSCRIPT\_INTRON\_VARIANT | 0.283 |
| *ENST00000512211.2* | c.532-941G>A | CODING\_TRANSCRIPT\_INTRON\_VARIANT | 0.283 |

Squirls features

| Feature | Value |
| --- | --- |
| *Ri wt* donor | 9.14 |
| *ΔRi* canonical donor | 0.00 |
| *ΔRi wt* closest donor | 0.00 |
| Donor offset | -1055 |
| max *Ri* cryptic donor window | 10.46 |
| *ΔRi* cryptic donor | 1.31 |
| phyloP | 1.18 |

Cryptic donor

Using cryptic donor site at *5:112,115,548*
would lead to addition
of 4114
bases
to the coding sequence.

Canonical donor site

T
G
A
C
C
G
T
A
C
T
A
G
C
T
A
G
A
G
C
T
C
T
G
A
C
T
G
A
C
T
A
G
C
A
G
T
-3

-2

-1

1

2

3

4

5

6

a
a
t
g
t
a
a
g
t

Predicted cryptic donor site

T
G
A
C
C
G
T
A
C
T
A
G
C
T
A
G
A
G
C
T
C
T
G
A
C
T
G
A
C
T
A
G
C
A
G
T
4112

.

.

.

4115

.

.

.

.

.

4120

.

a
g
g
g
t
g
a
g
a
a

**X:31,279,780 T>C**

***DMD***

Squirls score:
**0.274**

The variant overlaps with 13
transcripts:

| Transcript Accession | CDS Change | Variant Effect | Squirls Score |
| --- | --- | --- | --- |
| *ENST00000343523.2* | c.1845-647A>G | CODING\_TRANSCRIPT\_INTRON\_VARIANT | 0.154 |
| *ENST00000357033.4* | c.9225-647A>G | CODING\_TRANSCRIPT\_INTRON\_VARIANT | 0.154 |
| *ENST00000358062.2* | c.2313-647A>G | CODING\_TRANSCRIPT\_INTRON\_VARIANT | 0.154 |
| *ENST00000359836.1* | c.1845-647A>G | CODING\_TRANSCRIPT\_INTRON\_VARIANT | 0.154 |
| *ENST00000361471.4* | c.21-647A>G | CODING\_TRANSCRIPT\_INTRON\_VARIANT | 0.274 |
| *ENST00000378677.2* | c.9213-647A>G | CODING\_TRANSCRIPT\_INTRON\_VARIANT | 0.154 |
| *ENST00000378680.2* | c.21-647A>G | CODING\_TRANSCRIPT\_INTRON\_VARIANT | 0.274 |
| *ENST00000378702.4* | c.21-647A>G | CODING\_TRANSCRIPT\_INTRON\_VARIANT | 0.274 |
| *ENST00000378707.3* | c.1845-647A>G | CODING\_TRANSCRIPT\_INTRON\_VARIANT | 0.154 |
| *ENST00000378723.3* | c.21-647A>G | CODING\_TRANSCRIPT\_INTRON\_VARIANT | 0.274 |
| *ENST00000469142.1* | n.244-647A>G | NON\_CODING\_TRANSCRIPT\_INTRON\_VARIANT | 0.154 |
| *ENST00000474231.1* | c.1845-647A>G | CODING\_TRANSCRIPT\_INTRON\_VARIANT | 0.154 |
| *ENST00000541735.1* | c.1845-647A>G | CODING\_TRANSCRIPT\_INTRON\_VARIANT | 0.154 |

Squirls features

| Feature | Value |
| --- | --- |
| *Ri wt* donor | 10.24 |
| *ΔRi* canonical donor | 0.00 |
| *ΔRi wt* closest donor | 0.00 |
| Donor offset | -709 |
| max *Ri* cryptic donor window | 9.72 |
| *ΔRi* cryptic donor | -0.52 |
| phyloP | 0.72 |

Cryptic donor

Using cryptic donor site at *X:31,279,781*
would lead to addition
of 5148
bases
to the coding sequence.

Canonical donor site

T
G
A
C
C
G
T
A
C
T
A
G
C
T
A
G
A
G
C
T
C
T
G
A
C
T
G
A
C
T
A
G
C
A
G
T
-3

-2

-1

1

2

3

4

5

6

a
g
g
g
t
a
a
g
t

Predicted cryptic donor site

T
G
A
C
C
G
T
A
C
T
A
G
C
T
A
G
A
G
C
T
C
T
G
A
C
T
G
A
C
T
A
G
C
A
G
T
5146

.

.

.

5149

.

.

.

.

.

5154

.

c
a
a
g
t
a
a
a
t
g

**13:113,770,192 G>A**

***F7***

Squirls score:
**0.253**

The variant overlaps with 5
transcripts:

| Transcript Accession | CDS Change | Variant Effect | Squirls Score |
| --- | --- | --- | --- |
| *ENST00000346342.3* | c.505+78G>A | CODING\_TRANSCRIPT\_INTRON\_VARIANT | 0.040 |
| *ENST00000375581.3* | c.571+78G>A | CODING\_TRANSCRIPT\_INTRON\_VARIANT | 0.040 |
| *ENST00000444337.1* | c.\*313+78G>A | THREE\_PRIME\_UTR\_INTRON\_VARIANT | 0.040 |
| *ENST00000479674.1* | n.698-888G>A | NON\_CODING\_TRANSCRIPT\_INTRON\_VARIANT | 0.253 |
| *ENST00000541084.1* | c.364+78G>A | CODING\_TRANSCRIPT\_INTRON\_VARIANT | 0.040 |

Squirls features

| Feature | Value |
| --- | --- |
| *Ri wt* donor | 4.18 |
| *ΔRi* canonical donor | 0.00 |
| *ΔRi wt* closest donor | 0.00 |
| Donor offset | -958 |
| max *Ri* cryptic donor window | 8.08 |
| *ΔRi* cryptic donor | 3.90 |
| phyloP | 0.95 |

Cryptic donor

Using cryptic donor site at *13:113,770,194*
would lead to addition
of 1920
bases
to the coding sequence.

Canonical donor site

T
G
A
C
C
G
T
A
C
T
A
G
C
T
A
G
A
G
C
T
C
T
G
A
C
T
G
A
C
T
A
G
C
A
G
T
-3

-2

-1

1

2

3

4

5

6

c
g
c
g
t
a
a
g
g

Predicted cryptic donor site

T
G
A
C
C
G
T
A
C
T
A
G
C
T
A
G
A
G
C
T
C
T
G
A
C
T
G
A
C
T
A
G
C
A
G
T
1918

.

.

.

1921

.

.

.

.

.

1926

.

c
g
g
g
t
g
g
g
a
a

**15:48,721,629 T>C**

***FBN1***

Squirls score:
**0.249**

The variant overlaps with 3
transcripts:

| Transcript Accession | CDS Change | Variant Effect | Squirls Score |
| --- | --- | --- | --- |
| *ENST00000316623.5* | c.6872-961A>G | CODING\_TRANSCRIPT\_INTRON\_VARIANT | 0.249 |
| *ENST00000559133.1* | c.2179-961A>G | CODING\_TRANSCRIPT\_INTRON\_VARIANT | 0.249 |
| *ENST00000560720.1* | n.159-961A>G | NON\_CODING\_TRANSCRIPT\_INTRON\_VARIANT | 0.038 |

Squirls features

| Feature | Value |
| --- | --- |
| *ΔRi* canonical acceptor | 0.00 |
| *ΔRi* cryptic acceptor | 6.66 |
| Creates AG in *AGEZ* | No |
| Acceptor offset | -961 |
| Exon length | -1 |
| ESRSeq | -2.30 |
| SMS | -0.84 |
| phyloP | 1.01 |

Cryptic acceptor

Using cryptic acceptor site at *15:48,721,629*
would lead to addition
of 961
bases
to the coding sequence.

Canonical acceptor site

G
C
A
T
G
A
C
T
G
A
C
T
G
A
C
T
G
A
C
T
G
A
C
T
G
A
C
T
G
A
C
T
G
A
C
T
A
G
C
T
A
G
C
T
A
G
C
T
A
G
C
T
A
G
C
T
A
G
C
T
A
G
C
T
A
G
C
T
G
A
C
T
G
A
C
T
G
A
C
T
G
A
C
T
G
A
C
T
G
A
T
C
C
T
G
A
A
T
C
G
T
C
A
G
C
G
A
T
-25
.
.
.
.
-20
.
.
.
.
-15
.
.
.
.
.
-9
-8
-7
-6
-5
-4
-3
-2
-1
1
2
t
t
g
g
t
c
c
t
t
c
a
a
t
a
a
a
a
t
c
a
a
a
c
a
g
a
t

Predicted cryptic acceptor site

G
C
A
T
G
A
C
T
G
A
C
T
G
A
C
T
G
A
C
T
G
A
C
T
G
A
C
T
G
A
C
T
G
A
C
T
A
G
C
T
A
G
C
T
A
G
C
T
A
G
C
T
A
G
C
T
A
G
C
T
A
G
C
T
A
G
C
T
G
A
C
T
G
A
C
T
G
A
C
T
G
A
C
T
G
A
C
T
G
A
T
C
C
T
G
A
A
T
C
G
T
C
A
G
C
G
A
T
-986
.
.
.
.
.
-981
.
.
.
.
.
-976
.
.
.
.
.
-971
.
.
.
.
.
-966
.
.
.
.
.
-961
.
.
t
t
t
g
a
t
c
t
t
t
c
t
a
a
a
t
a
a
t
c
a
t
c
a
g
a
t
g

**1:155,206,890 G>T**

***GBA***

Squirls score:
**0.249**

The variant overlaps with 8
transcripts:

| Transcript Accession | CDS Change | Variant Effect | Squirls Score |
| --- | --- | --- | --- |
| *ENST00000327247.5* | c.999+242C>A | CODING\_TRANSCRIPT\_INTRON\_VARIANT | 0.091 |
| *ENST00000368373.3* | c.999+242C>A | CODING\_TRANSCRIPT\_INTRON\_VARIANT | 0.091 |
| *ENST00000427500.3* | c.852+242C>A | CODING\_TRANSCRIPT\_INTRON\_VARIANT | 0.091 |
| *ENST00000428024.3* | c.738+242C>A | CODING\_TRANSCRIPT\_INTRON\_VARIANT | 0.091 |
| *ENST00000484489.1* | n.340-811C>A | NON\_CODING\_TRANSCRIPT\_INTRON\_VARIANT | 0.249 |
| *ENST00000491081.1* | n.604+242C>A | NON\_CODING\_TRANSCRIPT\_INTRON\_VARIANT | 0.091 |
| *ENST00000497670.1* | n.622+242C>A | NON\_CODING\_TRANSCRIPT\_INTRON\_VARIANT | 0.091 |
| *ENST00000536770.1* | c.660+242C>A | CODING\_TRANSCRIPT\_INTRON\_VARIANT | 0.091 |

Squirls features

| Feature | Value |
| --- | --- |
| *Ri wt* donor | 2.24 |
| *ΔRi* canonical donor | 0.00 |
| *ΔRi wt* closest donor | 0.00 |
| Donor offset | -855 |
| max *Ri* cryptic donor window | 10.53 |
| *ΔRi* cryptic donor | 8.29 |
| phyloP | -0.29 |

Cryptic donor

Using cryptic donor site at *1:155,206,888*
would lead to addition
of 2872
bases
to the coding sequence.

Canonical donor site

T
G
A
C
C
G
T
A
C
T
A
G
C
T
A
G
A
G
C
T
C
T
G
A
C
T
G
A
C
T
A
G
C
A
G
T
-3

-2

-1

1

2

3

4

5

6

t
t
g
g
t
a
c
c
t

Predicted cryptic donor site

T
G
A
C
C
G
T
A
C
T
A
G
C
T
A
G
A
G
C
T
C
T
G
A
C
T
G
A
C
T
A
G
C
A
G
T
2870

.

.

.

2873

.

.

.

.

.

2878

.

g
a
g
g
t
c
a
g
g
a

**13:113,770,688 C>G**

***F7***

Squirls score:
**0.230**

The variant overlaps with 5
transcripts:

| Transcript Accession | CDS Change | Variant Effect | Squirls Score |
| --- | --- | --- | --- |
| *ENST00000346342.3* | c.506-392C>G | CODING\_TRANSCRIPT\_INTRON\_VARIANT | 0.122 |
| *ENST00000375581.3* | c.572-392C>G | CODING\_TRANSCRIPT\_INTRON\_VARIANT | 0.122 |
| *ENST00000444337.1* | c.\*314-392C>G | THREE\_PRIME\_UTR\_INTRON\_VARIANT | 0.122 |
| *ENST00000479674.1* | n.698-392C>G | NON\_CODING\_TRANSCRIPT\_INTRON\_VARIANT | 0.230 |
| *ENST00000541084.1* | c.365-392C>G | CODING\_TRANSCRIPT\_INTRON\_VARIANT | 0.122 |

Squirls features

| Feature | Value |
| --- | --- |
| *Ri wt* donor | 4.18 |
| *ΔRi* canonical donor | 0.00 |
| *ΔRi wt* closest donor | 0.00 |
| Donor offset | -462 |
| max *Ri* cryptic donor window | 7.98 |
| *ΔRi* cryptic donor | 3.80 |
| phyloP | -0.69 |

Cryptic donor

Using cryptic donor site at *13:113,770,688*
would lead to addition
of 2414
bases
to the coding sequence.

Canonical donor site

T
G
A
C
C
G
T
A
C
T
A
G
C
T
A
G
A
G
C
T
C
T
G
A
C
T
G
A
C
T
A
G
C
A
G
T
-3

-2

-1

1

2

3

4

5

6

c
g
c
g
t
a
a
g
g

Predicted cryptic donor site

T
G
A
C
C
G
T
A
C
T
A
G
C
T
A
G
A
G
C
T
C
T
G
A
C
T
G
A
C
T
A
G
C
A
G
T
2412

.

.

.

2415

.

.

.

.

.

2420

.

a
a
a
c
t
a
a
g
c
g

**11:5,247,102 A>C**

***HBB***

Squirls score:
**0.223**

The variant overlaps with 2
transcripts:

| Transcript Accession | CDS Change | Variant Effect | Squirls Score |
| --- | --- | --- | --- |
| *ENST00000335295.4* | c.316-146T>G | CODING\_TRANSCRIPT\_INTRON\_VARIANT | 0.223 |
| *ENST00000475226.1* | n.248-146T>G | NON\_CODING\_TRANSCRIPT\_INTRON\_VARIANT | 0.223 |

Squirls features

| Feature | Value |
| --- | --- |
| *Ri wt* donor | 9.43 |
| *ΔRi* canonical donor | 0.00 |
| *ΔRi wt* closest donor | 0.00 |
| Donor offset | -409 |
| max *Ri* cryptic donor window | 10.44 |
| *ΔRi* cryptic donor | 1.01 |
| phyloP | -0.32 |

Cryptic donor

Using cryptic donor site at *11:5,247,103*
would lead to addition
of 706
bases
to the coding sequence.

Canonical donor site

T
G
A
C
C
G
T
A
C
T
A
G
C
T
A
G
A
G
C
T
C
T
G
A
C
T
G
A
C
T
A
G
C
A
G
T
-3

-2

-1

1

2

3

4

5

6

a
g
g
g
t
g
a
g
t

Predicted cryptic donor site

T
G
A
C
C
G
T
A
C
T
A
G
C
T
A
G
A
G
C
T
C
T
G
A
C
T
G
A
C
T
A
G
C
A
G
T
704

.

.

.

707

.

.

.

.

.

712

.

g
a
t
g
t
a
a
g
a
g

**2:47,635,062 T>G**

***MSH2***

Squirls score:
**0.217**

The variant overlaps with 4
transcripts:

| Transcript Accession | CDS Change | Variant Effect | Squirls Score |
| --- | --- | --- | --- |
| *ENST00000233146.2* | c.212-478T>G | CODING\_TRANSCRIPT\_INTRON\_VARIANT | 0.217 |
| *ENST00000406134.1* | c.212-478T>G | CODING\_TRANSCRIPT\_INTRON\_VARIANT | 0.217 |
| *ENST00000454849.1* | c.14-478T>G | CODING\_TRANSCRIPT\_INTRON\_VARIANT | 0.217 |
| *ENST00000543555.1* | c.14-478T>G | CODING\_TRANSCRIPT\_INTRON\_VARIANT | 0.217 |

Squirls features

| Feature | Value |
| --- | --- |
| *Ri wt* donor | 10.66 |
| *ΔRi* canonical donor | 0.00 |
| *ΔRi wt* closest donor | 0.00 |
| Donor offset | -633 |
| max *Ri* cryptic donor window | 10.53 |
| *ΔRi* cryptic donor | -0.13 |
| phyloP | 0.21 |

Cryptic donor

Using cryptic donor site at *2:47,635,062*
would lead to addition
of 4521
bases
to the coding sequence.

Canonical donor site

T
G
A
C
C
G
T
A
C
T
A
G
C
T
A
G
A
G
C
T
C
T
G
A
C
T
G
A
C
T
A
G
C
A
G
T
-3

-2

-1

1

2

3

4

5

6

c
a
g
g
t
g
a
g
g

Predicted cryptic donor site

T
G
A
C
C
G
T
A
C
T
A
G
C
T
A
G
A
G
C
T
C
T
G
A
C
T
G
A
C
T
A
G
C
A
G
T
4519

.

.

.

4522

.

.

.

.

.

4527

.

g
a
g
t
t
a
a
g
g
g

**16:88,898,676 T>C**

***GALNS***

Squirls score:
**0.215**

The variant overlaps with 6
transcripts:

| Transcript Accession | CDS Change | Variant Effect | Squirls Score |
| --- | --- | --- | --- |
| *ENST00000268695.5* | c.899-167A>G | CODING\_TRANSCRIPT\_INTRON\_VARIANT | 0.215 |
| *ENST00000542788.1* | c.674-167A>G | CODING\_TRANSCRIPT\_INTRON\_VARIANT | 0.215 |
| *ENST00000562593.1* | n.4308-167A>G | NON\_CODING\_TRANSCRIPT\_INTRON\_VARIANT | 0.215 |
| *ENST00000562931.1* | n.487-167A>G | NON\_CODING\_TRANSCRIPT\_INTRON\_VARIANT | 0.215 |
| *ENST00000567525.1* | c.\*370-167A>G | THREE\_PRIME\_UTR\_INTRON\_VARIANT | 0.215 |
| *ENST00000568613.1* | c.\*862-167A>G | THREE\_PRIME\_UTR\_INTRON\_VARIANT | 0.215 |

Squirls features

| Feature | Value |
| --- | --- |
| *Ri wt* donor | 11.87 |
| *ΔRi* canonical donor | 0.00 |
| *ΔRi wt* closest donor | 0.00 |
| Donor offset | -271 |
| max *Ri* cryptic donor window | 10.33 |
| *ΔRi* cryptic donor | -1.54 |
| phyloP | -0.83 |

Cryptic donor

Using cryptic donor site at *16:88,898,676*
would lead to addition
of 2945
bases
to the coding sequence.

Canonical donor site

T
G
A
C
C
G
T
A
C
T
A
G
C
T
A
G
A
G
C
T
C
T
G
A
C
T
G
A
C
T
A
G
C
A
G
T
-3

-2

-1

1

2

3

4

5

6

a
a
g
g
t
g
a
g
t

Predicted cryptic donor site

T
G
A
C
C
G
T
A
C
T
A
G
C
T
A
G
A
G
C
T
C
T
G
A
C
T
G
A
C
T
A
G
C
A
G
T
2943

.

.

.

2946

.

.

.

.

.

2951

.

c
a
g
a
t
g
a
g
c
g

**18:21,132,700 C>T**

***NPC1***

Squirls score:
**0.210**

The variant overlaps with 5
transcripts:

| Transcript Accession | CDS Change | Variant Effect | Squirls Score |
| --- | --- | --- | --- |
| *ENST00000269228.5* | c.1554-1009G>A | CODING\_TRANSCRIPT\_INTRON\_VARIANT | 0.210 |
| *ENST00000412552.2* | c.803+2022G>A | CODING\_TRANSCRIPT\_INTRON\_VARIANT | 0.037 |
| *ENST00000540608.1* | n.1468-1009G>A | NON\_CODING\_TRANSCRIPT\_INTRON\_VARIANT | 0.210 |
| *ENST00000590301.1* | n.229-1009G>A | NON\_CODING\_TRANSCRIPT\_INTRON\_VARIANT | 0.210 |
| *ENST00000591051.1* | c.835+2022G>A | CODING\_TRANSCRIPT\_INTRON\_VARIANT | 0.037 |

Squirls features

| Feature | Value |
| --- | --- |
| *Ri wt* donor | 10.10 |
| *ΔRi* canonical donor | 0.00 |
| *ΔRi wt* closest donor | 0.00 |
| Donor offset | -1110 |
| max *Ri* cryptic donor window | 9.50 |
| *ΔRi* cryptic donor | -0.60 |
| phyloP | 0.12 |

Cryptic donor

Using cryptic donor site at *18:21,132,702*
would lead to addition
of 2024
bases
to the coding sequence.

Canonical donor site

T
G
A
C
C
G
T
A
C
T
A
G
C
T
A
G
A
G
C
T
C
T
G
A
C
T
G
A
C
T
A
G
C
A
G
T
-3

-2

-1

1

2

3

4

5

6

a
c
g
g
t
a
a
g
t

Predicted cryptic donor site

T
G
A
C
C
G
T
A
C
T
A
G
C
T
A
G
A
G
C
T
C
T
G
A
C
T
G
A
C
T
A
G
C
A
G
T
2022

.

.

.

2025

.

.

.

.

.

2030

.

c
g
g
g
t
g
g
g
t
a

**18:21,114,289 G>A**

***NPC1***

Squirls score:
**0.204**

The variant overlaps with 7
transcripts:

| Transcript Accession | CDS Change | Variant Effect | Squirls Score |
| --- | --- | --- | --- |
| *ENST00000269228.5* | c.3591+121C>T | CODING\_TRANSCRIPT\_INTRON\_VARIANT | 0.163 |
| *ENST00000412552.2* | c.2637+121C>T | CODING\_TRANSCRIPT\_INTRON\_VARIANT | 0.163 |
| *ENST00000586150.1* | c.346+121C>T | CODING\_TRANSCRIPT\_INTRON\_VARIANT | 0.163 |
| *ENST00000587163.1* | n.115+121C>T | NON\_CODING\_TRANSCRIPT\_INTRON\_VARIANT | 0.163 |
| *ENST00000588867.1* | n.467C>T | NON\_CODING\_TRANSCRIPT\_EXON\_VARIANT | 0.204 |
| *ENST00000591051.1* | c.2669+121C>T | CODING\_TRANSCRIPT\_INTRON\_VARIANT | 0.163 |
| *ENST00000591107.2* | c.268+121C>T | CODING\_TRANSCRIPT\_INTRON\_VARIANT | 0.163 |

**4:15,989,860 T>C**

***PROM1***

Squirls score:
**0.191**

The variant overlaps with 7
transcripts:

| Transcript Accession | CDS Change | Variant Effect | Squirls Score |
| --- | --- | --- | --- |
| *ENST00000447510.2* | c.2077-521A>G | CODING\_TRANSCRIPT\_INTRON\_VARIANT | 0.191 |
| *ENST00000505450.1* | c.2050-521A>G | CODING\_TRANSCRIPT\_INTRON\_VARIANT | 0.191 |
| *ENST00000508167.1* | c.2050-521A>G | CODING\_TRANSCRIPT\_INTRON\_VARIANT | 0.191 |
| *ENST00000510224.1* | c.2077-521A>G | CODING\_TRANSCRIPT\_INTRON\_VARIANT | 0.191 |
| *ENST00000539194.1* | c.2077-521A>G | CODING\_TRANSCRIPT\_INTRON\_VARIANT | 0.191 |
| *ENST00000540805.1* | c.2077-521A>G | CODING\_TRANSCRIPT\_INTRON\_VARIANT | 0.191 |
| *ENST00000543373.1* | c.2050-521A>G | CODING\_TRANSCRIPT\_INTRON\_VARIANT | 0.191 |

Squirls features

| Feature | Value |
| --- | --- |
| *Ri wt* donor | 5.48 |
| *ΔRi* canonical donor | 0.00 |
| *ΔRi wt* closest donor | 0.00 |
| Donor offset | -575 |
| max *Ri* cryptic donor window | 7.68 |
| *ΔRi* cryptic donor | 2.21 |
| phyloP | -0.33 |

Cryptic donor

Using cryptic donor site at *4:15,989,860*
would lead to addition
of 1495
bases
to the coding sequence.

Canonical donor site

T
G
A
C
C
G
T
A
C
T
A
G
C
T
A
G
A
G
C
T
C
T
G
A
C
T
G
A
C
T
A
G
C
A
G
T
-3

-2

-1

1

2

3

4

5

6

c
t
g
g
t
a
a
c
a

Predicted cryptic donor site

T
G
A
C
C
G
T
A
C
T
A
G
C
T
A
G
A
G
C
T
C
T
G
A
C
T
G
A
C
T
A
G
C
A
G
T
1493

.

.

.

1496

.

.

.

.

.

1501

.

a
g
g
a
t
a
t
g
t
g

**9:21,968,795 A>C**

***CDKN2A***

Squirls score:
**0.189**

The variant overlaps with 12
transcripts:

| Transcript Accession | CDS Change | Variant Effect | Squirls Score |
| --- | --- | --- | --- |
| *ENST00000304494.5* | c.458-554T>G | CODING\_TRANSCRIPT\_INTRON\_VARIANT | 0.008 |
| *ENST00000361570.3* | c.\*102-554T>G | THREE\_PRIME\_UTR\_INTRON\_VARIANT | 0.008 |
| *ENST00000380151.3* | c.\*381-554T>G | THREE\_PRIME\_UTR\_INTRON\_VARIANT | 0.008 |
| *ENST00000404796.2* | c.348-60637A>C | CODING\_TRANSCRIPT\_INTRON\_VARIANT | 0.006 |
| *ENST00000446177.1* | c.458-25T>G | CODING\_TRANSCRIPT\_INTRON\_VARIANT | 0.124 |
| *ENST00000494262.1* | c.305-554T>G | CODING\_TRANSCRIPT\_INTRON\_VARIANT | 0.008 |
| *ENST00000498124.1* | c.458-25T>G | CODING\_TRANSCRIPT\_INTRON\_VARIANT | 0.189 |
| *ENST00000498628.2* | c.305-554T>G | CODING\_TRANSCRIPT\_INTRON\_VARIANT | 0.008 |
| *ENST00000530628.2* | c.\*28-554T>G | THREE\_PRIME\_UTR\_INTRON\_VARIANT | 0.008 |
| *ENST00000578845.2* | c.305-554T>G | CODING\_TRANSCRIPT\_INTRON\_VARIANT | 0.008 |
| *ENST00000579122.1* | c.384-554T>G | CODING\_TRANSCRIPT\_INTRON\_VARIANT | 0.008 |
| *ENST00000579755.1* | c.\*102-554T>G | THREE\_PRIME\_UTR\_INTRON\_VARIANT | 0.008 |

Squirls features

| Feature | Value |
| --- | --- |
| *ΔRi* canonical acceptor | 1.14 |
| *ΔRi* cryptic acceptor | 0.00 |
| Creates AG in *AGEZ* | Yes |
| Acceptor offset | -25 |
| Exon length | 197 |
| ESRSeq | 3.92 |
| SMS | 2.48 |
| phyloP | -2.61 |

Canonical acceptor

Canonical acceptor site

G
C
A
T
G
A
C
T
G
A
C
T
G
A
C
T
G
A
C
T
G
A
C
T
G
A
C
T
G
A
C
T
G
A
C
T
A
G
C
T
A
G
C
T
A
G
C
T
A
G
C
T
A
G
C
T
A
G
C
T
A
G
C
T
A
G
C
T
G
A
C
T
G
A
C
T
G
A
C
T
G
A
C
T
G
A
C
T
G
A
T
C
C
T
G
A
A
T
C
G
T
C
A
G
C
G
A
T
-25
.
.
.
.
-20
.
.
.
.
-15
.
.
.
.
.
-9
-8
-7
-6
-5
-4
-3
-2
-1
1
2
t
a
a
a
t
g
a
g
c
a
t
c
c
a
t
t
t
t
g
t
t
t
c
a
g
a
a
g

*ΔRi* score distribution

-10


-5


0


5


10

ΔRi
: 1.14

**11:47,364,834 T>C**

***MYBPC3***

Squirls score:
**0.174**

The variant overlaps with 4
transcripts:

| Transcript Accession | CDS Change | Variant Effect | Squirls Score |
| --- | --- | --- | --- |
| *ENST00000256993.4* | c.1224-138A>G | CODING\_TRANSCRIPT\_INTRON\_VARIANT | 0.008 |
| *ENST00000399249.2* | c.1224-21A>G | CODING\_TRANSCRIPT\_INTRON\_VARIANT | 0.174 |
| *ENST00000544791.1* | c.1224-21A>G | CODING\_TRANSCRIPT\_INTRON\_VARIANT | 0.174 |
| *ENST00000545968.1* | c.1224-21A>G | CODING\_TRANSCRIPT\_INTRON\_VARIANT | 0.174 |

Squirls features

| Feature | Value |
| --- | --- |
| *ΔRi* canonical acceptor | 0.51 |
| *ΔRi* cryptic acceptor | 0.00 |
| Creates AG in *AGEZ* | Yes |
| Acceptor offset | -21 |
| Exon length | 3 |
| ESRSeq | -1.73 |
| SMS | -0.08 |
| phyloP | -0.06 |

Canonical acceptor

Canonical acceptor site

G
C
A
T
G
A
C
T
G
A
C
T
G
A
C
T
G
A
C
T
G
A
C
T
G
A
C
T
G
A
C
T
G
A
C
T
A
G
C
T
A
G
C
T
A
G
C
T
A
G
C
T
A
G
C
T
A
G
C
T
A
G
C
T
A
G
C
T
G
A
C
T
G
A
C
T
G
A
C
T
G
A
C
T
G
A
C
T
G
A
T
C
C
T
G
A
A
T
C
G
T
C
A
G
C
G
A
T
-25
.
.
.
.
-20
.
.
.
.
-15
.
.
.
.
.
-9
-8
-7
-6
-5
-4
-3
-2
-1
1
2
t
t
c
a
a
c
g
g
c
c
c
c
t
t
c
t
g
t
t
c
t
a
c
a
g
c
a
g

*ΔRi* score distribution

-10


-5


0


5


10

ΔRi
: 0.51

**X:32,366,456 T>C**

***DMD***

Squirls score:
**0.163**

The variant overlaps with 4
transcripts:

| Transcript Accession | CDS Change | Variant Effect | Squirls Score |
| --- | --- | --- | --- |
| *ENST00000357033.4* | c.5448+67A>G | CODING\_TRANSCRIPT\_INTRON\_VARIANT | 0.163 |
| *ENST00000378677.2* | c.5436+67A>G | CODING\_TRANSCRIPT\_INTRON\_VARIANT | 0.163 |
| *ENST00000488902.1* | n.336-131276A>G | NON\_CODING\_TRANSCRIPT\_INTRON\_VARIANT | 0.150 |
| *ENST00000493412.1* | c.105+67A>G | CODING\_TRANSCRIPT\_INTRON\_VARIANT | 0.163 |

Squirls features

| Feature | Value |
| --- | --- |
| *Ri wt* donor | 7.41 |
| *ΔRi* canonical donor | 0.00 |
| *ΔRi wt* closest donor | 0.00 |
| Donor offset | 67 |
| max *Ri* cryptic donor window | 11.26 |
| *ΔRi* cryptic donor | 3.85 |
| phyloP | 1.73 |

Cryptic donor

Using cryptic donor site at *X:32,366,456*
would lead to addition
of 67
bases
to the coding sequence.

Canonical donor site

T
G
A
C
C
G
T
A
C
T
A
G
C
T
A
G
A
G
C
T
C
T
G
A
C
T
G
A
C
T
A
G
C
A
G
T
-3

-2

-1

1

2

3

4

5

6

a
t
g
g
t
a
a
a
t

Predicted cryptic donor site

T
G
A
C
C
G
T
A
C
T
A
G
C
T
A
G
A
G
C
T
C
T
G
A
C
T
G
A
C
T
A
G
C
A
G
T
65

.

.

.

68

.

.

.

.

.

73

.

t
a
g
a
t
a
a
g
t
g

**1:161,284,289 A>G**

***SDHC***

Squirls score:
**0.162**

The variant overlaps with 7
transcripts:

| Transcript Accession | CDS Change | Variant Effect | Squirls Score |
| --- | --- | --- | --- |
| *ENST00000342751.4* | c.20+74A>G | CODING\_TRANSCRIPT\_INTRON\_VARIANT | 0.010 |
| *ENST00000367975.2* | c.20+74A>G | CODING\_TRANSCRIPT\_INTRON\_VARIANT | 0.010 |
| *ENST00000392169.2* | c.20+74A>G | CODING\_TRANSCRIPT\_INTRON\_VARIANT | 0.010 |
| *ENST00000432287.2* | c.20+74A>G | CODING\_TRANSCRIPT\_INTRON\_VARIANT | 0.010 |
| *ENST00000504963.1* | c.20+74A>G | CODING\_TRANSCRIPT\_INTRON\_VARIANT | 0.010 |
| *ENST00000513009.1* | c.20+74A>G | CODING\_TRANSCRIPT\_INTRON\_VARIANT | 0.010 |
| *ENST00000515731.1* | n.119A>G | NON\_CODING\_TRANSCRIPT\_EXON\_VARIANT | 0.162 |

Squirls features

| Feature | Value |
| --- | --- |
| *Ri wt* donor | 4.44 |
| *ΔRi* canonical donor | 0.00 |
| *ΔRi wt* closest donor | -5.20 |
| Donor offset | -376 |
| max *Ri* cryptic donor window | 6.90 |
| *ΔRi* cryptic donor | 2.46 |
| phyloP | 0.77 |

Cryptic donor

Using cryptic donor site at *1:161,284,289*
would lead to removal
of 376
bases
from the coding sequence.

Canonical donor site

T
G
A
C
C
G
T
A
C
T
A
G
C
T
A
G
A
G
C
T
C
T
G
A
C
T
G
A
C
T
A
G
C
A
G
T
-3

-2

-1

1

2

3

4

5

6

a
c
c
g
t
g
a
g
t

Predicted cryptic donor site

T
G
A
C
C
G
T
A
C
T
A
G
C
T
A
G
A
G
C
T
C
T
G
A
C
T
G
A
C
T
A
G
C
A
G
T
-379

.

.

.

-376

.

.

.

.

.

-371

.

c
t
g
a
t
a
a
c
t
g

**9:135,173,806 T>C**

***SETX***

Squirls score:
**0.160**

The variant overlaps with 4
transcripts:

| Transcript Accession | CDS Change | Variant Effect | Squirls Score |
| --- | --- | --- | --- |
| *ENST00000224140.5* | c.5549-107A>G | CODING\_TRANSCRIPT\_INTRON\_VARIANT | 0.160 |
| *ENST00000372169.2* | c.5549-107A>G | CODING\_TRANSCRIPT\_INTRON\_VARIANT | 0.160 |
| *ENST00000393220.1* | c.5549-107A>G | CODING\_TRANSCRIPT\_INTRON\_VARIANT | 0.160 |
| *ENST00000436441.1* | c.275-107A>G | CODING\_TRANSCRIPT\_INTRON\_VARIANT | 0.160 |

Squirls features

| Feature | Value |
| --- | --- |
| *Ri wt* donor | 5.61 |
| *ΔRi* canonical donor | 0.00 |
| *ΔRi wt* closest donor | 0.00 |
| Donor offset | -340 |
| max *Ri* cryptic donor window | 7.71 |
| *ΔRi* cryptic donor | 2.10 |
| phyloP | -0.19 |

Cryptic donor

Using cryptic donor site at *9:135,173,802*
would lead to addition
of 2207
bases
to the coding sequence.

Canonical donor site

T
G
A
C
C
G
T
A
C
T
A
G
C
T
A
G
A
G
C
T
C
T
G
A
C
T
G
A
C
T
A
G
C
A
G
T
-3

-2

-1

1

2

3

4

5

6

t
c
a
g
t
a
a
g
t

Predicted cryptic donor site

T
G
A
C
C
G
T
A
C
T
A
G
C
T
A
G
A
G
C
T
C
T
G
A
C
T
G
A
C
T
A
G
C
A
G
T
2205

.

.

.

2208

.

.

.

.

.

2213

.

t
a
t
g
t
a
a
a
t
g

**8:42,328,683 C>T**

***SLC20A2***

Squirls score:
**0.157**

The variant overlaps with 4
transcripts:

| Transcript Accession | CDS Change | Variant Effect | Squirls Score |
| --- | --- | --- | --- |
| *ENST00000342228.3* | c.289+937G>A | CODING\_TRANSCRIPT\_INTRON\_VARIANT | 0.049 |
| *ENST00000520179.1* | c.289+937G>A | CODING\_TRANSCRIPT\_INTRON\_VARIANT | 0.049 |
| *ENST00000520262.1* | c.289+937G>A | CODING\_TRANSCRIPT\_INTRON\_VARIANT | 0.049 |
| *ENST00000524211.1* | c.\*8G>A | THREE\_PRIME\_UTR\_EXON\_VARIANT | 0.157 |

**X:32,535,101 C>A**

***DMD***

Squirls score:
**0.153**

The variant overlaps with 6
transcripts:

| Transcript Accession | CDS Change | Variant Effect | Squirls Score |
| --- | --- | --- | --- |
| *ENST00000288447.4* | c.\*973G>T | THREE\_PRIME\_UTR\_EXON\_VARIANT | 0.038 |
| *ENST00000357033.4* | c.2292+1024G>T | CODING\_TRANSCRIPT\_INTRON\_VARIANT | 0.153 |
| *ENST00000378677.2* | c.2280+1024G>T | CODING\_TRANSCRIPT\_INTRON\_VARIANT | 0.153 |
| *ENST00000420596.1* | c.94-151785G>T | CODING\_TRANSCRIPT\_INTRON\_VARIANT | 0.058 |
| *ENST00000448370.1* | c.94-152274G>T | CODING\_TRANSCRIPT\_INTRON\_VARIANT | 0.060 |
| *ENST00000488902.1* | n.336-299921G>T | NON\_CODING\_TRANSCRIPT\_INTRON\_VARIANT | 0.058 |

Squirls features

| Feature | Value |
| --- | --- |
| *Ri wt* donor | 6.66 |
| *ΔRi* canonical donor | 0.00 |
| *ΔRi wt* closest donor | 0.00 |
| Donor offset | 1024 |
| max *Ri* cryptic donor window | 11.27 |
| *ΔRi* cryptic donor | 4.61 |
| phyloP | -1.78 |

Cryptic donor

Using cryptic donor site at *X:32,535,100*
would lead to addition
of 1023
bases
to the coding sequence.

Canonical donor site

T
G
A
C
C
G
T
A
C
T
A
G
C
T
A
G
A
G
C
T
C
T
G
A
C
T
G
A
C
T
A
G
C
A
G
T
-3

-2

-1

1

2

3

4

5

6

a
a
t
g
t
a
g
g
t

Predicted cryptic donor site

T
G
A
C
C
G
T
A
C
T
A
G
C
T
A
G
A
G
C
T
C
T
G
A
C
T
G
A
C
T
A
G
C
A
G
T
1021

.

.

.

1024

.

.

.

.

.

1029

.

a
a
g
g
g
a
a
g
a
t

**13:49,046,098 A>G**

***RB1***

Squirls score:
**0.151**

The variant overlaps with 1
transcript:

| Transcript Accession | CDS Change | Variant Effect | Squirls Score |
| --- | --- | --- | --- |
| *ENST00000267163.4* | c.2490-1398A>G | CODING\_TRANSCRIPT\_INTRON\_VARIANT | 0.151 |

Squirls features

| Feature | Value |
| --- | --- |
| *Ri wt* donor | 9.32 |
| *ΔRi* canonical donor | 0.00 |
| *ΔRi wt* closest donor | 0.00 |
| Donor offset | -1429 |
| max *Ri* cryptic donor window | 9.67 |
| *ΔRi* cryptic donor | 0.36 |
| phyloP | 1.11 |

Cryptic donor

Using cryptic donor site at *13:49,046,098*
would lead to addition
of 6594
bases
to the coding sequence.

Canonical donor site

T
G
A
C
C
G
T
A
C
T
A
G
C
T
A
G
A
G
C
T
C
T
G
A
C
T
G
A
C
T
A
G
C
A
G
T
-3

-2

-1

1

2

3

4

5

6

a
a
g
g
t
g
t
g
t

Predicted cryptic donor site

T
G
A
C
C
G
T
A
C
T
A
G
C
T
A
G
A
G
C
T
C
T
G
A
C
T
G
A
C
T
A
G
C
A
G
T
6592

.

.

.

6595

.

.

.

.

.

6600

.

a
t
g
a
t
g
a
g
t
g

**22:30,073,952 T>A**

***NF2***

Squirls score:
**0.148**

The variant overlaps with 12
transcripts:

| Transcript Accession | CDS Change | Variant Effect | Squirls Score |
| --- | --- | --- | --- |
| *ENST00000334961.7* | c.1198-233T>A | CODING\_TRANSCRIPT\_INTRON\_VARIANT | 0.148 |
| *ENST00000338641.4* | c.1447-233T>A | CODING\_TRANSCRIPT\_INTRON\_VARIANT | 0.148 |
| *ENST00000347330.5* | c.629-233T>A | CODING\_TRANSCRIPT\_INTRON\_VARIANT | 0.148 |
| *ENST00000353887.4* | c.1198-233T>A | CODING\_TRANSCRIPT\_INTRON\_VARIANT | 0.148 |
| *ENST00000361166.4* | c.1447-233T>A | CODING\_TRANSCRIPT\_INTRON\_VARIANT | 0.148 |
| *ENST00000361452.4* | c.1324-233T>A | CODING\_TRANSCRIPT\_INTRON\_VARIANT | 0.148 |
| *ENST00000361676.4* | c.1321-233T>A | CODING\_TRANSCRIPT\_INTRON\_VARIANT | 0.148 |
| *ENST00000397789.3* | c.1447-233T>A | CODING\_TRANSCRIPT\_INTRON\_VARIANT | 0.148 |
| *ENST00000403435.1* | c.1360-233T>A | CODING\_TRANSCRIPT\_INTRON\_VARIANT | 0.148 |
| *ENST00000403999.3* | c.1447-233T>A | CODING\_TRANSCRIPT\_INTRON\_VARIANT | 0.148 |
| *ENST00000413209.2* | c.448-16789T>A | CODING\_TRANSCRIPT\_INTRON\_VARIANT | 0.046 |
| *ENST00000432151.1* | c.629-233T>A | CODING\_TRANSCRIPT\_INTRON\_VARIANT | 0.148 |

Squirls features

| Feature | Value |
| --- | --- |
| *Ri wt* donor | 7.74 |
| *ΔRi* canonical donor | 0.00 |
| *ΔRi wt* closest donor | 0.00 |
| Donor offset | -361 |
| max *Ri* cryptic donor window | 8.68 |
| *ΔRi* cryptic donor | 0.94 |
| phyloP | -0.53 |

Cryptic donor

Using cryptic donor site at *22:30,073,950*
would lead to addition
of 3020
bases
to the coding sequence.

Canonical donor site

T
G
A
C
C
G
T
A
C
T
A
G
C
T
A
G
A
G
C
T
C
T
G
A
C
T
G
A
C
T
A
G
C
A
G
T
-3

-2

-1

1

2

3

4

5

6

c
c
g
g
t
g
a
g
c

Predicted cryptic donor site

T
G
A
C
C
G
T
A
C
T
A
G
C
T
A
G
A
G
C
T
C
T
G
A
C
T
G
A
C
T
A
G
C
A
G
T
3018

.

.

.

3021

.

.

.

.

.

3026

.

a
c
g
g
t
t
a
g
a
a

**3:150,660,197 A>C**

***CLRN1***

Squirls score:
**0.146**

The variant overlaps with 6
transcripts:

| Transcript Accession | CDS Change | Variant Effect | Squirls Score |
| --- | --- | --- | --- |
| *ENST00000295911.2* | c.26-649T>G | CODING\_TRANSCRIPT\_INTRON\_VARIANT | 0.098 |
| *ENST00000327047.1* | c.254-649T>G | CODING\_TRANSCRIPT\_INTRON\_VARIANT | 0.146 |
| *ENST00000328863.4* | c.254-649T>G | CODING\_TRANSCRIPT\_INTRON\_VARIANT | 0.146 |
| *ENST00000468836.1* | c.26-649T>G | CODING\_TRANSCRIPT\_INTRON\_VARIANT | 0.098 |
| *ENST00000472224.1* | n.260-649T>G | NON\_CODING\_TRANSCRIPT\_INTRON\_VARIANT | 0.146 |
| *ENST00000485607.1* | c.-84+144T>G | FIVE\_PRIME\_UTR\_INTRON\_VARIANT | 0.084 |

Squirls features

| Feature | Value |
| --- | --- |
| *Ri wt* donor | 9.26 |
| *ΔRi* canonical donor | 0.00 |
| *ΔRi wt* closest donor | 0.00 |
| Donor offset | -829 |
| max *Ri* cryptic donor window | 8.88 |
| *ΔRi* cryptic donor | -0.38 |
| phyloP | 0.61 |

Cryptic donor

Using cryptic donor site at *3:150,660,198*
would lead to addition
of 30047
bases
to the coding sequence.

Canonical donor site

T
G
A
C
C
G
T
A
C
T
A
G
C
T
A
G
A
G
C
T
C
T
G
A
C
T
G
A
C
T
A
G
C
A
G
T
-3

-2

-1

1

2

3

4

5

6

c
a
t
g
t
a
a
g
t

Predicted cryptic donor site

T
G
A
C
C
G
T
A
C
T
A
G
C
T
A
G
A
G
C
T
C
T
G
A
C
T
G
A
C
T
A
G
C
A
G
T
30045

.

.

.

30048

.

.

.

.

.

30053

.

a
a
t
g
t
a
g
g
g
g

**2:71,889,030 G>T**

***DYSF***

Squirls score:
**0.145**

The variant overlaps with 12
transcripts:

| Transcript Accession | CDS Change | Variant Effect | Squirls Score |
| --- | --- | --- | --- |
| *ENST00000258104.3* | c.4886+1249G>T | CODING\_TRANSCRIPT\_INTRON\_VARIANT | 0.145 |
| *ENST00000394120.2* | c.4889+1249G>T | CODING\_TRANSCRIPT\_INTRON\_VARIANT | 0.145 |
| *ENST00000409366.1* | c.4952+1249G>T | CODING\_TRANSCRIPT\_INTRON\_VARIANT | 0.145 |
| *ENST00000409582.3* | c.5000+1249G>T | CODING\_TRANSCRIPT\_INTRON\_VARIANT | 0.145 |
| *ENST00000409651.1* | c.4982+1249G>T | CODING\_TRANSCRIPT\_INTRON\_VARIANT | 0.145 |
| *ENST00000409744.1* | c.4910+1249G>T | CODING\_TRANSCRIPT\_INTRON\_VARIANT | 0.145 |
| *ENST00000409762.1* | c.4937+1249G>T | CODING\_TRANSCRIPT\_INTRON\_VARIANT | 0.145 |
| *ENST00000410020.3* | c.5003+1249G>T | CODING\_TRANSCRIPT\_INTRON\_VARIANT | 0.145 |
| *ENST00000410041.1* | c.4940+1249G>T | CODING\_TRANSCRIPT\_INTRON\_VARIANT | 0.145 |
| *ENST00000413539.2* | c.4979+1249G>T | CODING\_TRANSCRIPT\_INTRON\_VARIANT | 0.145 |
| *ENST00000429174.2* | c.4949+1249G>T | CODING\_TRANSCRIPT\_INTRON\_VARIANT | 0.145 |
| *ENST00000479049.2* | n.1771+1249G>T | NON\_CODING\_TRANSCRIPT\_INTRON\_VARIANT | 0.145 |

Squirls features

| Feature | Value |
| --- | --- |
| *Ri wt* donor | 6.55 |
| *ΔRi* canonical donor | 0.00 |
| *ΔRi wt* closest donor | 0.00 |
| Donor offset | 1249 |
| max *Ri* cryptic donor window | 11.85 |
| *ΔRi* cryptic donor | 5.30 |
| phyloP | 0.16 |

Cryptic donor

Using cryptic donor site at *2:71,889,029*
would lead to addition
of 1248
bases
to the coding sequence.

Canonical donor site

T
G
A
C
C
G
T
A
C
T
A
G
C
T
A
G
A
G
C
T
C
T
G
A
C
T
G
A
C
T
A
G
C
A
G
T
-3

-2

-1

1

2

3

4

5

6

a
a
a
g
t
a
a
a
t

Predicted cryptic donor site

T
G
A
C
C
G
T
A
C
T
A
G
C
T
A
G
A
G
C
T
C
T
G
A
C
T
G
A
C
T
A
G
C
A
G
T
1246

.

.

.

1249

.

.

.

.

.

1254

.

g
a
g
g
g
a
a
g
t
t

**16:56,917,770 C>T**

***SLC12A3***

Squirls score:
**0.136**

The variant overlaps with 4
transcripts:

| Transcript Accession | CDS Change | Variant Effect | Squirls Score |
| --- | --- | --- | --- |
| *ENST00000262502.5* | c.1667-191C>T | CODING\_TRANSCRIPT\_INTRON\_VARIANT | 0.136 |
| *ENST00000438926.2* | c.1670-191C>T | CODING\_TRANSCRIPT\_INTRON\_VARIANT | 0.136 |
| *ENST00000563236.1* | c.1670-191C>T | CODING\_TRANSCRIPT\_INTRON\_VARIANT | 0.136 |
| *ENST00000566786.1* | c.1667-191C>T | CODING\_TRANSCRIPT\_INTRON\_VARIANT | 0.136 |

Squirls features

| Feature | Value |
| --- | --- |
| *Ri wt* donor | 8.95 |
| *ΔRi* canonical donor | 0.00 |
| *ΔRi wt* closest donor | 0.00 |
| Donor offset | -347 |
| max *Ri* cryptic donor window | 9.43 |
| *ΔRi* cryptic donor | 0.48 |
| phyloP | -1.57 |

Cryptic donor

Using cryptic donor site at *16:56,917,769*
would lead to addition
of 1360
bases
to the coding sequence.

Canonical donor site

T
G
A
C
C
G
T
A
C
T
A
G
C
T
A
G
A
G
C
T
C
T
G
A
C
T
G
A
C
T
A
G
C
A
G
T
-3

-2

-1

1

2

3

4

5

6

c
t
g
g
t
a
a
g
c

Predicted cryptic donor site

T
G
A
C
C
G
T
A
C
T
A
G
C
T
A
G
A
G
C
T
C
T
G
A
C
T
G
A
C
T
A
G
C
A
G
T
1358

.

.

.

1361

.

.

.

.

.

1366

.

c
a
g
g
c
g
t
g
t
t

**1:100,672,742 T>C**

***DBT***

Squirls score:
**0.134**

The variant overlaps with 1
transcript:

| Transcript Accession | CDS Change | Variant Effect | Squirls Score |
| --- | --- | --- | --- |
| *ENST00000370132.4* | c.1018-550A>G | CODING\_TRANSCRIPT\_INTRON\_VARIANT | 0.134 |

Squirls features

| Feature | Value |
| --- | --- |
| *Ri wt* donor | 4.37 |
| *ΔRi* canonical donor | 0.00 |
| *ΔRi wt* closest donor | 0.00 |
| Donor offset | -742 |
| max *Ri* cryptic donor window | 6.96 |
| *ΔRi* cryptic donor | 2.58 |
| phyloP | -0.14 |

Cryptic donor

Using cryptic donor site at *1:100,672,742*
would lead to addition
of 3508
bases
to the coding sequence.

Canonical donor site

T
G
A
C
C
G
T
A
C
T
A
G
C
T
A
G
A
G
C
T
C
T
G
A
C
T
G
A
C
T
A
G
C
A
G
T
-3

-2

-1

1

2

3

4

5

6

a
a
g
g
t
t
g
g
c

Predicted cryptic donor site

T
G
A
C
C
G
T
A
C
T
A
G
C
T
A
G
A
G
C
T
C
T
G
A
C
T
G
A
C
T
A
G
C
A
G
T
3506

.

.

.

3509

.

.

.

.

.

3514

.

c
a
g
a
t
a
t
t
t
g

**X:148,568,762 T>C**

***IDS***

Squirls score:
**0.129**

The variant overlaps with 8
transcripts:

| Transcript Accession | CDS Change | Variant Effect | Squirls Score |
| --- | --- | --- | --- |
| *ENST00000340855.6* | c.1007-133A>G | CODING\_TRANSCRIPT\_INTRON\_VARIANT | 0.061 |
| *ENST00000370441.4* | c.\*57A>G | THREE\_PRIME\_UTR\_EXON\_VARIANT | 0.035 |
| *ENST00000422081.2* | c.374-133A>G | CODING\_TRANSCRIPT\_INTRON\_VARIANT | 0.061 |
| *ENST00000441880.1* | n.114-133A>G | NON\_CODING\_TRANSCRIPT\_INTRON\_VARIANT | 0.129 |
| *ENST00000466323.1* | c.\*280A>G | THREE\_PRIME\_UTR\_EXON\_VARIANT | 0.035 |
| *ENST00000490775.1* | n.874A>G | NON\_CODING\_TRANSCRIPT\_EXON\_VARIANT | 0.031 |
| *ENST00000537071.1* | c.-12+3083A>G | FIVE\_PRIME\_UTR\_INTRON\_VARIANT | 0.016 |
| *ENST00000541269.1* | c.374-133A>G | CODING\_TRANSCRIPT\_INTRON\_VARIANT | 0.061 |

Squirls features

| Feature | Value |
| --- | --- |
| *ΔRi* canonical acceptor | 0.00 |
| *ΔRi* cryptic acceptor | 3.88 |
| Creates AG in *AGEZ* | No |
| Acceptor offset | -133 |
| Exon length | -1 |
| ESRSeq | -0.59 |
| SMS | -0.41 |
| phyloP | 0.27 |

Cryptic acceptor

Using cryptic acceptor site at *X:148,568,777*
would lead to addition
of 118
bases
to the coding sequence.

Canonical acceptor site

G
C
A
T
G
A
C
T
G
A
C
T
G
A
C
T
G
A
C
T
G
A
C
T
G
A
C
T
G
A
C
T
G
A
C
T
A
G
C
T
A
G
C
T
A
G
C
T
A
G
C
T
A
G
C
T
A
G
C
T
A
G
C
T
A
G
C
T
G
A
C
T
G
A
C
T
G
A
C
T
G
A
C
T
G
A
C
T
G
A
T
C
C
T
G
A
A
T
C
G
T
C
A
G
C
G
A
T
-25
.
.
.
.
-20
.
.
.
.
-15
.
.
.
.
.
-9
-8
-7
-6
-5
-4
-3
-2
-1
1
2
t
c
a
t
t
c
t
g
t
g
c
t
t
t
t
t
a
t
g
a
a
c
t
a
g
g
g

Predicted cryptic acceptor site

G
C
A
T
G
A
C
T
G
A
C
T
G
A
C
T
G
A
C
T
G
A
C
T
G
A
C
T
G
A
C
T
G
A
C
T
A
G
C
T
A
G
C
T
A
G
C
T
A
G
C
T
A
G
C
T
A
G
C
T
A
G
C
T
A
G
C
T
G
A
C
T
G
A
C
T
G
A
C
T
G
A
C
T
G
A
C
T
G
A
T
C
C
T
G
A
A
T
C
G
T
C
A
G
C
G
A
T
-143
.
.
.
.
.
-138
.
.
.
.
.
-133
.
.
.
.
.
-128
.
.
.
.
.
-123
.
.
.
.
.
-118
.
.
t
t
c
c
a
a
g
t
g
a
a
t
a
t
t
t
t
t
c
t
t
c
t
a
g
g
t
g

**X:13,768,358 A>G**

***OFD1***

Squirls score:
**0.127**

The variant overlaps with 5
transcripts:

| Transcript Accession | CDS Change | Variant Effect | Squirls Score |
| --- | --- | --- | --- |
| *ENST00000340096.6* | c.935+706A>G | CODING\_TRANSCRIPT\_INTRON\_VARIANT | 0.127 |
| *ENST00000380550.3* | c.935+706A>G | CODING\_TRANSCRIPT\_INTRON\_VARIANT | 0.127 |
| *ENST00000380567.1* | c.515+706A>G | CODING\_TRANSCRIPT\_INTRON\_VARIANT | 0.127 |
| *ENST00000398395.3* | c.935+706A>G | CODING\_TRANSCRIPT\_INTRON\_VARIANT | 0.127 |
| *ENST00000490265.1* | n.1246+706A>G | NON\_CODING\_TRANSCRIPT\_INTRON\_VARIANT | 0.127 |

Squirls features

| Feature | Value |
| --- | --- |
| *Ri wt* donor | 6.94 |
| *ΔRi* canonical donor | 0.00 |
| *ΔRi wt* closest donor | 0.00 |
| Donor offset | 706 |
| max *Ri* cryptic donor window | 12.69 |
| *ΔRi* cryptic donor | 5.74 |
| phyloP | 0.18 |

Cryptic donor

Using cryptic donor site at *X:13,768,354*
would lead to addition
of 702
bases
to the coding sequence.

Canonical donor site

T
G
A
C
C
G
T
A
C
T
A
G
C
T
A
G
A
G
C
T
C
T
G
A
C
T
G
A
C
T
A
G
C
A
G
T
-3

-2

-1

1

2

3

4

5

6

a
t
t
g
t
a
a
g
t

Predicted cryptic donor site

T
G
A
C
C
G
T
A
C
T
A
G
C
T
A
G
A
G
C
T
C
T
G
A
C
T
G
A
C
T
A
G
C
A
G
T
700

.

.

.

703

.

.

.

.

.

708

.

a
a
g
g
t
a
a
a
t
g

**15:42,697,047 G>C**

***CAPN3***

Squirls score:
**0.124**

The variant overlaps with 13
transcripts:

| Transcript Accession | CDS Change | Variant Effect | Squirls Score |
| --- | --- | --- | --- |
| *ENST00000318023.7* | c.1782+1072G>C | CODING\_TRANSCRIPT\_INTRON\_VARIANT | 0.010 |
| *ENST00000337571.4* | c.-188G>C | FIVE\_PRIME\_UTR\_EXON\_VARIANT | 0.113 |
| *ENST00000349748.3* | c.1638+1072G>C | CODING\_TRANSCRIPT\_INTRON\_VARIANT | 0.010 |
| *ENST00000356316.3* | c.1521+1072G>C | CODING\_TRANSCRIPT\_INTRON\_VARIANT | 0.010 |
| *ENST00000357568.3* | c.1782+1072G>C | CODING\_TRANSCRIPT\_INTRON\_VARIANT | 0.010 |
| *ENST00000397163.3* | c.1782+1072G>C | CODING\_TRANSCRIPT\_INTRON\_VARIANT | 0.009 |
| *ENST00000397200.4* | c.246+1072G>C | CODING\_TRANSCRIPT\_INTRON\_VARIANT | 0.009 |
| *ENST00000397204.4* | c.-206G>C | FIVE\_PRIME\_UTR\_EXON\_VARIANT | 0.124 |
| *ENST00000561817.1* | c.-188G>C | FIVE\_PRIME\_UTR\_EXON\_VARIANT | 0.113 |
| *ENST00000565173.1* | n.23G>C | NON\_CODING\_TRANSCRIPT\_EXON\_VARIANT | 0.117 |
| *ENST00000567071.1* | c.241+1072G>C | CODING\_TRANSCRIPT\_INTRON\_VARIANT | 0.009 |
| *ENST00000569136.1* | c.-320G>C | FIVE\_PRIME\_UTR\_EXON\_VARIANT | 0.124 |
| *ENST00000569827.1* | c.246+1072G>C | CODING\_TRANSCRIPT\_INTRON\_VARIANT | 0.010 |

Squirls features

| Feature | Value |
| --- | --- |
| *Ri wt* donor | 7.14 |
| *ΔRi* canonical donor | 0.00 |
| *ΔRi wt* closest donor | -3.07 |
| Donor offset | -107 |
| max *Ri* cryptic donor window | 8.12 |
| *ΔRi* cryptic donor | 0.98 |
| phyloP | 0.31 |

Cryptic donor

Using cryptic donor site at *15:42,697,050*
would lead to removal
of 104
bases
from the coding sequence.

Canonical donor site

T
G
A
C
C
G
T
A
C
T
A
G
C
T
A
G
A
G
C
T
C
T
G
A
C
T
G
A
C
T
A
G
C
A
G
T
-3

-2

-1

1

2

3

4

5

6

c
a
c
g
t
g
a
g
t

Predicted cryptic donor site

T
G
A
C
C
G
T
A
C
T
A
G
C
T
A
G
A
G
C
T
C
T
G
A
C
T
G
A
C
T
A
G
C
A
G
T
-107

.

.

.

-104

.

.

.

.

.

-99

.

g
t
g
g
t
a
g
g
t
c

**5:78,180,826 T>C**

***ARSB***

Squirls score:
**0.124**

The variant overlaps with 4
transcripts:

| Transcript Accession | CDS Change | Variant Effect | Squirls Score |
| --- | --- | --- | --- |
| *ENST00000264914.4* | c.1142+581A>G | CODING\_TRANSCRIPT\_INTRON\_VARIANT | 0.124 |
| *ENST00000396151.3* | c.1142+581A>G | CODING\_TRANSCRIPT\_INTRON\_VARIANT | 0.124 |
| *ENST00000521800.1* | n.373A>G | NON\_CODING\_TRANSCRIPT\_EXON\_VARIANT | 0.013 |
| *ENST00000565165.1* | c.1142+581A>G | CODING\_TRANSCRIPT\_INTRON\_VARIANT | 0.124 |

Squirls features

| Feature | Value |
| --- | --- |
| *Ri wt* donor | 5.98 |
| *ΔRi* canonical donor | 0.00 |
| *ΔRi wt* closest donor | 0.00 |
| Donor offset | 581 |
| max *Ri* cryptic donor window | 9.65 |
| *ΔRi* cryptic donor | 3.67 |
| phyloP | 0.19 |

Cryptic donor

Using cryptic donor site at *5:78,180,826*
would lead to addition
of 581
bases
to the coding sequence.

Canonical donor site

T
G
A
C
C
G
T
A
C
T
A
G
C
T
A
G
A
G
C
T
C
T
G
A
C
T
G
A
C
T
A
G
C
A
G
T
-3

-2

-1

1

2

3

4

5

6

c
a
g
g
t
a
c
c
t

Predicted cryptic donor site

T
G
A
C
C
G
T
A
C
T
A
G
C
T
A
G
A
G
C
T
C
T
G
A
C
T
G
A
C
T
A
G
C
A
G
T
579

.

.

.

582

.

.

.

.

.

587

.

g
t
g
a
t
a
a
g
t
g

**1:94,484,125 T>C**

***ABCA4***

Squirls score:
**0.122**

The variant overlaps with 2
transcripts:

| Transcript Accession | CDS Change | Variant Effect | Squirls Score |
| --- | --- | --- | --- |
| *ENST00000370225.3* | c.5196+1013A>G | CODING\_TRANSCRIPT\_INTRON\_VARIANT | 0.122 |
| *ENST00000536513.1* | c.6+5A>G | SPLICE\_REGION\_VARIANT | 0.008 |

Squirls features

| Feature | Value |
| --- | --- |
| *Ri wt* donor | 5.64 |
| *ΔRi* canonical donor | 0.00 |
| *ΔRi wt* closest donor | 0.00 |
| Donor offset | 1013 |
| max *Ri* cryptic donor window | 9.67 |
| *ΔRi* cryptic donor | 4.03 |
| phyloP | 0.00 |

Cryptic donor

Using cryptic donor site at *1:94,484,121*
would lead to addition
of 1009
bases
to the coding sequence.

Canonical donor site

T
G
A
C
C
G
T
A
C
T
A
G
C
T
A
G
A
G
C
T
C
T
G
A
C
T
G
A
C
T
A
G
C
A
G
T
-3

-2

-1

1

2

3

4

5

6

a
t
c
g
t
a
a
g
t

Predicted cryptic donor site

T
G
A
C
C
G
T
A
C
T
A
G
C
T
A
G
A
G
C
T
C
T
G
A
C
T
G
A
C
T
A
G
C
A
G
T
1007

.

.

.

1010

.

.

.

.

.

1015

.

a
t
g
g
t
g
a
a
t
g

**13:32,919,384 T>G**

***BRCA2***

Squirls score:
**0.120**

The variant overlaps with 2
transcripts:

| Transcript Accession | CDS Change | Variant Effect | Squirls Score |
| --- | --- | --- | --- |
| *ENST00000380152.3* | c.6937+594T>G | CODING\_TRANSCRIPT\_INTRON\_VARIANT | 0.120 |
| *ENST00000544455.1* | c.6937+594T>G | CODING\_TRANSCRIPT\_INTRON\_VARIANT | 0.120 |

Squirls features

| Feature | Value |
| --- | --- |
| *Ri wt* donor | 5.99 |
| *ΔRi* canonical donor | 0.00 |
| *ΔRi wt* closest donor | 0.00 |
| Donor offset | 594 |
| max *Ri* cryptic donor window | 10.13 |
| *ΔRi* cryptic donor | 4.14 |
| phyloP | 0.02 |

Cryptic donor

Using cryptic donor site at *13:32,919,380*
would lead to addition
of 590
bases
to the coding sequence.

Canonical donor site

T
G
A
C
C
G
T
A
C
T
A
G
C
T
A
G
A
G
C
T
C
T
G
A
C
T
G
A
C
T
A
G
C
A
G
T
-3

-2

-1

1

2

3

4

5

6

a
t
g
g
t
a
a
a
a

Predicted cryptic donor site

T
G
A
C
C
G
T
A
C
T
A
G
C
T
A
G
A
G
C
T
C
T
G
A
C
T
G
A
C
T
A
G
C
A
G
T
588

.

.

.

591

.

.

.

.

.

596

.

a
a
g
g
t
a
t
t
t
g

**1:94,495,923 C>T**

***ABCA4***

Squirls score:
**0.119**

The variant overlaps with 1
transcript:

| Transcript Accession | CDS Change | Variant Effect | Squirls Score |
| --- | --- | --- | --- |
| *ENST00000370225.3* | c.4352+61G>A | CODING\_TRANSCRIPT\_INTRON\_VARIANT | 0.119 |

Squirls features

| Feature | Value |
| --- | --- |
| *Ri wt* donor | 3.82 |
| *ΔRi* canonical donor | 0.00 |
| *ΔRi wt* closest donor | 0.00 |
| Donor offset | 61 |
| max *Ri* cryptic donor window | 9.28 |
| *ΔRi* cryptic donor | 5.46 |
| phyloP | -0.77 |

Cryptic donor

Using cryptic donor site at *1:94,495,920*
would lead to addition
of 58
bases
to the coding sequence.

Canonical donor site

T
G
A
C
C
G
T
A
C
T
A
G
C
T
A
G
A
G
C
T
C
T
G
A
C
T
G
A
C
T
A
G
C
A
G
T
-3

-2

-1

1

2

3

4

5

6

t
c
c
g
t
a
a
g
t

Predicted cryptic donor site

T
G
A
C
C
G
T
A
C
T
A
G
C
T
A
G
A
G
C
T
C
T
G
A
C
T
G
A
C
T
A
G
C
A
G
T
56

.

.

.

59

.

.

.

.

.

64

.

a
c
g
g
t
g
g
g
t
a

**16:56,927,219 C>T**

***SLC12A3***

Squirls score:
**0.118**

The variant overlaps with 4
transcripts:

| Transcript Accession | CDS Change | Variant Effect | Squirls Score |
| --- | --- | --- | --- |
| *ENST00000262502.5* | c.2518+253C>T | CODING\_TRANSCRIPT\_INTRON\_VARIANT | 0.118 |
| *ENST00000438926.2* | c.2548+253C>T | CODING\_TRANSCRIPT\_INTRON\_VARIANT | 0.118 |
| *ENST00000563236.1* | c.2521+253C>T | CODING\_TRANSCRIPT\_INTRON\_VARIANT | 0.118 |
| *ENST00000566786.1* | c.2545+253C>T | CODING\_TRANSCRIPT\_INTRON\_VARIANT | 0.118 |

Squirls features

| Feature | Value |
| --- | --- |
| *Ri wt* donor | 7.59 |
| *ΔRi* canonical donor | 0.00 |
| *ΔRi wt* closest donor | 0.00 |
| Donor offset | 253 |
| max *Ri* cryptic donor window | 10.32 |
| *ΔRi* cryptic donor | 2.73 |
| phyloP | 0.02 |

Cryptic donor

Using cryptic donor site at *16:56,927,218*
would lead to addition
of 252
bases
to the coding sequence.

Canonical donor site

T
G
A
C
C
G
T
A
C
T
A
G
C
T
A
G
A
G
C
T
C
T
G
A
C
T
G
A
C
T
A
G
C
A
G
T
-3

-2

-1

1

2

3

4

5

6

g
a
g
g
t
c
a
g
t

Predicted cryptic donor site

T
G
A
C
C
G
T
A
C
T
A
G
C
T
A
G
A
G
C
T
C
T
G
A
C
T
G
A
C
T
A
G
C
A
G
T
250

.

.

.

253

.

.

.

.

.

258

.

c
a
g
g
c
a
g
g
t
t

**4:108,945,190 G>T**

***HADH***

Squirls score:
**0.113**

The variant overlaps with 6
transcripts:

| Transcript Accession | CDS Change | Variant Effect | Squirls Score |
| --- | --- | --- | --- |
| *ENST00000309522.3* | c.636+471G>T | CODING\_TRANSCRIPT\_INTRON\_VARIANT | 0.113 |
| *ENST00000403312.1* | c.813+471G>T | CODING\_TRANSCRIPT\_INTRON\_VARIANT | 0.113 |
| *ENST00000454409.2* | c.648+471G>T | CODING\_TRANSCRIPT\_INTRON\_VARIANT | 0.113 |
| *ENST00000505878.1* | c.648+471G>T | CODING\_TRANSCRIPT\_INTRON\_VARIANT | 0.113 |
| *ENST00000507260.1* | n.336+471G>T | NON\_CODING\_TRANSCRIPT\_INTRON\_VARIANT | 0.113 |
| *ENST00000603302.1* | c.636+471G>T | CODING\_TRANSCRIPT\_INTRON\_VARIANT | 0.113 |

Squirls features

| Feature | Value |
| --- | --- |
| *Ri wt* donor | 11.27 |
| *ΔRi* canonical donor | 0.00 |
| *ΔRi wt* closest donor | 0.00 |
| Donor offset | 471 |
| max *Ri* cryptic donor window | 10.21 |
| *ΔRi* cryptic donor | -1.06 |
| phyloP | -0.31 |

Cryptic donor

Using cryptic donor site at *4:108,945,189*
would lead to addition
of 470
bases
to the coding sequence.

Canonical donor site

T
G
A
C
C
G
T
A
C
T
A
G
C
T
A
G
A
G
C
T
C
T
G
A
C
T
G
A
C
T
A
G
C
A
G
T
-3

-2

-1

1

2

3

4

5

6

a
a
g
g
t
a
a
g
a

Predicted cryptic donor site

T
G
A
C
C
G
T
A
C
T
A
G
C
T
A
G
A
G
C
T
C
T
G
A
C
T
G
A
C
T
A
G
C
A
G
T
468

.

.

.

471

.

.

.

.

.

476

.

c
c
g
g
g
a
a
g
t
t

**X:154,130,719 C>T**

***F8***

Squirls score:
**0.112**

The variant overlaps with 1
transcript:

| Transcript Accession | CDS Change | Variant Effect | Squirls Score |
| --- | --- | --- | --- |
| *ENST00000360256.4* | c.5999-277G>A | CODING\_TRANSCRIPT\_INTRON\_VARIANT | 0.112 |

Squirls features

| Feature | Value |
| --- | --- |
| *Ri wt* donor | 8.83 |
| *ΔRi* canonical donor | 0.00 |
| *ΔRi wt* closest donor | 0.00 |
| Donor offset | -394 |
| max *Ri* cryptic donor window | 9.14 |
| *ΔRi* cryptic donor | 0.32 |
| phyloP | 0.51 |

Cryptic donor

Using cryptic donor site at *X:154,130,716*
would lead to addition
of 1459
bases
to the coding sequence.

Canonical donor site

T
G
A
C
C
G
T
A
C
T
A
G
C
T
A
G
A
G
C
T
C
T
G
A
C
T
G
A
C
T
A
G
C
A
G
T
-3

-2

-1

1

2

3

4

5

6

c
a
g
g
t
a
t
g
a

Predicted cryptic donor site

T
G
A
C
C
G
T
A
C
T
A
G
C
T
A
G
A
G
C
T
C
T
G
A
C
T
G
A
C
T
A
G
C
A
G
T
1457

.

.

.

1460

.

.

.

.

.

1465

.

a
a
t
g
t
a
g
g
t
a

**X:31,200,680 A>T**

***DMD***

Squirls score:
**0.109**

The variant overlaps with 14
transcripts:

| Transcript Accession | CDS Change | Variant Effect | Squirls Score |
| --- | --- | --- | --- |
| *ENST00000343523.2* | c.2594+175T>A | CODING\_TRANSCRIPT\_INTRON\_VARIANT | 0.109 |
| *ENST00000357033.4* | c.9974+175T>A | CODING\_TRANSCRIPT\_INTRON\_VARIANT | 0.109 |
| *ENST00000358062.2* | c.3062+175T>A | CODING\_TRANSCRIPT\_INTRON\_VARIANT | 0.109 |
| *ENST00000359836.1* | c.2594+175T>A | CODING\_TRANSCRIPT\_INTRON\_VARIANT | 0.109 |
| *ENST00000361471.4* | c.770+175T>A | CODING\_TRANSCRIPT\_INTRON\_VARIANT | 0.109 |
| *ENST00000378677.2* | c.9962+175T>A | CODING\_TRANSCRIPT\_INTRON\_VARIANT | 0.109 |
| *ENST00000378680.2* | c.770+175T>A | CODING\_TRANSCRIPT\_INTRON\_VARIANT | 0.109 |
| *ENST00000378702.4* | c.770+175T>A | CODING\_TRANSCRIPT\_INTRON\_VARIANT | 0.109 |
| *ENST00000378705.3* | c.344+175T>A | CODING\_TRANSCRIPT\_INTRON\_VARIANT | 0.109 |
| *ENST00000378707.3* | c.2594+175T>A | CODING\_TRANSCRIPT\_INTRON\_VARIANT | 0.109 |
| *ENST00000378723.3* | c.770+175T>A | CODING\_TRANSCRIPT\_INTRON\_VARIANT | 0.109 |
| *ENST00000474231.1* | c.2594+175T>A | CODING\_TRANSCRIPT\_INTRON\_VARIANT | 0.109 |
| *ENST00000475732.1* | n.190+175T>A | NON\_CODING\_TRANSCRIPT\_INTRON\_VARIANT | 0.109 |
| *ENST00000541735.1* | c.2594+175T>A | CODING\_TRANSCRIPT\_INTRON\_VARIANT | 0.109 |

Squirls features

| Feature | Value |
| --- | --- |
| *Ri wt* donor | 5.54 |
| *ΔRi* canonical donor | 0.00 |
| *ΔRi wt* closest donor | 0.00 |
| Donor offset | 175 |
| max *Ri* cryptic donor window | 7.97 |
| *ΔRi* cryptic donor | 2.43 |
| phyloP | 1.51 |

Cryptic donor

Using cryptic donor site at *X:31,200,678*
would lead to addition
of 173
bases
to the coding sequence.

Canonical donor site

T
G
A
C
C
G
T
A
C
T
A
G
C
T
A
G
A
G
C
T
C
T
G
A
C
T
G
A
C
T
A
G
C
A
G
T
-3

-2

-1

1

2

3

4

5

6

c
a
g
g
t
a
t
t
a

Predicted cryptic donor site

T
G
A
C
C
G
T
A
C
T
A
G
C
T
A
G
A
G
C
T
C
T
G
A
C
T
G
A
C
T
A
G
C
A
G
T
171

.

.

.

174

.

.

.

.

.

179

.

t
a
g
g
t
t
a
t
t
a

**1:94,509,799 G>A**

***ABCA4***

Squirls score:
**0.105**

The variant overlaps with 1
transcript:

| Transcript Accession | CDS Change | Variant Effect | Squirls Score |
| --- | --- | --- | --- |
| *ENST00000370225.3* | c.3050+370C>T | CODING\_TRANSCRIPT\_INTRON\_VARIANT | 0.105 |

Squirls features

| Feature | Value |
| --- | --- |
| *Ri wt* donor | 5.50 |
| *ΔRi* canonical donor | 0.00 |
| *ΔRi wt* closest donor | 0.00 |
| Donor offset | 370 |
| max *Ri* cryptic donor window | 7.95 |
| *ΔRi* cryptic donor | 2.45 |
| phyloP | -1.46 |

Cryptic donor

Using cryptic donor site at *1:94,509,798*
would lead to addition
of 369
bases
to the coding sequence.

Canonical donor site

T
G
A
C
C
G
T
A
C
T
A
G
C
T
A
G
A
G
C
T
C
T
G
A
C
T
G
A
C
T
A
G
C
A
G
T
-3

-2

-1

1

2

3

4

5

6

c
c
a
g
t
a
a
g
c

Predicted cryptic donor site

T
G
A
C
C
G
T
A
C
T
A
G
C
T
A
G
A
G
C
T
C
T
G
A
C
T
G
A
C
T
A
G
C
A
G
T
367

.

.

.

370

.

.

.

.

.

375

.

a
a
g
g
c
a
a
a
c
t

**X:31,219,364 G>A**

***DMD***

Squirls score:
**0.100**

The variant overlaps with 13
transcripts:

| Transcript Accession | CDS Change | Variant Effect | Squirls Score |
| --- | --- | --- | --- |
| *ENST00000343523.2* | c.2427+2714C>T | CODING\_TRANSCRIPT\_INTRON\_VARIANT | 0.100 |
| *ENST00000357033.4* | c.9807+2714C>T | CODING\_TRANSCRIPT\_INTRON\_VARIANT | 0.100 |
| *ENST00000358062.2* | c.2895+2714C>T | CODING\_TRANSCRIPT\_INTRON\_VARIANT | 0.100 |
| *ENST00000359836.1* | c.2427+2714C>T | CODING\_TRANSCRIPT\_INTRON\_VARIANT | 0.100 |
| *ENST00000361471.4* | c.603+2714C>T | CODING\_TRANSCRIPT\_INTRON\_VARIANT | 0.100 |
| *ENST00000378677.2* | c.9795+2714C>T | CODING\_TRANSCRIPT\_INTRON\_VARIANT | 0.100 |
| *ENST00000378680.2* | c.603+2714C>T | CODING\_TRANSCRIPT\_INTRON\_VARIANT | 0.100 |
| *ENST00000378702.4* | c.603+2714C>T | CODING\_TRANSCRIPT\_INTRON\_VARIANT | 0.100 |
| *ENST00000378705.3* | c.177+2714C>T | CODING\_TRANSCRIPT\_INTRON\_VARIANT | 0.100 |
| *ENST00000378707.3* | c.2427+2714C>T | CODING\_TRANSCRIPT\_INTRON\_VARIANT | 0.100 |
| *ENST00000378723.3* | c.603+2714C>T | CODING\_TRANSCRIPT\_INTRON\_VARIANT | 0.100 |
| *ENST00000474231.1* | c.2427+2714C>T | CODING\_TRANSCRIPT\_INTRON\_VARIANT | 0.100 |
| *ENST00000541735.1* | c.2427+2714C>T | CODING\_TRANSCRIPT\_INTRON\_VARIANT | 0.100 |

Squirls features

| Feature | Value |
| --- | --- |
| *Ri wt* donor | 5.51 |
| *ΔRi* canonical donor | 0.00 |
| *ΔRi wt* closest donor | 0.00 |
| Donor offset | 2714 |
| max *Ri* cryptic donor window | 8.92 |
| *ΔRi* cryptic donor | 3.41 |
| phyloP | 0.56 |

Cryptic donor

Using cryptic donor site at *X:31,219,363*
would lead to addition
of 2713
bases
to the coding sequence.

Canonical donor site

T
G
A
C
C
G
T
A
C
T
A
G
C
T
A
G
A
G
C
T
C
T
G
A
C
T
G
A
C
T
A
G
C
A
G
T
-3

-2

-1

1

2

3

4

5

6

t
t
t
g
t
a
a
g
t

Predicted cryptic donor site

T
G
A
C
C
G
T
A
C
T
A
G
C
T
A
G
A
G
C
T
C
T
G
A
C
T
G
A
C
T
A
G
C
A
G
T
2711

.

.

.

2714

.

.

.

.

.

2719

.

c
a
g
g
c
a
t
g
g
t

**16:2,107,460 C>T**

***TSC2***

Squirls score:
**0.095**

The variant overlaps with 9
transcripts:

| Transcript Accession | CDS Change | Variant Effect | Squirls Score |
| --- | --- | --- | --- |
| *ENST00000219476.3* | c.848+281C>T | CODING\_TRANSCRIPT\_INTRON\_VARIANT | 0.095 |
| *ENST00000350773.4* | c.848+281C>T | CODING\_TRANSCRIPT\_INTRON\_VARIANT | 0.095 |
| *ENST00000353929.4* | c.848+281C>T | CODING\_TRANSCRIPT\_INTRON\_VARIANT | 0.095 |
| *ENST00000382538.6* | c.701+281C>T | CODING\_TRANSCRIPT\_INTRON\_VARIANT | 0.095 |
| *ENST00000401874.2* | c.848+281C>T | CODING\_TRANSCRIPT\_INTRON\_VARIANT | 0.095 |
| *ENST00000439117.2* | c.\*147+281C>T | THREE\_PRIME\_UTR\_INTRON\_VARIANT | 0.095 |
| *ENST00000439673.2* | c.737+281C>T | CODING\_TRANSCRIPT\_INTRON\_VARIANT | 0.095 |
| *ENST00000467949.1* | c.284+281C>T | CODING\_TRANSCRIPT\_INTRON\_VARIANT | 0.095 |
| *ENST00000568454.1* | c.881+281C>T | CODING\_TRANSCRIPT\_INTRON\_VARIANT | 0.095 |

Squirls features

| Feature | Value |
| --- | --- |
| *ΔRi* canonical acceptor | 0.00 |
| *ΔRi* cryptic acceptor | 2.35 |
| Creates AG in *AGEZ* | No |
| Acceptor offset | 355 |
| Exon length | -1 |
| ESRSeq | -2.12 |
| SMS | -2.21 |
| phyloP | -0.49 |

Cryptic acceptor

Using cryptic acceptor site at *16:2,107,459*
would lead to addition
of 1289
bases
to the coding sequence.

Canonical acceptor site

G
C
A
T
G
A
C
T
G
A
C
T
G
A
C
T
G
A
C
T
G
A
C
T
G
A
C
T
G
A
C
T
G
A
C
T
A
G
C
T
A
G
C
T
A
G
C
T
A
G
C
T
A
G
C
T
A
G
C
T
A
G
C
T
A
G
C
T
G
A
C
T
G
A
C
T
G
A
C
T
G
A
C
T
G
A
C
T
G
A
T
C
C
T
G
A
A
T
C
G
T
C
A
G
C
G
A
T
-25
.
.
.
.
-20
.
.
.
.
-15
.
.
.
.
.
-9
-8
-7
-6
-5
-4
-3
-2
-1
1
2
c
c
g
t
c
t
c
t
c
t
g
g
g
g
a
a
c
a
c
t
t
t
t
a
g
a
g

Predicted cryptic acceptor site

G
C
A
T
G
A
C
T
G
A
C
T
G
A
C
T
G
A
C
T
G
A
C
T
G
A
C
T
G
A
C
T
G
A
C
T
A
G
C
T
A
G
C
T
A
G
C
T
A
G
C
T
A
G
C
T
A
G
C
T
A
G
C
T
A
G
C
T
G
A
C
T
G
A
C
T
G
A
C
T
G
A
C
T
G
A
C
T
G
A
T
C
C
T
G
A
A
T
C
G
T
C
A
G
C
G
A
T
-1314
.
.
.
.
.
-1309
.
.
.
.
.
-1304
.
.
.
.
.
-1299
.
.
.
.
.
-1294
.
.
.
.
.
-1289
.
.
c
c
c
c
a
a
c
t
a
c
t
t
a
g
c
c
t
g
t
t
a
c
a
a
g
g
c
t

**6:51,747,238 T>C**

***PKHD1***

Squirls score:
**0.091**

The variant overlaps with 2
transcripts:

| Transcript Accession | CDS Change | Variant Effect | Squirls Score |
| --- | --- | --- | --- |
| *ENST00000340994.4* | c.7350+653A>G | CODING\_TRANSCRIPT\_INTRON\_VARIANT | 0.091 |
| *ENST00000371117.3* | c.7350+653A>G | CODING\_TRANSCRIPT\_INTRON\_VARIANT | 0.091 |

Squirls features

| Feature | Value |
| --- | --- |
| *Ri wt* donor | 12.69 |
| *ΔRi* canonical donor | 0.00 |
| *ΔRi wt* closest donor | 0.00 |
| Donor offset | 653 |
| max *Ri* cryptic donor window | 10.49 |
| *ΔRi* cryptic donor | -2.20 |
| phyloP | -0.49 |

Cryptic donor

Using cryptic donor site at *6:51,747,238*
would lead to addition
of 653
bases
to the coding sequence.

Canonical donor site

T
G
A
C
C
G
T
A
C
T
A
G
C
T
A
G
A
G
C
T
C
T
G
A
C
T
G
A
C
T
A
G
C
A
G
T
-3

-2

-1

1

2

3

4

5

6

a
a
g
g
t
a
a
g
t

Predicted cryptic donor site

T
G
A
C
C
G
T
A
C
T
A
G
C
T
A
G
A
G
C
T
C
T
G
A
C
T
G
A
C
T
A
G
C
A
G
T
651

.

.

.

654

.

.

.

.

.

659

.

a
t
g
a
t
a
a
g
t
g

**1:216,041,166 G>C**

***USH2A***

Squirls score:
**0.091**

The variant overlaps with 2
transcripts:

| Transcript Accession | CDS Change | Variant Effect | Squirls Score |
| --- | --- | --- | --- |
| *ENST00000307340.3* | c.8682-654C>G | CODING\_TRANSCRIPT\_INTRON\_VARIANT | 0.091 |
| *ENST00000366943.2* | c.8682-654C>G | CODING\_TRANSCRIPT\_INTRON\_VARIANT | 0.091 |

Squirls features

| Feature | Value |
| --- | --- |
| *ΔRi* canonical acceptor | 0.00 |
| *ΔRi* cryptic acceptor | 1.71 |
| Creates AG in *AGEZ* | No |
| Acceptor offset | -654 |
| Exon length | -1 |
| ESRSeq | 1.45 |
| SMS | 0.27 |
| phyloP | 3.27 |

Cryptic acceptor

Using cryptic acceptor site at *1:216,041,187*
would lead to addition
of 633
bases
to the coding sequence.

Canonical acceptor site

G
C
A
T
G
A
C
T
G
A
C
T
G
A
C
T
G
A
C
T
G
A
C
T
G
A
C
T
G
A
C
T
G
A
C
T
A
G
C
T
A
G
C
T
A
G
C
T
A
G
C
T
A
G
C
T
A
G
C
T
A
G
C
T
A
G
C
T
G
A
C
T
G
A
C
T
G
A
C
T
G
A
C
T
G
A
C
T
G
A
T
C
C
T
G
A
A
T
C
G
T
C
A
G
C
G
A
T
-25
.
.
.
.
-20
.
.
.
.
-15
.
.
.
.
.
-9
-8
-7
-6
-5
-4
-3
-2
-1
1
2
t
t
t
t
t
t
t
a
a
c
a
a
a
a
c
a
a
c
a
t
t
t
t
a
g
g
t

Predicted cryptic acceptor site

G
C
A
T
G
A
C
T
G
A
C
T
G
A
C
T
G
A
C
T
G
A
C
T
G
A
C
T
G
A
C
T
G
A
C
T
A
G
C
T
A
G
C
T
A
G
C
T
A
G
C
T
A
G
C
T
A
G
C
T
A
G
C
T
A
G
C
T
G
A
C
T
G
A
C
T
G
A
C
T
G
A
C
T
G
A
C
T
G
A
T
C
C
T
G
A
A
T
C
G
T
C
A
G
C
G
A
T
-658
.
.
.
.
.
-653
.
.
.
.
.
-648
.
.
.
.
.
-643
.
.
.
.
.
-638
.
.
.
.
.
-633
.
.
a
t
g
t
c
a
g
t
t
t
t
t
g
c
c
t
t
c
t
g
c
a
a
a
g
t
a
g

**19:54,633,399 C>G**

***PRPF31***

Squirls score:
**0.088**

The variant overlaps with 4
transcripts:

| Transcript Accession | CDS Change | Variant Effect | Squirls Score |
| --- | --- | --- | --- |
| *ENST00000321030.4* | c.1374+654C>G | CODING\_TRANSCRIPT\_INTRON\_VARIANT | 0.088 |
| *ENST00000391755.1* | c.1356+654C>G | CODING\_TRANSCRIPT\_INTRON\_VARIANT | 0.088 |
| *ENST00000419967.1* | c.1306+654C>G | CODING\_TRANSCRIPT\_INTRON\_VARIANT | 0.088 |
| *ENST00000466404.1* | n.1434+654C>G | NON\_CODING\_TRANSCRIPT\_INTRON\_VARIANT | 0.088 |

Squirls features

| Feature | Value |
| --- | --- |
| *Ri wt* donor | 5.98 |
| *ΔRi* canonical donor | 0.00 |
| *ΔRi wt* closest donor | 0.00 |
| Donor offset | 654 |
| max *Ri* cryptic donor window | 8.53 |
| *ΔRi* cryptic donor | 2.55 |
| phyloP | -0.69 |

Cryptic donor

Using cryptic donor site at *19:54,633,399*
would lead to addition
of 654
bases
to the coding sequence.

Canonical donor site

T
G
A
C
C
G
T
A
C
T
A
G
C
T
A
G
A
G
C
T
C
T
G
A
C
T
G
A
C
T
A
G
C
A
G
T
-3

-2

-1

1

2

3

4

5

6

c
a
g
g
t
a
c
c
t

Predicted cryptic donor site

T
G
A
C
C
G
T
A
C
T
A
G
C
T
A
G
A
G
C
T
C
T
G
A
C
T
G
A
C
T
A
G
C
A
G
T
652

.

.

.

655

.

.

.

.

.

660

.

c
a
g
c
t
c
a
g
t
g

**19:11,233,632 G>A**

***LDLR***

Squirls score:
**0.079**

The variant overlaps with 7
transcripts:

| Transcript Accession | CDS Change | Variant Effect | Squirls Score |
| --- | --- | --- | --- |
| *ENST00000252444.5* | c.2395-218G>A | CODING\_TRANSCRIPT\_INTRON\_VARIANT | 0.079 |
| *ENST00000455727.2* | c.1637-218G>A | CODING\_TRANSCRIPT\_INTRON\_VARIANT | 0.079 |
| *ENST00000535915.1* | c.2018-218G>A | CODING\_TRANSCRIPT\_INTRON\_VARIANT | 0.079 |
| *ENST00000545707.1* | c.1607-218G>A | CODING\_TRANSCRIPT\_INTRON\_VARIANT | 0.030 |
| *ENST00000557933.1* | c.2141-218G>A | CODING\_TRANSCRIPT\_INTRON\_VARIANT | 0.079 |
| *ENST00000558013.1* | c.2141-218G>A | CODING\_TRANSCRIPT\_INTRON\_VARIANT | 0.079 |
| *ENST00000558518.1* | c.2141-218G>A | CODING\_TRANSCRIPT\_INTRON\_VARIANT | 0.079 |

Squirls features

| Feature | Value |
| --- | --- |
| *Ri wt* donor | 8.10 |
| *ΔRi* canonical donor | 0.00 |
| *ΔRi wt* closest donor | 0.00 |
| Donor offset | -389 |
| max *Ri* cryptic donor window | 8.59 |
| *ΔRi* cryptic donor | 0.49 |
| phyloP | -1.38 |

Cryptic donor

Using cryptic donor site at *19:11,233,630*
would lead to addition
of 2432
bases
to the coding sequence.

Canonical donor site

T
G
A
C
C
G
T
A
C
T
A
G
C
T
A
G
A
G
C
T
C
T
G
A
C
T
G
A
C
T
A
G
C
A
G
T
-3

-2

-1

1

2

3

4

5

6

c
a
g
g
t
g
t
g
g

Predicted cryptic donor site

T
G
A
C
C
G
T
A
C
T
A
G
C
T
A
G
A
G
C
T
C
T
G
A
C
T
G
A
C
T
A
G
C
A
G
T
2430

.

.

.

2433

.

.

.

.

.

2438

.

c
a
g
g
t
g
t
g
c
a

**8:87,617,644 C>T**

***CNGB3***

Squirls score:
**0.071**

The variant overlaps with 1
transcript:

| Transcript Accession | CDS Change | Variant Effect | Squirls Score |
| --- | --- | --- | --- |
| *ENST00000320005.5* | c.1663-1205G>A | CODING\_TRANSCRIPT\_INTRON\_VARIANT | 0.071 |

Squirls features

| Feature | Value |
| --- | --- |
| *Ri wt* donor | 11.87 |
| *ΔRi* canonical donor | 0.00 |
| *ΔRi wt* closest donor | 0.00 |
| Donor offset | -1324 |
| max *Ri* cryptic donor window | 9.26 |
| *ΔRi* cryptic donor | -2.62 |
| phyloP | 0.28 |

Cryptic donor

Using cryptic donor site at *8:87,617,641*
would lead to addition
of 6169
bases
to the coding sequence.

Canonical donor site

T
G
A
C
C
G
T
A
C
T
A
G
C
T
A
G
A
G
C
T
C
T
G
A
C
T
G
A
C
T
A
G
C
A
G
T
-3

-2

-1

1

2

3

4

5

6

a
a
g
g
t
g
a
g
t

Predicted cryptic donor site

T
G
A
C
C
G
T
A
C
T
A
G
C
T
A
G
A
G
C
T
C
T
G
A
C
T
G
A
C
T
A
G
C
A
G
T
6167

.

.

.

6170

.

.

.

.

.

6175

.

c
a
t
g
t
a
g
g
t
a

**X:154,249,118 T>C**

***F8***

Squirls score:
**0.071**

The variant overlaps with 3
transcripts:

| Transcript Accession | CDS Change | Variant Effect | Squirls Score |
| --- | --- | --- | --- |
| *ENST00000360256.4* | c.143+1567A>G | CODING\_TRANSCRIPT\_INTRON\_VARIANT | 0.032 |
| *ENST00000423959.1* | c.38+5937A>G | CODING\_TRANSCRIPT\_INTRON\_VARIANT | 0.071 |
| *ENST00000453950.1* | c.125+1567A>G | CODING\_TRANSCRIPT\_INTRON\_VARIANT | 0.032 |

Squirls features

| Feature | Value |
| --- | --- |
| *Ri wt* donor | 7.27 |
| *ΔRi* canonical donor | 0.00 |
| *ΔRi wt* closest donor | 0.00 |
| Donor offset | 5937 |
| max *Ri* cryptic donor window | 9.26 |
| *ΔRi* cryptic donor | 2.00 |
| phyloP | -1.13 |

Cryptic donor

Using cryptic donor site at *X:154,249,118*
would lead to addition
of 5937
bases
to the coding sequence.

Canonical donor site

T
G
A
C
C
G
T
A
C
T
A
G
C
T
A
G
A
G
C
T
C
T
G
A
C
T
G
A
C
T
A
G
C
A
G
T
-3

-2

-1

1

2

3

4

5

6

g
g
g
g
t
g
a
g
g

Predicted cryptic donor site

T
G
A
C
C
G
T
A
C
T
A
G
C
T
A
G
A
G
C
T
C
T
G
A
C
T
G
A
C
T
A
G
C
A
G
T
5935

.

.

.

5938

.

.

.

.

.

5943

.

g
c
g
a
t
a
a
g
t
g

**X:66,942,551 A>G**

***AR***

Squirls score:
**0.064**

The variant overlaps with 3
transcripts:

| Transcript Accession | CDS Change | Variant Effect | Squirls Score |
| --- | --- | --- | --- |
| *ENST00000374690.3* | c.2450-118A>G | CODING\_TRANSCRIPT\_INTRON\_VARIANT | 0.064 |
| *ENST00000396043.2* | c.854-118A>G | CODING\_TRANSCRIPT\_INTRON\_VARIANT | 0.064 |
| *ENST00000396044.3* | c.2174-977A>G | CODING\_TRANSCRIPT\_INTRON\_VARIANT | 0.020 |

Squirls features

| Feature | Value |
| --- | --- |
| *Ri wt* donor | 6.00 |
| *ΔRi* canonical donor | 0.00 |
| *ΔRi wt* closest donor | 0.00 |
| Donor offset | -276 |
| max *Ri* cryptic donor window | 7.23 |
| *ΔRi* cryptic donor | 1.23 |
| phyloP | 0.71 |

Cryptic donor

Using cryptic donor site at *X:66,942,552*
would lead to addition
of 747
bases
to the coding sequence.

Canonical donor site

T
G
A
C
C
G
T
A
C
T
A
G
C
T
A
G
A
G
C
T
C
T
G
A
C
T
G
A
C
T
A
G
C
A
G
T
-3

-2

-1

1

2

3

4

5

6

t
t
a
g
t
a
a
g
t

Predicted cryptic donor site

T
G
A
C
C
G
T
A
C
T
A
G
C
T
A
G
A
G
C
T
C
T
G
A
C
T
G
A
C
T
A
G
C
A
G
T
745

.

.

.

748

.

.

.

.

.

753

.

c
a
a
g
t
a
g
a
t
g

**1:94,483,922 G>T**

***ABCA4***

Squirls score:
**0.063**

The variant overlaps with 2
transcripts:

| Transcript Accession | CDS Change | Variant Effect | Squirls Score |
| --- | --- | --- | --- |
| *ENST00000370225.3* | c.5196+1216C>A | CODING\_TRANSCRIPT\_INTRON\_VARIANT | 0.063 |
| *ENST00000536513.1* | c.6+208C>A | CODING\_TRANSCRIPT\_INTRON\_VARIANT | 0.028 |

Squirls features

| Feature | Value |
| --- | --- |
| *Ri wt* donor | 5.64 |
| *ΔRi* canonical donor | 0.00 |
| *ΔRi wt* closest donor | 0.00 |
| Donor offset | 1216 |
| max *Ri* cryptic donor window | 7.68 |
| *ΔRi* cryptic donor | 2.03 |
| phyloP | 0.15 |

Cryptic donor

Using cryptic donor site at *1:94,483,919*
would lead to addition
of 1213
bases
to the coding sequence.

Canonical donor site

T
G
A
C
C
G
T
A
C
T
A
G
C
T
A
G
A
G
C
T
C
T
G
A
C
T
G
A
C
T
A
G
C
A
G
T
-3

-2

-1

1

2

3

4

5

6

a
t
c
g
t
a
a
g
t

Predicted cryptic donor site

T
G
A
C
C
G
T
A
C
T
A
G
C
T
A
G
A
G
C
T
C
T
G
A
C
T
G
A
C
T
A
G
C
A
G
T
1211

.

.

.

1214

.

.

.

.

.

1219

.

c
a
g
g
t
a
c
c
a
a

**X:107,820,081 A>G**

***COL4A5***

Squirls score:
**0.062**

The variant overlaps with 2
transcripts:

| Transcript Accession | CDS Change | Variant Effect | Squirls Score |
| --- | --- | --- | --- |
| *ENST00000328300.6* | c.609+879A>G | CODING\_TRANSCRIPT\_INTRON\_VARIANT | 0.062 |
| *ENST00000361603.2* | c.609+879A>G | CODING\_TRANSCRIPT\_INTRON\_VARIANT | 0.062 |

Squirls features

| Feature | Value |
| --- | --- |
| *Ri wt* donor | 8.84 |
| *ΔRi* canonical donor | 0.00 |
| *ΔRi wt* closest donor | 0.00 |
| Donor offset | 879 |
| max *Ri* cryptic donor window | 10.46 |
| *ΔRi* cryptic donor | 1.62 |
| phyloP | -0.84 |

Cryptic donor

Using cryptic donor site at *X:107,820,081*
would lead to addition
of 879
bases
to the coding sequence.

Canonical donor site

T
G
A
C
C
G
T
A
C
T
A
G
C
T
A
G
A
G
C
T
C
T
G
A
C
T
G
A
C
T
A
G
C
A
G
T
-3

-2

-1

1

2

3

4

5

6

a
t
g
g
t
a
a
g
c

Predicted cryptic donor site

T
G
A
C
C
G
T
A
C
T
A
G
C
T
A
G
A
G
C
T
C
T
G
A
C
T
G
A
C
T
A
G
C
A
G
T
877

.

.

.

880

.

.

.

.

.

885

.

a
a
g
a
t
g
a
g
a
g

**9:98,226,337 T>C**

***PTCH1***

Squirls score:
**0.053**

The variant overlaps with 8
transcripts:

| Transcript Accession | CDS Change | Variant Effect | Squirls Score |
| --- | --- | --- | --- |
| *ENST00000331920.6* | c.2561-2057A>G | CODING\_TRANSCRIPT\_INTRON\_VARIANT | 0.053 |
| *ENST00000375274.2* | c.2558-2057A>G | CODING\_TRANSCRIPT\_INTRON\_VARIANT | 0.053 |
| *ENST00000375290.2* | c.\*869-2057A>G | THREE\_PRIME\_UTR\_INTRON\_VARIANT | 0.053 |
| *ENST00000418258.1* | c.2108-2057A>G | CODING\_TRANSCRIPT\_INTRON\_VARIANT | 0.053 |
| *ENST00000421141.1* | c.2108-2057A>G | CODING\_TRANSCRIPT\_INTRON\_VARIANT | 0.053 |
| *ENST00000429896.2* | c.2108-2057A>G | CODING\_TRANSCRIPT\_INTRON\_VARIANT | 0.053 |
| *ENST00000430669.2* | c.2363-2057A>G | CODING\_TRANSCRIPT\_INTRON\_VARIANT | 0.053 |
| *ENST00000437951.1* | c.2363-2057A>G | CODING\_TRANSCRIPT\_INTRON\_VARIANT | 0.053 |

Squirls features

| Feature | Value |
| --- | --- |
| *Ri wt* donor | 8.83 |
| *ΔRi* canonical donor | 0.00 |
| *ΔRi wt* closest donor | 0.00 |
| Donor offset | -2200 |
| max *Ri* cryptic donor window | 8.90 |
| *ΔRi* cryptic donor | 0.07 |
| phyloP | -0.10 |

Cryptic donor

Using cryptic donor site at *9:98,226,337*
would lead to addition
of 3061
bases
to the coding sequence.

Canonical donor site

T
G
A
C
C
G
T
A
C
T
A
G
C
T
A
G
A
G
C
T
C
T
G
A
C
T
G
A
C
T
A
G
C
A
G
T
-3

-2

-1

1

2

3

4

5

6

a
g
g
g
t
a
a
g
a

Predicted cryptic donor site

T
G
A
C
C
G
T
A
C
T
A
G
C
T
A
G
A
G
C
T
C
T
G
A
C
T
G
A
C
T
A
G
C
A
G
T
3059

.

.

.

3062

.

.

.

.

.

3067

.

c
a
g
a
t
a
g
g
a
g

**1:94,484,082 T>C**

***ABCA4***

Squirls score:
**0.052**

The variant overlaps with 2
transcripts:

| Transcript Accession | CDS Change | Variant Effect | Squirls Score |
| --- | --- | --- | --- |
| *ENST00000370225.3* | c.5196+1056A>G | CODING\_TRANSCRIPT\_INTRON\_VARIANT | 0.052 |
| *ENST00000536513.1* | c.6+48A>G | CODING\_TRANSCRIPT\_INTRON\_VARIANT | 0.025 |

Squirls features

| Feature | Value |
| --- | --- |
| *Ri wt* donor | 5.64 |
| *ΔRi* canonical donor | 0.00 |
| *ΔRi wt* closest donor | 0.00 |
| Donor offset | 1056 |
| max *Ri* cryptic donor window | 7.41 |
| *ΔRi* cryptic donor | 1.76 |
| phyloP | -0.13 |

Cryptic donor

Using cryptic donor site at *1:94,484,083*
would lead to addition
of 1057
bases
to the coding sequence.

Canonical donor site

T
G
A
C
C
G
T
A
C
T
A
G
C
T
A
G
A
G
C
T
C
T
G
A
C
T
G
A
C
T
A
G
C
A
G
T
-3

-2

-1

1

2

3

4

5

6

a
t
c
g
t
a
a
g
t

Predicted cryptic donor site

T
G
A
C
C
G
T
A
C
T
A
G
C
T
A
G
A
G
C
T
C
T
G
A
C
T
G
A
C
T
A
G
C
A
G
T
1055

.

.

.

1058

.

.

.

.

.

1063

.

t
t
a
g
t
a
a
g
c
g

**10:79,783,995 G>A**

***POLR3A***

Squirls score:
**0.049**

The variant overlaps with 1
transcript:

| Transcript Accession | CDS Change | Variant Effect | Squirls Score |
| --- | --- | --- | --- |
| *ENST00000372371.3* | c.645+312C>T | CODING\_TRANSCRIPT\_INTRON\_VARIANT | 0.049 |

Squirls features

| Feature | Value |
| --- | --- |
| *Ri wt* donor | 10.33 |
| *ΔRi* canonical donor | 0.00 |
| *ΔRi wt* closest donor | 0.00 |
| Donor offset | 312 |
| max *Ri* cryptic donor window | 10.24 |
| *ΔRi* cryptic donor | -0.09 |
| phyloP | -0.14 |

Cryptic donor

Using cryptic donor site at *10:79,783,994*
would lead to addition
of 311
bases
to the coding sequence.

Canonical donor site

T
G
A
C
C
G
T
A
C
T
A
G
C
T
A
G
A
G
C
T
C
T
G
A
C
T
G
A
C
T
A
G
C
A
G
T
-3

-2

-1

1

2

3

4

5

6

c
a
g
g
t
g
a
g
c

Predicted cryptic donor site

T
G
A
C
C
G
T
A
C
T
A
G
C
T
A
G
A
G
C
T
C
T
G
A
C
T
G
A
C
T
A
G
C
A
G
T
309

.

.

.

312

.

.

.

.

.

317

.

c
a
g
g
c
a
t
g
t
t

**5:37,031,001 C>T**

***NIPBL***

Squirls score:
**0.049**

The variant overlaps with 2
transcripts:

| Transcript Accession | CDS Change | Variant Effect | Squirls Score |
| --- | --- | --- | --- |
| *ENST00000282516.8* | c.5862+3487C>T | CODING\_TRANSCRIPT\_INTRON\_VARIANT | 0.049 |
| *ENST00000448238.2* | c.5862+3487C>T | CODING\_TRANSCRIPT\_INTRON\_VARIANT | 0.049 |

Squirls features

| Feature | Value |
| --- | --- |
| *Ri wt* donor | 7.03 |
| *ΔRi* canonical donor | 0.00 |
| *ΔRi wt* closest donor | 0.00 |
| Donor offset | 3487 |
| max *Ri* cryptic donor window | 8.59 |
| *ΔRi* cryptic donor | 1.57 |
| phyloP | 0.30 |

Cryptic donor

Using cryptic donor site at *5:37,031,000*
would lead to addition
of 3486
bases
to the coding sequence.

Canonical donor site

T
G
A
C
C
G
T
A
C
T
A
G
C
T
A
G
A
G
C
T
C
T
G
A
C
T
G
A
C
T
A
G
C
A
G
T
-3

-2

-1

1

2

3

4

5

6

a
a
c
g
t
g
a
g
t

Predicted cryptic donor site

T
G
A
C
C
G
T
A
C
T
A
G
C
T
A
G
A
G
C
T
C
T
G
A
C
T
G
A
C
T
A
G
C
A
G
T
3484

.

.

.

3487

.

.

.

.

.

3492

.

c
a
g
g
c
a
t
g
c
t

**11:47,367,305 G>A**

***MYBPC3***

Squirls score:
**0.049**

The variant overlaps with 4
transcripts:

| Transcript Accession | CDS Change | Variant Effect | Squirls Score |
| --- | --- | --- | --- |
| *ENST00000256993.4* | c.1090+453C>T | CODING\_TRANSCRIPT\_INTRON\_VARIANT | 0.049 |
| *ENST00000399249.2* | c.1090+453C>T | CODING\_TRANSCRIPT\_INTRON\_VARIANT | 0.049 |
| *ENST00000544791.1* | c.1090+453C>T | CODING\_TRANSCRIPT\_INTRON\_VARIANT | 0.049 |
| *ENST00000545968.1* | c.1090+453C>T | CODING\_TRANSCRIPT\_INTRON\_VARIANT | 0.049 |

Squirls features

| Feature | Value |
| --- | --- |
| *ΔRi* canonical acceptor | 0.00 |
| *ΔRi* cryptic acceptor | 5.23 |
| Creates AG in *AGEZ* | No |
| Acceptor offset | 617 |
| Exon length | -1 |
| ESRSeq | -2.57 |
| SMS | -2.59 |
| phyloP | -0.30 |

Cryptic acceptor

Using cryptic acceptor site at *11:47,367,304*
would lead to addition
of 2131
bases
to the coding sequence.

Canonical acceptor site

G
C
A
T
G
A
C
T
G
A
C
T
G
A
C
T
G
A
C
T
G
A
C
T
G
A
C
T
G
A
C
T
G
A
C
T
A
G
C
T
A
G
C
T
A
G
C
T
A
G
C
T
A
G
C
T
A
G
C
T
A
G
C
T
A
G
C
T
G
A
C
T
G
A
C
T
G
A
C
T
G
A
C
T
G
A
C
T
G
A
T
C
C
T
G
A
A
T
C
G
T
C
A
G
C
G
A
T
-25
.
.
.
.
-20
.
.
.
.
-15
.
.
.
.
.
-9
-8
-7
-6
-5
-4
-3
-2
-1
1
2
g
c
c
g
g
g
g
c
t
g
a
g
g
g
g
t
g
g
t
g
c
t
c
a
g
c
c

Predicted cryptic acceptor site

G
C
A
T
G
A
C
T
G
A
C
T
G
A
C
T
G
A
C
T
G
A
C
T
G
A
C
T
G
A
C
T
G
A
C
T
A
G
C
T
A
G
C
T
A
G
C
T
A
G
C
T
A
G
C
T
A
G
C
T
A
G
C
T
A
G
C
T
G
A
C
T
G
A
C
T
G
A
C
T
G
A
C
T
G
A
C
T
G
A
T
C
C
T
G
A
A
T
C
G
T
C
A
G
C
G
A
T
-2156
.
.
.
.
.
-2151
.
.
.
.
.
-2146
.
.
.
.
.
-2141
.
.
.
.
.
-2136
.
.
.
.
.
-2131
.
.
g
c
t
a
a
g
c
c
t
g
g
a
g
a
g
c
c
a
c
a
c
a
c
a
g
g
c
t

**X:154,130,469 T>C**

***F8***

Squirls score:
**0.049**

The variant overlaps with 1
transcript:

| Transcript Accession | CDS Change | Variant Effect | Squirls Score |
| --- | --- | --- | --- |
| *ENST00000360256.4* | c.5999-27A>G | CODING\_TRANSCRIPT\_INTRON\_VARIANT | 0.049 |

Squirls features

| Feature | Value |
| --- | --- |
| *ΔRi* canonical acceptor | 0.00 |
| *ΔRi* cryptic acceptor | -8.88 |
| Creates AG in *AGEZ* | Yes |
| Acceptor offset | -27 |
| Exon length | -1 |
| ESRSeq | -0.95 |
| SMS | -1.58 |
| phyloP | -0.24 |

Cryptic acceptor

Using cryptic acceptor site at *X:154,130,493*
would lead to addition
of 3
bases
to the coding sequence.

The variant creates *AG* dinucleotide in the
AG exclusion zone
of the canonical acceptor site.

Canonical acceptor site

G
C
A
T
G
A
C
T
G
A
C
T
G
A
C
T
G
A
C
T
G
A
C
T
G
A
C
T
G
A
C
T
G
A
C
T
A
G
C
T
A
G
C
T
A
G
C
T
A
G
C
T
A
G
C
T
A
G
C
T
A
G
C
T
A
G
C
T
G
A
C
T
G
A
C
T
G
A
C
T
G
A
C
T
G
A
C
T
G
A
T
C
C
T
G
A
A
T
C
G
T
C
A
G
C
G
A
T
-25
.
.
.
.
-20
.
.
.
.
-15
.
.
.
.
.
-9
-8
-7
-6
-5
-4
-3
-2
-1
1
2
t
t
c
t
g
t
t
c
c
t
g
t
t
g
g
t
t
t
t
t
a
t
a
a
g
g
t

Predicted cryptic acceptor site

G
C
A
T
G
A
C
T
G
A
C
T
G
A
C
T
G
A
C
T
G
A
C
T
G
A
C
T
G
A
C
T
G
A
C
T
A
G
C
T
A
G
C
T
A
G
C
T
A
G
C
T
A
G
C
T
A
G
C
T
A
G
C
T
A
G
C
T
G
A
C
T
G
A
C
T
G
A
C
T
G
A
C
T
G
A
C
T
G
A
T
C
C
T
G
A
A
T
C
G
T
C
A
G
C
G
A
T
-28
.
.
.
.
.
-23
.
.
.
.
.
-18
.
.
.
.
.
-13
.
.
.
.
.
-8
.
.
.
.
.
-3
.
.
a
a
t
t
t
c
t
g
t
t
c
c
t
g
t
t
g
g
t
t
t
t
t
a
t
a
a
g

**1:22,215,993 G>A**

***HSPG2***

Squirls score:
**0.047**

The variant overlaps with 2
transcripts:

| Transcript Accession | CDS Change | Variant Effect | Squirls Score |
| --- | --- | --- | --- |
| *ENST00000374695.3* | c.574+481C>T | CODING\_TRANSCRIPT\_INTRON\_VARIANT | 0.029 |
| *ENST00000412328.1* | c.342+481C>T | CODING\_TRANSCRIPT\_INTRON\_VARIANT | 0.029 |

Squirls features

| Feature | Value |
| --- | --- |
| *ΔRi* canonical acceptor | 0.00 |
| *ΔRi* cryptic acceptor | 0.00 |
| Creates AG in *AGEZ* | No |
| Acceptor offset | -14292 |
| Exon length | -1 |
| ESRSeq | -1.49 |
| SMS | -1.81 |
| phyloP | 0.66 |

Cryptic acceptor

**3:158,367,854 G>A**

***GFM1***

Squirls score:
**0.045**

The variant overlaps with 6
transcripts:

| Transcript Accession | CDS Change | Variant Effect | Squirls Score |
| --- | --- | --- | --- |
| *ENST00000264263.5* | c.746+4G>A | SPLICE\_REGION\_VARIANT | 0.010 |
| *ENST00000478251.1* | n.1085G>A | NON\_CODING\_TRANSCRIPT\_EXON\_VARIANT | 0.038 |
| *ENST00000478254.1* | c.689+908G>A | CODING\_TRANSCRIPT\_INTRON\_VARIANT | 0.045 |
| *ENST00000478576.1* | c.689+908G>A | CODING\_TRANSCRIPT\_INTRON\_VARIANT | 0.045 |
| *ENST00000482640.1* | c.362-3856C>T | CODING\_TRANSCRIPT\_INTRON\_VARIANT | 0.006 |
| *ENST00000486715.1* | c.689+908G>A | CODING\_TRANSCRIPT\_INTRON\_VARIANT | 0.045 |

Squirls features

| Feature | Value |
| --- | --- |
| *Ri wt* donor | 8.81 |
| *ΔRi* canonical donor | 0.00 |
| *ΔRi wt* closest donor | 0.00 |
| Donor offset | 908 |
| max *Ri* cryptic donor window | 10.33 |
| *ΔRi* cryptic donor | 1.52 |
| phyloP | 0.31 |

Cryptic donor

Using cryptic donor site at *3:158,367,851*
would lead to addition
of 905
bases
to the coding sequence.

Canonical donor site

T
G
A
C
C
G
T
A
C
T
A
G
C
T
A
G
A
G
C
T
C
T
G
A
C
T
G
A
C
T
A
G
C
A
G
T
-3

-2

-1

1

2

3

4

5

6

t
g
g
g
t
a
a
g
t

Predicted cryptic donor site

T
G
A
C
C
G
T
A
C
T
A
G
C
T
A
G
A
G
C
T
C
T
G
A
C
T
G
A
C
T
A
G
C
A
G
T
903

.

.

.

906

.

.

.

.

.

911

.

c
a
g
g
t
g
g
g
c
a

**1:94,493,901 T>C**

***ABCA4***

Squirls score:
**0.045**

The variant overlaps with 1
transcript:

| Transcript Accession | CDS Change | Variant Effect | Squirls Score |
| --- | --- | --- | --- |
| *ENST00000370225.3* | c.4539+1100A>G | CODING\_TRANSCRIPT\_INTRON\_VARIANT | 0.045 |

Squirls features

| Feature | Value |
| --- | --- |
| *Ri wt* donor | 5.98 |
| *ΔRi* canonical donor | 0.00 |
| *ΔRi wt* closest donor | 0.00 |
| Donor offset | 1100 |
| max *Ri* cryptic donor window | 7.63 |
| *ΔRi* cryptic donor | 1.65 |
| phyloP | 1.13 |

Cryptic donor

Using cryptic donor site at *1:94,493,902*
would lead to addition
of 1101
bases
to the coding sequence.

Canonical donor site

T
G
A
C
C
G
T
A
C
T
A
G
C
T
A
G
A
G
C
T
C
T
G
A
C
T
G
A
C
T
A
G
C
A
G
T
-3

-2

-1

1

2

3

4

5

6

c
a
g
g
t
a
c
c
t

Predicted cryptic donor site

T
G
A
C
C
G
T
A
C
T
A
G
C
T
A
G
A
G
C
T
C
T
G
A
C
T
G
A
C
T
A
G
C
A
G
T
1099

.

.

.

1102

.

.

.

.

.

1107

.

a
c
a
g
t
g
a
g
c
g

**17:29,487,248 C>T**

***NF1***

Squirls score:
**0.045**

The variant overlaps with 8
transcripts:

| Transcript Accession | CDS Change | Variant Effect | Squirls Score |
| --- | --- | --- | --- |
| *ENST00000356175.3* | c.288+1137C>T | CODING\_TRANSCRIPT\_INTRON\_VARIANT | 0.042 |
| *ENST00000358273.4* | c.288+1137C>T | CODING\_TRANSCRIPT\_INTRON\_VARIANT | 0.042 |
| *ENST00000431387.4* | c.288+1137C>T | CODING\_TRANSCRIPT\_INTRON\_VARIANT | 0.042 |
| *ENST00000487476.1* | n.671+1137C>T | NON\_CODING\_TRANSCRIPT\_INTRON\_VARIANT | 0.042 |
| *ENST00000489712.2* | c.87+1137C>T | CODING\_TRANSCRIPT\_INTRON\_VARIANT | 0.042 |
| *ENST00000490416.1* | n.29+1137C>T | NON\_CODING\_TRANSCRIPT\_INTRON\_VARIANT | 0.042 |
| *ENST00000495910.2* | c.170+1137C>T | CODING\_TRANSCRIPT\_INTRON\_VARIANT | 0.042 |
| *ENST00000579081.1* | c.390+1137C>T | CODING\_TRANSCRIPT\_INTRON\_VARIANT | 0.042 |

Squirls features

| Feature | Value |
| --- | --- |
| *ΔRi* canonical acceptor | 0.00 |
| *ΔRi* cryptic acceptor | 0.00 |
| Creates AG in *AGEZ* | No |
| Acceptor offset | -177587 |
| Exon length | -1 |
| ESRSeq | -2.14 |
| SMS | -1.73 |
| phyloP | 0.23 |

Cryptic acceptor

**5:112,158,419 C>T**

***APC***

Squirls score:
**0.044**

The variant overlaps with 8
transcripts:

| Transcript Accession | CDS Change | Variant Effect | Squirls Score |
| --- | --- | --- | --- |
| *ENST00000257430.4* | c.1408+731C>T | CODING\_TRANSCRIPT\_INTRON\_VARIANT | 0.013 |
| *ENST00000457016.1* | c.1408+731C>T | CODING\_TRANSCRIPT\_INTRON\_VARIANT | 0.013 |
| *ENST00000502371.1* | n.96+731C>T | NON\_CODING\_TRANSCRIPT\_INTRON\_VARIANT | 0.013 |
| *ENST00000504915.2* | c.44-585C>T | CODING\_TRANSCRIPT\_INTRON\_VARIANT | 0.043 |
| *ENST00000507379.1* | c.1354+731C>T | CODING\_TRANSCRIPT\_INTRON\_VARIANT | 0.013 |
| *ENST00000508376.2* | c.1408+731C>T | CODING\_TRANSCRIPT\_INTRON\_VARIANT | 0.013 |
| *ENST00000508624.1* | c.\*730+731C>T | THREE\_PRIME\_UTR\_INTRON\_VARIANT | 0.013 |
| *ENST00000512211.2* | c.1408+731C>T | CODING\_TRANSCRIPT\_INTRON\_VARIANT | 0.013 |

Squirls features

| Feature | Value |
| --- | --- |
| *ΔRi* canonical acceptor | 0.00 |
| *ΔRi* cryptic acceptor | 0.00 |
| Creates AG in *AGEZ* | No |
| Acceptor offset | -4491 |
| Exon length | -1 |
| ESRSeq | -3.05 |
| SMS | -2.12 |
| phyloP | 2.86 |

Cryptic acceptor

**1:216,247,476 T>C**

***USH2A***

Squirls score:
**0.044**

The variant overlaps with 2
transcripts:

| Transcript Accession | CDS Change | Variant Effect | Squirls Score |
| --- | --- | --- | --- |
| *ENST00000307340.3* | c.5573-834A>G | CODING\_TRANSCRIPT\_INTRON\_VARIANT | 0.044 |
| *ENST00000366943.2* | c.5573-834A>G | CODING\_TRANSCRIPT\_INTRON\_VARIANT | 0.044 |

Squirls features

| Feature | Value |
| --- | --- |
| *Ri wt* donor | 12.69 |
| *ΔRi* canonical donor | 0.00 |
| *ΔRi wt* closest donor | 0.00 |
| Donor offset | -1038 |
| max *Ri* cryptic donor window | 8.84 |
| *ΔRi* cryptic donor | -3.85 |
| phyloP | -0.59 |

Cryptic donor

Using cryptic donor site at *1:216,247,472*
would lead to addition
of 3951
bases
to the coding sequence.

Canonical donor site

T
G
A
C
C
G
T
A
C
T
A
G
C
T
A
G
A
G
C
T
C
T
G
A
C
T
G
A
C
T
A
G
C
A
G
T
-3

-2

-1

1

2

3

4

5

6

a
a
g
g
t
a
a
g
t

Predicted cryptic donor site

T
G
A
C
C
G
T
A
C
T
A
G
C
T
A
G
A
G
C
T
C
T
G
A
C
T
G
A
C
T
A
G
C
A
G
T
3949

.

.

.

3952

.

.

.

.

.

3957

.

g
t
g
g
t
g
a
a
t
g

**X:9,711,844 A>C**

***GPR143***

Squirls score:
**0.043**

The variant overlaps with 4
transcripts:

| Transcript Accession | CDS Change | Variant Effect | Squirls Score |
| --- | --- | --- | --- |
| *ENST00000380929.2* | c.719-131T>G | CODING\_TRANSCRIPT\_INTRON\_VARIANT | 0.043 |
| *ENST00000431126.1* | c.407-131T>G | CODING\_TRANSCRIPT\_INTRON\_VARIANT | 0.043 |
| *ENST00000447366.1* | c.465-131T>G | CODING\_TRANSCRIPT\_INTRON\_VARIANT | 0.043 |
| *ENST00000467482.1* | c.659-131T>G | CODING\_TRANSCRIPT\_INTRON\_VARIANT | 0.043 |

Squirls features

| Feature | Value |
| --- | --- |
| *Ri wt* donor | 9.72 |
| *ΔRi* canonical donor | 0.00 |
| *ΔRi wt* closest donor | 0.00 |
| Donor offset | -240 |
| max *Ri* cryptic donor window | 8.42 |
| *ΔRi* cryptic donor | -1.30 |
| phyloP | -1.45 |

Cryptic donor

Using cryptic donor site at *X:9,711,845*
would lead to addition
of 2241
bases
to the coding sequence.

Canonical donor site

T
G
A
C
C
G
T
A
C
T
A
G
C
T
A
G
A
G
C
T
C
T
G
A
C
T
G
A
C
T
A
G
C
A
G
T
-3

-2

-1

1

2

3

4

5

6

c
a
g
g
t
a
a
a
t

Predicted cryptic donor site

T
G
A
C
C
G
T
A
C
T
A
G
C
T
A
G
A
G
C
T
C
T
G
A
C
T
G
A
C
T
A
G
C
A
G
T
2239

.

.

.

2242

.

.

.

.

.

2247

.

a
a
t
g
t
c
a
g
t
g

**X:31,279,418 T>C**

***DMD***

Squirls score:
**0.043**

The variant overlaps with 13
transcripts:

| Transcript Accession | CDS Change | Variant Effect | Squirls Score |
| --- | --- | --- | --- |
| *ENST00000343523.2* | c.1845-285A>G | CODING\_TRANSCRIPT\_INTRON\_VARIANT | 0.043 |
| *ENST00000357033.4* | c.9225-285A>G | CODING\_TRANSCRIPT\_INTRON\_VARIANT | 0.043 |
| *ENST00000358062.2* | c.2313-285A>G | CODING\_TRANSCRIPT\_INTRON\_VARIANT | 0.043 |
| *ENST00000359836.1* | c.1845-285A>G | CODING\_TRANSCRIPT\_INTRON\_VARIANT | 0.043 |
| *ENST00000361471.4* | c.21-285A>G | CODING\_TRANSCRIPT\_INTRON\_VARIANT | 0.028 |
| *ENST00000378677.2* | c.9213-285A>G | CODING\_TRANSCRIPT\_INTRON\_VARIANT | 0.043 |
| *ENST00000378680.2* | c.21-285A>G | CODING\_TRANSCRIPT\_INTRON\_VARIANT | 0.028 |
| *ENST00000378702.4* | c.21-285A>G | CODING\_TRANSCRIPT\_INTRON\_VARIANT | 0.028 |
| *ENST00000378707.3* | c.1845-285A>G | CODING\_TRANSCRIPT\_INTRON\_VARIANT | 0.043 |
| *ENST00000378723.3* | c.21-285A>G | CODING\_TRANSCRIPT\_INTRON\_VARIANT | 0.028 |
| *ENST00000469142.1* | n.244-285A>G | NON\_CODING\_TRANSCRIPT\_INTRON\_VARIANT | 0.043 |
| *ENST00000474231.1* | c.1845-285A>G | CODING\_TRANSCRIPT\_INTRON\_VARIANT | 0.043 |
| *ENST00000541735.1* | c.1845-285A>G | CODING\_TRANSCRIPT\_INTRON\_VARIANT | 0.043 |

Squirls features

| Feature | Value |
| --- | --- |
| *Ri wt* donor | 9.75 |
| *ΔRi* canonical donor | 0.00 |
| *ΔRi wt* closest donor | 0.00 |
| Donor offset | -347 |
| max *Ri* cryptic donor window | 8.42 |
| *ΔRi* cryptic donor | -1.33 |
| phyloP | -0.09 |

Cryptic donor

Using cryptic donor site at *X:31,279,414*
would lead to addition
of 62293
bases
to the coding sequence.

Canonical donor site

T
G
A
C
C
G
T
A
C
T
A
G
C
T
A
G
A
G
C
T
C
T
G
A
C
T
G
A
C
T
A
G
C
A
G
T
-3

-2

-1

1

2

3

4

5

6

c
a
a
g
t
a
a
g
t

Predicted cryptic donor site

T
G
A
C
C
G
T
A
C
T
A
G
C
T
A
G
A
G
C
T
C
T
G
A
C
T
G
A
C
T
A
G
C
A
G
T
62291

.

.

.

62294

.

.

.

.

.

62299

.

a
a
g
g
t
c
a
a
t
g

**1:94,511,126 A>T**

***ABCA4***

Squirls score:
**0.042**

The variant overlaps with 1
transcript:

| Transcript Accession | CDS Change | Variant Effect | Squirls Score |
| --- | --- | --- | --- |
| *ENST00000370225.3* | c.2919-826T>A | CODING\_TRANSCRIPT\_INTRON\_VARIANT | 0.042 |

Squirls features

| Feature | Value |
| --- | --- |
| *Ri wt* donor | 6.24 |
| *ΔRi* canonical donor | 0.00 |
| *ΔRi wt* closest donor | 0.00 |
| Donor offset | -958 |
| max *Ri* cryptic donor window | 7.00 |
| *ΔRi* cryptic donor | 0.76 |
| phyloP | -2.49 |

Cryptic donor

Using cryptic donor site at *1:94,511,128*
would lead to addition
of 1351
bases
to the coding sequence.

Canonical donor site

T
G
A
C
C
G
T
A
C
T
A
G
C
T
A
G
A
G
C
T
C
T
G
A
C
T
G
A
C
T
A
G
C
A
G
T
-3

-2

-1

1

2

3

4

5

6

c
t
t
g
t
g
a
g
t

Predicted cryptic donor site

T
G
A
C
C
G
T
A
C
T
A
G
C
T
A
G
A
G
C
T
C
T
G
A
C
T
G
A
C
T
A
G
C
A
G
T
1349

.

.

.

1352

.

.

.

.

.

1357

.

a
t
g
g
t
c
a
g
a
a

**4:56,284,334 G>A**

***TMEM165***

Squirls score:
**0.042**

The variant overlaps with 9
transcripts:

| Transcript Accession | CDS Change | Variant Effect | Squirls Score |
| --- | --- | --- | --- |
| *ENST00000381334.5* | c.792+182G>A | CODING\_TRANSCRIPT\_INTRON\_VARIANT | 0.012 |
| *ENST00000506103.2* | c.\*164+3G>A | SPLICE\_REGION\_VARIANT | 0.012 |
| *ENST00000506198.1* | c.208-6371G>A | CODING\_TRANSCRIPT\_INTRON\_VARIANT | 0.042 |
| *ENST00000508404.1* | c.\*664+182G>A | THREE\_PRIME\_UTR\_INTRON\_VARIANT | 0.012 |
| *ENST00000508561.1* | n.488+3G>A | SPLICE\_REGION\_VARIANT | 0.011 |
| *ENST00000509575.1* | n.295+182G>A | NON\_CODING\_TRANSCRIPT\_INTRON\_VARIANT | 0.012 |
| *ENST00000514904.1* | n.1246+182G>A | NON\_CODING\_TRANSCRIPT\_INTRON\_VARIANT | 0.012 |
| *ENST00000542052.1* | c.603+182G>A | CODING\_TRANSCRIPT\_INTRON\_VARIANT | 0.012 |
| *ENST00000608091.1* | c.302+182G>A | CODING\_TRANSCRIPT\_INTRON\_VARIANT | 0.012 |

Squirls features

| Feature | Value |
| --- | --- |
| *Ri wt* donor | 9.38 |
| *ΔRi* canonical donor | 0.00 |
| *ΔRi wt* closest donor | 0.00 |
| Donor offset | -6477 |
| max *Ri* cryptic donor window | 8.30 |
| *ΔRi* cryptic donor | -1.08 |
| phyloP | -0.74 |

Cryptic donor

Using cryptic donor site at *4:56,284,332*
would lead to addition
of 21769
bases
to the coding sequence.

Canonical donor site

T
G
A
C
C
G
T
A
C
T
A
G
C
T
A
G
A
G
C
T
C
T
G
A
C
T
G
A
C
T
A
G
C
A
G
T
-3

-2

-1

1

2

3

4

5

6

g
a
g
g
t
g
a
g
c

Predicted cryptic donor site

T
G
A
C
C
G
T
A
C
T
A
G
C
T
A
G
A
G
C
T
C
T
G
A
C
T
G
A
C
T
A
G
C
A
G
T
21767

.

.

.

21770

.

.

.

.

.

21775

.

c
a
g
g
t
g
a
a
a
a

**5:112,112,916 A>G**

***APC***

Squirls score:
**0.039**

The variant overlaps with 6
transcripts:

| Transcript Accession | CDS Change | Variant Effect | Squirls Score |
| --- | --- | --- | --- |
| *ENST00000257430.4* | c.531+1482A>G | CODING\_TRANSCRIPT\_INTRON\_VARIANT | 0.019 |
| *ENST00000457016.1* | c.531+1482A>G | CODING\_TRANSCRIPT\_INTRON\_VARIANT | 0.019 |
| *ENST00000507379.1* | c.561+1482A>G | CODING\_TRANSCRIPT\_INTRON\_VARIANT | 0.019 |
| *ENST00000508376.2* | c.531+1482A>G | CODING\_TRANSCRIPT\_INTRON\_VARIANT | 0.019 |
| *ENST00000508624.1* | c.531+1482A>G | CODING\_TRANSCRIPT\_INTRON\_VARIANT | 0.019 |
| *ENST00000512211.2* | c.531+1482A>G | CODING\_TRANSCRIPT\_INTRON\_VARIANT | 0.019 |

Squirls features

| Feature | Value |
| --- | --- |
| *ΔRi* canonical acceptor | 0.00 |
| *ΔRi* cryptic acceptor | 0.00 |
| Creates AG in *AGEZ* | No |
| Acceptor offset | -49994 |
| Exon length | -1 |
| ESRSeq | -0.54 |
| SMS | 0.09 |
| phyloP | 1.15 |

Cryptic acceptor

**18:77,470,825 C>T**

***CTDP1***

Squirls score:
**0.038**

The variant overlaps with 3
transcripts:

| Transcript Accession | CDS Change | Variant Effect | Squirls Score |
| --- | --- | --- | --- |
| *ENST00000075430.7* | c.863+389C>T | CODING\_TRANSCRIPT\_INTRON\_VARIANT | 0.020 |
| *ENST00000299543.7* | c.863+389C>T | CODING\_TRANSCRIPT\_INTRON\_VARIANT | 0.020 |
| *ENST00000591598.1* | c.659+389C>T | CODING\_TRANSCRIPT\_INTRON\_VARIANT | 0.020 |

Squirls features

| Feature | Value |
| --- | --- |
| *ΔRi* canonical acceptor | 0.00 |
| *ΔRi* cryptic acceptor | 0.00 |
| Creates AG in *AGEZ* | No |
| Acceptor offset | -24785 |
| Exon length | -1 |
| ESRSeq | -2.03 |
| SMS | -1.57 |
| phyloP | -1.29 |

Cryptic acceptor

**7:117,229,521 A>G**

***CFTR***

Squirls score:
**0.036**

The variant overlaps with 3
transcripts:

| Transcript Accession | CDS Change | Variant Effect | Squirls Score |
| --- | --- | --- | --- |
| *ENST00000003084.6* | c.1680-886A>G | CODING\_TRANSCRIPT\_INTRON\_VARIANT | 0.036 |
| *ENST00000426809.1* | c.1590-886A>G | CODING\_TRANSCRIPT\_INTRON\_VARIANT | 0.036 |
| *ENST00000454343.1* | c.1497-886A>G | CODING\_TRANSCRIPT\_INTRON\_VARIANT | 0.036 |

Squirls features

| Feature | Value |
| --- | --- |
| *Ri wt* donor | 8.79 |
| *ΔRi* canonical donor | 0.00 |
| *ΔRi wt* closest donor | 0.00 |
| Donor offset | -973 |
| max *Ri* cryptic donor window | 8.31 |
| *ΔRi* cryptic donor | -0.48 |
| phyloP | 0.33 |

Cryptic donor

Using cryptic donor site at *7:117,229,521*
would lead to addition
of 1634
bases
to the coding sequence.

Canonical donor site

T
G
A
C
C
G
T
A
C
T
A
G
C
T
A
G
A
G
C
T
C
T
G
A
C
T
G
A
C
T
A
G
C
A
G
T
-3

-2

-1

1

2

3

4

5

6

a
a
g
g
t
g
a
a
t

Predicted cryptic donor site

T
G
A
C
C
G
T
A
C
T
A
G
C
T
A
G
A
G
C
T
C
T
G
A
C
T
G
A
C
T
A
G
C
A
G
T
1632

.

.

.

1635

.

.

.

.

.

1640

.

g
a
t
a
t
a
a
g
t
g

**16:89,577,853 A>G**

***SPG7***

Squirls score:
**0.030**

The variant overlaps with 5
transcripts:

| Transcript Accession | CDS Change | Variant Effect | Squirls Score |
| --- | --- | --- | --- |
| *ENST00000268704.2* | c.286+853A>G | CODING\_TRANSCRIPT\_INTRON\_VARIANT | 0.012 |
| *ENST00000341316.2* | c.286+853A>G | CODING\_TRANSCRIPT\_INTRON\_VARIANT | 0.012 |
| *ENST00000566371.1* | c.154+853A>G | CODING\_TRANSCRIPT\_INTRON\_VARIANT | 0.012 |
| *ENST00000568151.1* | c.382+853A>G | CODING\_TRANSCRIPT\_INTRON\_VARIANT | 0.012 |
| *ENST00000569363.1* | n.127+853A>G | NON\_CODING\_TRANSCRIPT\_INTRON\_VARIANT | 0.012 |

Squirls features

| Feature | Value |
| --- | --- |
| *ΔRi* canonical acceptor | 0.00 |
| *ΔRi* cryptic acceptor | 0.00 |
| Creates AG in *AGEZ* | No |
| Acceptor offset | -41619 |
| Exon length | -1 |
| ESRSeq | 1.36 |
| SMS | -0.56 |
| phyloP | 1.29 |

Cryptic acceptor

**12:48,380,015 A>C**

***COL2A1***

Squirls score:
**0.028**

The variant overlaps with 3
transcripts:

| Transcript Accession | CDS Change | Variant Effect | Squirls Score |
| --- | --- | --- | --- |
| *ENST00000337299.6* | c.1320+104T>G | CODING\_TRANSCRIPT\_INTRON\_VARIANT | 0.028 |
| *ENST00000380518.3* | c.1527+104T>G | CODING\_TRANSCRIPT\_INTRON\_VARIANT | 0.028 |
| *ENST00000493991.1* | n.451+104T>G | NON\_CODING\_TRANSCRIPT\_INTRON\_VARIANT | 0.028 |

Squirls features

| Feature | Value |
| --- | --- |
| *Ri wt* donor | -1.40 |
| *ΔRi* canonical donor | 0.00 |
| *ΔRi wt* closest donor | 0.00 |
| Donor offset | 104 |
| max *Ri* cryptic donor window | 3.59 |
| *ΔRi* cryptic donor | 4.99 |
| phyloP | -0.37 |

Cryptic donor

Using cryptic donor site at *12:48,380,016*
would lead to addition
of 105
bases
to the coding sequence.

Canonical donor site

T
G
A
C
C
G
T
A
C
T
A
G
C
T
A
G
A
G
C
T
C
T
G
A
C
T
G
A
C
T
A
G
C
A
G
T
-3

-2

-1

1

2

3

4

5

6

a
g
a
g
t
t
a
a
g

Predicted cryptic donor site

T
G
A
C
C
G
T
A
C
T
A
G
C
T
A
G
A
G
C
T
C
T
G
A
C
T
G
A
C
T
A
G
C
A
G
T
103

.

.

.

106

.

.

.

.

.

111

.

c
a
t
g
t
c
a
t
c
g

**3:132,436,749 G>C**

***NPHP3***

Squirls score:
**0.026**

The variant overlaps with 5
transcripts:

| Transcript Accession | CDS Change | Variant Effect | Squirls Score |
| --- | --- | --- | --- |
| *ENST00000326682.8* | c.671-996C>G | CODING\_TRANSCRIPT\_INTRON\_VARIANT | 0.007 |
| *ENST00000337331.5* | c.671-996C>G | CODING\_TRANSCRIPT\_INTRON\_VARIANT | 0.007 |
| *ENST00000465756.1* | c.377-996C>G | CODING\_TRANSCRIPT\_INTRON\_VARIANT | 0.007 |
| *ENST00000469232.1* | c.\*113+50C>G | THREE\_PRIME\_UTR\_INTRON\_VARIANT | 0.026 |
| *ENST00000471702.1* | c.671-996C>G | CODING\_TRANSCRIPT\_INTRON\_VARIANT | 0.007 |

Squirls features

| Feature | Value |
| --- | --- |
| *Ri wt* donor | 5.17 |
| *ΔRi* canonical donor | 0.00 |
| *ΔRi wt* closest donor | 0.00 |
| Donor offset | 50 |
| max *Ri* cryptic donor window | 6.35 |
| *ΔRi* cryptic donor | 1.18 |
| phyloP | -1.48 |

Cryptic donor

Using cryptic donor site at *3:132,436,746*
would lead to addition
of 47
bases
to the coding sequence.

Canonical donor site

T
G
A
C
C
G
T
A
C
T
A
G
C
T
A
G
A
G
C
T
C
T
G
A
C
T
G
A
C
T
A
G
C
A
G
T
-3

-2

-1

1

2

3

4

5

6

a
t
g
g
t
g
a
a
a

Predicted cryptic donor site

T
G
A
C
C
G
T
A
C
T
A
G
C
T
A
G
A
G
C
T
C
T
G
A
C
T
G
A
C
T
A
G
C
A
G
T
45

.

.

.

48

.

.

.

.

.

53

.

g
t
g
g
t
g
c
g
t
g

**X:20,191,268 A>C**

***RPS6KA3***

Squirls score:
**0.026**

The variant overlaps with 4
transcripts:

| Transcript Accession | CDS Change | Variant Effect | Squirls Score |
| --- | --- | --- | --- |
| *ENST00000379548.4* | c.1141-282T>G | CODING\_TRANSCRIPT\_INTRON\_VARIANT | 0.013 |
| *ENST00000379565.3* | c.1228-279T>G | CODING\_TRANSCRIPT\_INTRON\_VARIANT | 0.013 |
| *ENST00000540702.1* | c.1144-282T>G | CODING\_TRANSCRIPT\_INTRON\_VARIANT | 0.013 |
| *ENST00000544447.1* | c.1144-279T>G | CODING\_TRANSCRIPT\_INTRON\_VARIANT | 0.013 |

Squirls features

| Feature | Value |
| --- | --- |
| *ΔRi* canonical acceptor | 0.00 |
| *ΔRi* cryptic acceptor | 0.00 |
| Creates AG in *AGEZ* | No |
| Acceptor offset | 20444 |
| Exon length | -1 |
| ESRSeq | -1.21 |
| SMS | -0.76 |
| phyloP | 2.35 |

Cryptic acceptor

**22:50,502,853 A>C**

***MLC1***

Squirls score:
**0.026**

The variant overlaps with 7
transcripts:

| Transcript Accession | CDS Change | Variant Effect | Squirls Score |
| --- | --- | --- | --- |
| *ENST00000311597.5* | c.895-226T>G | CODING\_TRANSCRIPT\_INTRON\_VARIANT | 0.026 |
| *ENST00000395876.2* | c.895-226T>G | CODING\_TRANSCRIPT\_INTRON\_VARIANT | 0.026 |
| *ENST00000431262.2* | c.805-226T>G | CODING\_TRANSCRIPT\_INTRON\_VARIANT | 0.026 |
| *ENST00000450140.2* | c.739-226T>G | CODING\_TRANSCRIPT\_INTRON\_VARIANT | 0.026 |
| *ENST00000483836.1* | n.252-226T>G | NON\_CODING\_TRANSCRIPT\_INTRON\_VARIANT | 0.026 |
| *ENST00000535444.1* | c.658-226T>G | CODING\_TRANSCRIPT\_INTRON\_VARIANT | 0.026 |
| *ENST00000538737.1* | c.793-226T>G | CODING\_TRANSCRIPT\_INTRON\_VARIANT | 0.026 |

Squirls features

| Feature | Value |
| --- | --- |
| *Ri wt* donor | 8.82 |
| *ΔRi* canonical donor | 0.00 |
| *ΔRi wt* closest donor | 0.00 |
| Donor offset | -391 |
| max *Ri* cryptic donor window | 7.65 |
| *ΔRi* cryptic donor | -1.16 |
| phyloP | -0.50 |

Cryptic donor

Using cryptic donor site at *22:50,502,854*
would lead to addition
of 4010
bases
to the coding sequence.

Canonical donor site

T
G
A
C
C
G
T
A
C
T
A
G
C
T
A
G
A
G
C
T
C
T
G
A
C
T
G
A
C
T
A
G
C
A
G
T
-3

-2

-1

1

2

3

4

5

6

a
a
a
g
t
g
a
g
t

Predicted cryptic donor site

T
G
A
C
C
G
T
A
C
T
A
G
C
T
A
G
A
G
C
T
C
T
G
A
C
T
G
A
C
T
A
G
C
A
G
T
4008

.

.

.

4011

.

.

.

.

.

4016

.

c
c
t
g
t
a
t
g
t
g

**X:154,131,652 G>A**

***F8***

Squirls score:
**0.026**

The variant overlaps with 1
transcript:

| Transcript Accession | CDS Change | Variant Effect | Squirls Score |
| --- | --- | --- | --- |
| *ENST00000360256.4* | c.5998+529C>T | CODING\_TRANSCRIPT\_INTRON\_VARIANT | 0.012 |

Squirls features

| Feature | Value |
| --- | --- |
| *ΔRi* canonical acceptor | 0.00 |
| *ΔRi* cryptic acceptor | 0.00 |
| Creates AG in *AGEZ* | No |
| Acceptor offset | -17060 |
| Exon length | -1 |
| ESRSeq | -2.48 |
| SMS | -1.89 |
| phyloP | 0.31 |

Cryptic acceptor

**4:17,500,790 T>C**

***QDPR***

Squirls score:
**0.025**

The variant overlaps with 7
transcripts:

| Transcript Accession | CDS Change | Variant Effect | Squirls Score |
| --- | --- | --- | --- |
| *ENST00000281243.5* | c.436+2552A>G | CODING\_TRANSCRIPT\_INTRON\_VARIANT | 0.012 |
| *ENST00000428702.2* | c.343+2552A>G | CODING\_TRANSCRIPT\_INTRON\_VARIANT | 0.012 |
| *ENST00000505710.1* | c.363+2552A>G | CODING\_TRANSCRIPT\_INTRON\_VARIANT | 0.012 |
| *ENST00000507439.1* | c.436+2552A>G | CODING\_TRANSCRIPT\_INTRON\_VARIANT | 0.012 |
| *ENST00000508623.1* | c.436+2552A>G | CODING\_TRANSCRIPT\_INTRON\_VARIANT | 0.012 |
| *ENST00000513615.1* | c.436+2552A>G | CODING\_TRANSCRIPT\_INTRON\_VARIANT | 0.012 |
| *ENST00000514300.1* | c.\*367+2552A>G | THREE\_PRIME\_UTR\_INTRON\_VARIANT | 0.012 |

Squirls features

| Feature | Value |
| --- | --- |
| *ΔRi* canonical acceptor | 0.00 |
| *ΔRi* cryptic acceptor | 0.00 |
| Creates AG in *AGEZ* | No |
| Acceptor offset | -6654 |
| Exon length | -1 |
| ESRSeq | -2.33 |
| SMS | -1.05 |
| phyloP | -2.22 |

Cryptic acceptor

**5:178,414,570 A>AT**

***GRM6***

Squirls score:
**0.024**

The variant overlaps with 3
transcripts:

| Transcript Accession | CDS Change | Variant Effect | Squirls Score |
| --- | --- | --- | --- |
| *ENST00000231188.5* | c.1355-587\_1355-586insA | CODING\_TRANSCRIPT\_INTRON\_VARIANT | 0.013 |
| *ENST00000517717.1* | c.1355-587\_1355-586insA | CODING\_TRANSCRIPT\_INTRON\_VARIANT | 0.013 |
| *ENST00000518082.1* | n.39-587\_39-586insA | NON\_CODING\_TRANSCRIPT\_INTRON\_VARIANT | 0.013 |

Squirls features

| Feature | Value |
| --- | --- |
| *ΔRi* canonical acceptor | 0.00 |
| *ΔRi* cryptic acceptor | 0.00 |
| Creates AG in *AGEZ* | No |
| Acceptor offset | 7761 |
| Exon length | -1 |
| ESRSeq | -0.27 |
| SMS | -0.48 |
| phyloP | 1.86 |

Cryptic acceptor

**5:112,158,423 A>T**

***APC***

Squirls score:
**0.023**

The variant overlaps with 8
transcripts:

| Transcript Accession | CDS Change | Variant Effect | Squirls Score |
| --- | --- | --- | --- |
| *ENST00000257430.4* | c.1408+735A>T | CODING\_TRANSCRIPT\_INTRON\_VARIANT | 0.012 |
| *ENST00000457016.1* | c.1408+735A>T | CODING\_TRANSCRIPT\_INTRON\_VARIANT | 0.012 |
| *ENST00000502371.1* | n.96+735A>T | NON\_CODING\_TRANSCRIPT\_INTRON\_VARIANT | 0.012 |
| *ENST00000504915.2* | c.44-581A>T | CODING\_TRANSCRIPT\_INTRON\_VARIANT | 0.022 |
| *ENST00000507379.1* | c.1354+735A>T | CODING\_TRANSCRIPT\_INTRON\_VARIANT | 0.012 |
| *ENST00000508376.2* | c.1408+735A>T | CODING\_TRANSCRIPT\_INTRON\_VARIANT | 0.012 |
| *ENST00000508624.1* | c.\*730+735A>T | THREE\_PRIME\_UTR\_INTRON\_VARIANT | 0.012 |
| *ENST00000512211.2* | c.1408+735A>T | CODING\_TRANSCRIPT\_INTRON\_VARIANT | 0.012 |

Squirls features

| Feature | Value |
| --- | --- |
| *ΔRi* canonical acceptor | 0.00 |
| *ΔRi* cryptic acceptor | 0.00 |
| Creates AG in *AGEZ* | No |
| Acceptor offset | -6109 |
| Exon length | -1 |
| ESRSeq | -2.56 |
| SMS | -1.69 |
| phyloP | 3.55 |

Cryptic acceptor

**5:37,012,396 C>T**

***NIPBL***

Squirls score:
**0.023**

The variant overlaps with 2
transcripts:

| Transcript Accession | CDS Change | Variant Effect | Squirls Score |
| --- | --- | --- | --- |
| *ENST00000282516.8* | c.4560+2069C>T | CODING\_TRANSCRIPT\_INTRON\_VARIANT | 0.011 |
| *ENST00000448238.2* | c.4560+2069C>T | CODING\_TRANSCRIPT\_INTRON\_VARIANT | 0.011 |

Squirls features

| Feature | Value |
| --- | --- |
| *ΔRi* canonical acceptor | 0.00 |
| *ΔRi* cryptic acceptor | 0.00 |
| Creates AG in *AGEZ* | No |
| Acceptor offset | -46609 |
| Exon length | -1 |
| ESRSeq | -0.44 |
| SMS | -0.50 |
| phyloP | NaN |

Cryptic acceptor

**18:21,114,305 T>A**

***NPC1***

Squirls score:
**0.022**

The variant overlaps with 7
transcripts:

| Transcript Accession | CDS Change | Variant Effect | Squirls Score |
| --- | --- | --- | --- |
| *ENST00000269228.5* | c.3591+105A>T | CODING\_TRANSCRIPT\_INTRON\_VARIANT | 0.022 |
| *ENST00000412552.2* | c.2637+105A>T | CODING\_TRANSCRIPT\_INTRON\_VARIANT | 0.022 |
| *ENST00000586150.1* | c.346+105A>T | CODING\_TRANSCRIPT\_INTRON\_VARIANT | 0.022 |
| *ENST00000587163.1* | n.115+105A>T | NON\_CODING\_TRANSCRIPT\_INTRON\_VARIANT | 0.022 |
| *ENST00000588867.1* | n.451A>T | NON\_CODING\_TRANSCRIPT\_EXON\_VARIANT | 0.009 |
| *ENST00000591051.1* | c.2669+105A>T | CODING\_TRANSCRIPT\_INTRON\_VARIANT | 0.022 |
| *ENST00000591107.2* | c.268+105A>T | CODING\_TRANSCRIPT\_INTRON\_VARIANT | 0.022 |

Squirls features

| Feature | Value |
| --- | --- |
| *Ri wt* donor | 3.00 |
| *ΔRi* canonical donor | 0.00 |
| *ΔRi wt* closest donor | 0.00 |
| Donor offset | 105 |
| max *Ri* cryptic donor window | 4.30 |
| *ΔRi* cryptic donor | 1.30 |
| phyloP | -0.79 |

Cryptic donor

Using cryptic donor site at *18:21,114,304*
would lead to addition
of 104
bases
to the coding sequence.

Canonical donor site

T
G
A
C
C
G
T
A
C
T
A
G
C
T
A
G
A
G
C
T
C
T
G
A
C
T
G
A
C
T
A
G
C
A
G
T
-3

-2

-1

1

2

3

4

5

6

t
c
c
g
t
g
a
g
t

Predicted cryptic donor site

T
G
A
C
C
G
T
A
C
T
A
G
C
T
A
G
A
G
C
T
C
T
G
A
C
T
G
A
C
T
A
G
C
A
G
T
102

.

.

.

105

.

.

.

.

.

110

.

a
a
g
g
a
t
t
g
c
t

**17:29,580,296 A>G**

***NF1***

Squirls score:
**0.021**

The variant overlaps with 8
transcripts:

| Transcript Accession | CDS Change | Variant Effect | Squirls Score |
| --- | --- | --- | --- |
| *ENST00000356175.3* | c.4110+4159A>G | CODING\_TRANSCRIPT\_INTRON\_VARIANT | 0.009 |
| *ENST00000358273.4* | c.4173+278A>G | CODING\_TRANSCRIPT\_INTRON\_VARIANT | 0.009 |
| *ENST00000456735.2* | c.3108+4159A>G | CODING\_TRANSCRIPT\_INTRON\_VARIANT | 0.009 |
| *ENST00000466819.1* | c.649+278A>G | CODING\_TRANSCRIPT\_INTRON\_VARIANT | 0.010 |
| *ENST00000479614.1* | c.586+4159A>G | CODING\_TRANSCRIPT\_INTRON\_VARIANT | 0.010 |
| *ENST00000493220.1* | n.2646+4159A>G | NON\_CODING\_TRANSCRIPT\_INTRON\_VARIANT | 0.009 |
| *ENST00000495910.2* | c.\*6835A>G | THREE\_PRIME\_UTR\_EXON\_VARIANT | 0.008 |
| *ENST00000579081.1* | c.4212+4159A>G | CODING\_TRANSCRIPT\_INTRON\_VARIANT | 0.009 |

Squirls features

| Feature | Value |
| --- | --- |
| *ΔRi* canonical acceptor | 0.00 |
| *ΔRi* cryptic acceptor | 0.00 |
| Creates AG in *AGEZ* | No |
| Acceptor offset | 70771 |
| Exon length | -1 |
| ESRSeq | 0.08 |
| SMS | -1.94 |
| phyloP | 4.51 |

Cryptic acceptor

**14:21,793,624 A>G**

***RPGRIP1***

Squirls score:
**0.021**

The variant overlaps with 11
transcripts:

| Transcript Accession | CDS Change | Variant Effect | Squirls Score |
| --- | --- | --- | --- |
| *ENST00000206660.6* | c.2367+82A>G | CODING\_TRANSCRIPT\_INTRON\_VARIANT | 0.008 |
| *ENST00000307974.4* | c.444+82A>G | CODING\_TRANSCRIPT\_INTRON\_VARIANT | 0.008 |
| *ENST00000382933.4* | c.689-2158A>G | CODING\_TRANSCRIPT\_INTRON\_VARIANT | 0.021 |
| *ENST00000400017.2* | c.2367+82A>G | CODING\_TRANSCRIPT\_INTRON\_VARIANT | 0.008 |
| *ENST00000553500.1* | n.480+82A>G | NON\_CODING\_TRANSCRIPT\_INTRON\_VARIANT | 0.009 |
| *ENST00000553927.1* | n.1299+82A>G | NON\_CODING\_TRANSCRIPT\_INTRON\_VARIANT | 0.008 |
| *ENST00000555322.1* | c.\*714+82A>G | THREE\_PRIME\_UTR\_INTRON\_VARIANT | 0.008 |
| *ENST00000555489.1* | c.\*380+82A>G | THREE\_PRIME\_UTR\_INTRON\_VARIANT | 0.008 |
| *ENST00000555587.1* | c.792+82A>G | CODING\_TRANSCRIPT\_INTRON\_VARIANT | 0.008 |
| *ENST00000556336.1* | c.1682-2158A>G | CODING\_TRANSCRIPT\_INTRON\_VARIANT | 0.021 |
| *ENST00000557771.1* | c.2253+82A>G | CODING\_TRANSCRIPT\_INTRON\_VARIANT | 0.008 |

Squirls features

| Feature | Value |
| --- | --- |
| *Ri wt* donor | 4.24 |
| *ΔRi* canonical donor | 0.00 |
| *ΔRi wt* closest donor | 0.00 |
| Donor offset | -2343 |
| max *Ri* cryptic donor window | 5.61 |
| *ΔRi* cryptic donor | 1.37 |
| phyloP | -0.21 |

Cryptic donor

Using cryptic donor site at *14:21,793,624*
would lead to addition
of 3461
bases
to the coding sequence.

Canonical donor site

T
G
A
C
C
G
T
A
C
T
A
G
C
T
A
G
A
G
C
T
C
T
G
A
C
T
G
A
C
T
A
G
C
A
G
T
-3

-2

-1

1

2

3

4

5

6

c
t
g
g
c
a
a
g
t

Predicted cryptic donor site

T
G
A
C
C
G
T
A
C
T
A
G
C
T
A
G
A
G
C
T
C
T
G
A
C
T
G
A
C
T
A
G
C
A
G
T
3459

.

.

.

3462

.

.

.

.

.

3467

.

t
c
a
a
t
a
a
g
t
g

**X:153,641,699 G>A**

***TAZ***

Squirls score:
**0.021**

The variant overlaps with 21
transcripts:

| Transcript Accession | CDS Change | Variant Effect | Squirls Score |
| --- | --- | --- | --- |
| *ENST00000299328.5* | c.284+110G>A | CODING\_TRANSCRIPT\_INTRON\_VARIANT | 0.007 |
| *ENST00000350743.4* | c.284+110G>A | CODING\_TRANSCRIPT\_INTRON\_VARIANT | 0.007 |
| *ENST00000351413.4* | c.284+110G>A | CODING\_TRANSCRIPT\_INTRON\_VARIANT | 0.007 |
| *ENST00000369776.4* | c.209+110G>A | CODING\_TRANSCRIPT\_INTRON\_VARIANT | 0.007 |
| *ENST00000369790.4* | c.284+110G>A | CODING\_TRANSCRIPT\_INTRON\_VARIANT | 0.007 |
| *ENST00000369793.3* | n.698+23G>A | NON\_CODING\_TRANSCRIPT\_INTRON\_VARIANT | 0.018 |
| *ENST00000426231.1* | c.\*96+10G>A | THREE\_PRIME\_UTR\_INTRON\_VARIANT | 0.021 |
| *ENST00000426834.1* | c.338+110G>A | CODING\_TRANSCRIPT\_INTRON\_VARIANT | 0.007 |
| *ENST00000433313.1* | c.\*83+23G>A | THREE\_PRIME\_UTR\_INTRON\_VARIANT | 0.018 |
| *ENST00000439735.1* | c.284+110G>A | CODING\_TRANSCRIPT\_INTRON\_VARIANT | 0.007 |
| *ENST00000445994.1* | c.\*106G>A | THREE\_PRIME\_UTR\_EXON\_VARIANT | 0.018 |
| *ENST00000454722.1* | c.338+110G>A | CODING\_TRANSCRIPT\_INTRON\_VARIANT | 0.007 |
| *ENST00000455296.1* | c.\*102+4G>A | SPLICE\_REGION\_VARIANT | 0.011 |
| *ENST00000465540.1* | n.680G>A | NON\_CODING\_TRANSCRIPT\_EXON\_VARIANT | 0.010 |
| *ENST00000475699.1* | c.284+110G>A | CODING\_TRANSCRIPT\_INTRON\_VARIANT | 0.007 |
| *ENST00000476307.1* | n.605+110G>A | NON\_CODING\_TRANSCRIPT\_INTRON\_VARIANT | 0.007 |
| *ENST00000476679.1* | n.197+110G>A | NON\_CODING\_TRANSCRIPT\_INTRON\_VARIANT | 0.007 |
| *ENST00000476800.1* | n.272G>A | NON\_CODING\_TRANSCRIPT\_EXON\_VARIANT | 0.010 |
| *ENST00000479875.1* | n.313+110G>A | NON\_CODING\_TRANSCRIPT\_INTRON\_VARIANT | 0.007 |
| *ENST00000483674.1* | n.398G>A | NON\_CODING\_TRANSCRIPT\_EXON\_VARIANT | 0.010 |
| *ENST00000483780.1* | n.58+110G>A | NON\_CODING\_TRANSCRIPT\_INTRON\_VARIANT | 0.007 |

Squirls features

| Feature | Value |
| --- | --- |
| *Ri wt* donor | -5.58 |
| *ΔRi* canonical donor | 0.00 |
| *ΔRi wt* closest donor | 0.00 |
| Donor offset | 10 |
| max *Ri* cryptic donor window | 5.12 |
| *ΔRi* cryptic donor | 10.69 |
| phyloP | 1.50 |

Cryptic donor

Using cryptic donor site at *X:153,641,696*
would lead to addition
of 7
bases
to the coding sequence.

Canonical donor site

T
G
A
C
C
G
T
A
C
T
A
G
C
T
A
G
A
G
C
T
C
T
G
A
C
T
G
A
C
T
A
G
C
A
G
T
-3

-2

-1

1

2

3

4

5

6

g
c
a
g
t
c
c
a
g

Predicted cryptic donor site

T
G
A
C
C
G
T
A
C
T
A
G
C
T
A
G
A
G
C
T
C
T
G
A
C
T
G
A
C
T
A
G
C
A
G
T
5

.

.

.

8

.

.

.

.

.

13

.

c
a
g
g
c
a
g
g
g
a

**X:135,737,600 A>T**

***CD40LG***

Squirls score:
**0.020**

The variant overlaps with 2
transcripts:

| Transcript Accession | CDS Change | Variant Effect | Squirls Score |
| --- | --- | --- | --- |
| *ENST00000370628.2* | c.346+1011A>T | CODING\_TRANSCRIPT\_INTRON\_VARIANT | 0.015 |
| *ENST00000370629.2* | c.347-915A>T | CODING\_TRANSCRIPT\_INTRON\_VARIANT | 0.020 |

Squirls features

| Feature | Value |
| --- | --- |
| *Ri wt* donor | 10.20 |
| *ΔRi* canonical donor | 0.00 |
| *ΔRi wt* closest donor | 0.00 |
| Donor offset | -978 |
| max *Ri* cryptic donor window | 7.39 |
| *ΔRi* cryptic donor | -2.81 |
| phyloP | -1.00 |

Cryptic donor

Using cryptic donor site at *X:135,737,599*
would lead to addition
of 1010
bases
to the coding sequence.

Canonical donor site

T
G
A
C
C
G
T
A
C
T
A
G
C
T
A
G
A
G
C
T
C
T
G
A
C
T
G
A
C
T
A
G
C
A
G
T
-3

-2

-1

1

2

3

4

5

6

a
a
g
g
t
a
g
g
t

Predicted cryptic donor site

T
G
A
C
C
G
T
A
C
T
A
G
C
T
A
G
A
G
C
T
C
T
G
A
C
T
G
A
C
T
A
G
C
A
G
T
1008

.

.

.

1011

.

.

.

.

.

1016

.

t
g
g
g
a
a
a
g
a
t

**2:227,952,667 A>T**

***COL4A4***

Squirls score:
**0.020**

The variant overlaps with 2
transcripts:

| Transcript Accession | CDS Change | Variant Effect | Squirls Score |
| --- | --- | --- | --- |
| *ENST00000329662.7* | c.1623+702T>A | CODING\_TRANSCRIPT\_INTRON\_VARIANT | 0.020 |
| *ENST00000396625.3* | c.1623+702T>A | CODING\_TRANSCRIPT\_INTRON\_VARIANT | 0.020 |

Squirls features

| Feature | Value |
| --- | --- |
| *Ri wt* donor | 6.34 |
| *ΔRi* canonical donor | 0.00 |
| *ΔRi wt* closest donor | 0.00 |
| Donor offset | 702 |
| max *Ri* cryptic donor window | 7.16 |
| *ΔRi* cryptic donor | 0.82 |
| phyloP | 0.56 |

Cryptic donor

Using cryptic donor site at *2:227,952,664*
would lead to addition
of 699
bases
to the coding sequence.

Canonical donor site

T
G
A
C
C
G
T
A
C
T
A
G
C
T
A
G
A
G
C
T
C
T
G
A
C
T
G
A
C
T
A
G
C
A
G
T
-3

-2

-1

1

2

3

4

5

6

c
c
a
g
t
g
a
g
t

Predicted cryptic donor site

T
G
A
C
C
G
T
A
C
T
A
G
C
T
A
G
A
G
C
T
C
T
G
A
C
T
G
A
C
T
A
G
C
A
G
T
697

.

.

.

700

.

.

.

.

.

705

.

c
c
a
g
t
a
t
g
t
a

**3:193,362,516 A>G**

***OPA1***

Squirls score:
**0.020**

The variant overlaps with 6
transcripts:

| Transcript Accession | CDS Change | Variant Effect | Squirls Score |
| --- | --- | --- | --- |
| *ENST00000361150.2* | c.1446+622A>G | CODING\_TRANSCRIPT\_INTRON\_VARIANT | 0.010 |
| *ENST00000361510.2* | c.1608+622A>G | CODING\_TRANSCRIPT\_INTRON\_VARIANT | 0.010 |
| *ENST00000361715.2* | c.1500+622A>G | CODING\_TRANSCRIPT\_INTRON\_VARIANT | 0.010 |
| *ENST00000361828.2* | c.1497+622A>G | CODING\_TRANSCRIPT\_INTRON\_VARIANT | 0.010 |
| *ENST00000361908.3* | c.1554+622A>G | CODING\_TRANSCRIPT\_INTRON\_VARIANT | 0.010 |
| *ENST00000392438.3* | c.1443+622A>G | CODING\_TRANSCRIPT\_INTRON\_VARIANT | 0.010 |

Squirls features

| Feature | Value |
| --- | --- |
| *ΔRi* canonical acceptor | 0.00 |
| *ΔRi* cryptic acceptor | 0.00 |
| Creates AG in *AGEZ* | No |
| Acceptor offset | -4039 |
| Exon length | -1 |
| ESRSeq | 0.48 |
| SMS | -0.00 |
| phyloP | -0.14 |

Cryptic acceptor

**12:6,182,667 A>C**

***VWF***

Squirls score:
**0.019**

The variant overlaps with 2
transcripts:

| Transcript Accession | CDS Change | Variant Effect | Squirls Score |
| --- | --- | --- | --- |
| *ENST00000261405.5* | c.997+118T>G | CODING\_TRANSCRIPT\_INTRON\_VARIANT | 0.019 |
| *ENST00000538635.1* | n.420+37014T>G | NON\_CODING\_TRANSCRIPT\_INTRON\_VARIANT | 0.011 |

Squirls features

| Feature | Value |
| --- | --- |
| *Ri wt* donor | 5.99 |
| *ΔRi* canonical donor | 0.00 |
| *ΔRi wt* closest donor | 0.00 |
| Donor offset | 118 |
| max *Ri* cryptic donor window | 6.00 |
| *ΔRi* cryptic donor | 0.01 |
| phyloP | 1.76 |

Cryptic donor

Using cryptic donor site at *12:6,182,667*
would lead to addition
of 118
bases
to the coding sequence.

Canonical donor site

T
G
A
C
C
G
T
A
C
T
A
G
C
T
A
G
A
G
C
T
C
T
G
A
C
T
G
A
C
T
A
G
C
A
G
T
-3

-2

-1

1

2

3

4

5

6

c
t
g
g
t
a
a
t
g

Predicted cryptic donor site

T
G
A
C
C
G
T
A
C
T
A
G
C
T
A
G
A
G
C
T
C
T
G
A
C
T
G
A
C
T
A
G
C
A
G
T
116

.

.

.

119

.

.

.

.

.

124

.

t
t
a
t
t
a
a
g
t
g

**18:55,234,982 T>G**

***FECH***

Squirls score:
**0.018**

The variant overlaps with 6
transcripts:

| Transcript Accession | CDS Change | Variant Effect | Squirls Score |
| --- | --- | --- | --- |
| *ENST00000262093.5* | c.464-1169A>C | CODING\_TRANSCRIPT\_INTRON\_VARIANT | 0.010 |
| *ENST00000382873.3* | c.482-1169A>C | CODING\_TRANSCRIPT\_INTRON\_VARIANT | 0.010 |
| *ENST00000585494.1* | c.\*191-1169A>C | THREE\_PRIME\_UTR\_INTRON\_VARIANT | 0.010 |
| *ENST00000585699.1* | n.479-1169A>C | NON\_CODING\_TRANSCRIPT\_INTRON\_VARIANT | 0.010 |
| *ENST00000591215.1* | c.248-1169A>C | CODING\_TRANSCRIPT\_INTRON\_VARIANT | 0.010 |
| *ENST00000592699.1* | c.464-1169A>C | CODING\_TRANSCRIPT\_INTRON\_VARIANT | 0.010 |

Squirls features

| Feature | Value |
| --- | --- |
| *ΔRi* canonical acceptor | 0.00 |
| *ΔRi* cryptic acceptor | 0.00 |
| Creates AG in *AGEZ* | No |
| Acceptor offset | 5616 |
| Exon length | -1 |
| ESRSeq | 5.05 |
| SMS | 4.08 |
| phyloP | 0.14 |

Cryptic acceptor

**X:149,831,329 A>G**

***MTM1***

Squirls score:
**0.017**

The variant overlaps with 5
transcripts:

| Transcript Accession | CDS Change | Variant Effect | Squirls Score |
| --- | --- | --- | --- |
| *ENST00000306167.7* | n.1335-577A>G | NON\_CODING\_TRANSCRIPT\_INTRON\_VARIANT | 0.015 |
| *ENST00000370396.2* | c.1468-577A>G | CODING\_TRANSCRIPT\_INTRON\_VARIANT | 0.015 |
| *ENST00000413012.2* | c.1357-577A>G | CODING\_TRANSCRIPT\_INTRON\_VARIANT | 0.015 |
| *ENST00000542741.1* | c.1183-545A>G | CODING\_TRANSCRIPT\_INTRON\_VARIANT | 0.015 |
| *ENST00000543350.1* | c.1123-577A>G | CODING\_TRANSCRIPT\_INTRON\_VARIANT | 0.015 |

Squirls features

| Feature | Value |
| --- | --- |
| *ΔRi* canonical acceptor | 0.00 |
| *ΔRi* cryptic acceptor | 0.00 |
| Creates AG in *AGEZ* | No |
| Acceptor offset | 17174 |
| Exon length | -1 |
| ESRSeq | -0.66 |
| SMS | -0.68 |
| phyloP | -0.99 |

Cryptic acceptor

**6:35,477,388 C>T**

***TULP1***

Squirls score:
**0.017**

The variant overlaps with 3
transcripts:

| Transcript Accession | CDS Change | Variant Effect | Squirls Score |
| --- | --- | --- | --- |
| *ENST00000229771.6* | c.718+23G>A | CODING\_TRANSCRIPT\_INTRON\_VARIANT | 0.016 |
| *ENST00000322263.4* | c.559+23G>A | CODING\_TRANSCRIPT\_INTRON\_VARIANT | 0.016 |
| *ENST00000373892.4* | n.321-299G>A | NON\_CODING\_TRANSCRIPT\_INTRON\_VARIANT | 0.017 |

Squirls features

| Feature | Value |
| --- | --- |
| *Ri wt* donor | 7.87 |
| *ΔRi* canonical donor | 0.00 |
| *ΔRi wt* closest donor | 0.00 |
| Donor offset | -403 |
| max *Ri* cryptic donor window | 6.72 |
| *ΔRi* cryptic donor | -1.14 |
| phyloP | -0.62 |

Cryptic donor

Using cryptic donor site at *6:35,477,386*
would lead to addition
of 2035
bases
to the coding sequence.

Canonical donor site

T
G
A
C
C
G
T
A
C
T
A
G
C
T
A
G
A
G
C
T
C
T
G
A
C
T
G
A
C
T
A
G
C
A
G
T
-3

-2

-1

1

2

3

4

5

6

a
c
g
g
t
g
a
g
a

Predicted cryptic donor site

T
G
A
C
C
G
T
A
C
T
A
G
C
T
A
G
A
G
C
T
C
T
G
A
C
T
G
A
C
T
A
G
C
A
G
T
2033

.

.

.

2036

.

.

.

.

.

2041

.

c
t
g
g
t
g
t
g
g
a

**8:96,048,588 C>T**

***NDUFAF6***

Squirls score:
**0.017**

The variant overlaps with 16
transcripts:

| Transcript Accession | CDS Change | Variant Effect | Squirls Score |
| --- | --- | --- | --- |
| *ENST00000286687.4* | c.-160-11C>T | FIVE\_PRIME\_UTR\_INTRON\_VARIANT | 0.016 |
| *ENST00000396111.2* | c.144+784C>T | CODING\_TRANSCRIPT\_INTRON\_VARIANT | 0.008 |
| *ENST00000396113.1* | c.144+784C>T | CODING\_TRANSCRIPT\_INTRON\_VARIANT | 0.008 |
| *ENST00000396124.4* | c.420+784C>T | CODING\_TRANSCRIPT\_INTRON\_VARIANT | 0.008 |
| *ENST00000454358.2* | c.\*118+784C>T | THREE\_PRIME\_UTR\_INTRON\_VARIANT | 0.008 |
| *ENST00000517976.1* | c.\*177C>T | THREE\_PRIME\_UTR\_EXON\_VARIANT | 0.017 |
| *ENST00000518258.1* | c.\*173+784C>T | THREE\_PRIME\_UTR\_INTRON\_VARIANT | 0.008 |
| *ENST00000518608.1* | c.\*86+784C>T | THREE\_PRIME\_UTR\_INTRON\_VARIANT | 0.008 |
| *ENST00000519136.1* | c.87+784C>T | CODING\_TRANSCRIPT\_INTRON\_VARIANT | 0.008 |
| *ENST00000519804.1* | c.87+784C>T | CODING\_TRANSCRIPT\_INTRON\_VARIANT | 0.008 |
| *ENST00000520632.1* | c.\*119-11C>T | THREE\_PRIME\_UTR\_INTRON\_VARIANT | 0.016 |
| *ENST00000520757.1* | c.362-11C>T | CODING\_TRANSCRIPT\_INTRON\_VARIANT | 0.016 |
| *ENST00000522683.1* | c.\*232C>T | THREE\_PRIME\_UTR\_EXON\_VARIANT | 0.010 |
| *ENST00000523337.1* | c.\*173+784C>T | THREE\_PRIME\_UTR\_INTRON\_VARIANT | 0.008 |
| *ENST00000523378.1* | c.144+784C>T | CODING\_TRANSCRIPT\_INTRON\_VARIANT | 0.008 |
| *ENST00000542894.1* | c.264+784C>T | CODING\_TRANSCRIPT\_INTRON\_VARIANT | 0.008 |

Squirls features

| Feature | Value |
| --- | --- |
| *ΔRi* canonical acceptor | 0.00 |
| *ΔRi* cryptic acceptor | 3.16 |
| Creates AG in *AGEZ* | No |
| Acceptor offset | 59 |
| Exon length | 193 |
| ESRSeq | 0.40 |
| SMS | 0.28 |
| phyloP | 1.12 |

Cryptic acceptor

Using cryptic acceptor site at *8:96,048,599*
would lead to removal
of 70
bases
from the coding sequence.

Canonical acceptor site

G
C
A
T
G
A
C
T
G
A
C
T
G
A
C
T
G
A
C
T
G
A
C
T
G
A
C
T
G
A
C
T
G
A
C
T
A
G
C
T
A
G
C
T
A
G
C
T
A
G
C
T
A
G
C
T
A
G
C
T
A
G
C
T
A
G
C
T
G
A
C
T
G
A
C
T
G
A
C
T
G
A
C
T
G
A
C
T
G
A
T
C
C
T
G
A
A
T
C
G
T
C
A
G
C
G
A
T
-25
.
.
.
.
-20
.
.
.
.
-15
.
.
.
.
.
-9
-8
-7
-6
-5
-4
-3
-2
-1
1
2
g
a
t
c
t
g
t
g
t
c
c
c
t
g
t
t
g
t
a
a
t
g
c
a
g
t
g

Predicted cryptic acceptor site

G
C
A
T
G
A
C
T
G
A
C
T
G
A
C
T
G
A
C
T
G
A
C
T
G
A
C
T
G
A
C
T
G
A
C
T
A
G
C
T
A
G
C
T
A
G
C
T
A
G
C
T
A
G
C
T
A
G
C
T
A
G
C
T
A
G
C
T
G
A
C
T
G
A
C
T
G
A
C
T
G
A
C
T
G
A
C
T
G
A
T
C
C
T
G
A
A
T
C
G
T
C
A
G
C
G
A
T
46
.
.
.
.
.
51
.
.
.
.
.
56
.
.
.
.
.
61
.
.
.
.
.
66
.
.
.
.
.
71
.
.
c
t
g
a
c
a
g
t
g
g
t
a
c
a
c
c
t
c
t
t
c
t
c
a
g
g
t
t

**1:94,493,895 G>A**

***ABCA4***

Squirls score:
**0.017**

The variant overlaps with 1
transcript:

| Transcript Accession | CDS Change | Variant Effect | Squirls Score |
| --- | --- | --- | --- |
| *ENST00000370225.3* | c.4539+1106C>T | CODING\_TRANSCRIPT\_INTRON\_VARIANT | 0.017 |

Squirls features

| Feature | Value |
| --- | --- |
| *Ri wt* donor | 5.98 |
| *ΔRi* canonical donor | 0.00 |
| *ΔRi wt* closest donor | 0.00 |
| Donor offset | 1106 |
| max *Ri* cryptic donor window | 6.23 |
| *ΔRi* cryptic donor | 0.25 |
| phyloP | 0.12 |

Cryptic donor

Using cryptic donor site at *1:94,493,890*
would lead to addition
of 1101
bases
to the coding sequence.

Canonical donor site

T
G
A
C
C
G
T
A
C
T
A
G
C
T
A
G
A
G
C
T
C
T
G
A
C
T
G
A
C
T
A
G
C
A
G
T
-3

-2

-1

1

2

3

4

5

6

c
a
g
g
t
a
c
c
t

Predicted cryptic donor site

T
G
A
C
C
G
T
A
C
T
A
G
C
T
A
G
A
G
C
T
C
T
G
A
C
T
G
A
C
T
A
G
C
A
G
T
1099

.

.

.

1102

.

.

.

.

.

1107

.

a
c
a
g
t
g
a
g
c
t

**X:153,787,731 C>T**

***IKBKG***

Squirls score:
**0.016**

The variant overlaps with 14
transcripts:

| Transcript Accession | CDS Change | Variant Effect | Squirls Score |
| --- | --- | --- | --- |
| *ENST00000263518.6* | c.518+866C>T | CODING\_TRANSCRIPT\_INTRON\_VARIANT | 0.011 |
| *ENST00000369601.3* | c.518+866C>T | CODING\_TRANSCRIPT\_INTRON\_VARIANT | 0.008 |
| *ENST00000369602.3* | c.518+866C>T | CODING\_TRANSCRIPT\_INTRON\_VARIANT | 0.011 |
| *ENST00000369606.4* | c.518+866C>T | CODING\_TRANSCRIPT\_INTRON\_VARIANT | 0.008 |
| *ENST00000369607.1* | c.518+866C>T | CODING\_TRANSCRIPT\_INTRON\_VARIANT | 0.008 |
| *ENST00000369609.5* | c.722+866C>T | CODING\_TRANSCRIPT\_INTRON\_VARIANT | 0.008 |
| *ENST00000393549.2* | c.515+866C>T | CODING\_TRANSCRIPT\_INTRON\_VARIANT | 0.008 |
| *ENST00000413620.1* | c.482+866C>T | CODING\_TRANSCRIPT\_INTRON\_VARIANT | 0.008 |
| *ENST00000422680.1* | c.518+866C>T | CODING\_TRANSCRIPT\_INTRON\_VARIANT | 0.008 |
| *ENST00000424839.1* | c.116+866C>T | CODING\_TRANSCRIPT\_INTRON\_VARIANT | 0.008 |
| *ENST00000440286.1* | c.518+866C>T | CODING\_TRANSCRIPT\_INTRON\_VARIANT | 0.008 |
| *ENST00000455588.2* | c.515+866C>T | CODING\_TRANSCRIPT\_INTRON\_VARIANT | 0.008 |
| *ENST00000470142.1* | c.518+866C>T | CODING\_TRANSCRIPT\_INTRON\_VARIANT | 0.008 |
| *ENST00000490409.1* | n.200-3294C>T | NON\_CODING\_TRANSCRIPT\_INTRON\_VARIANT | 0.016 |

Squirls features

| Feature | Value |
| --- | --- |
| *ΔRi* canonical acceptor | 0.00 |
| *ΔRi* cryptic acceptor | 0.00 |
| Creates AG in *AGEZ* | No |
| Acceptor offset | 2977 |
| Exon length | -1 |
| ESRSeq | -1.42 |
| SMS | -1.31 |
| phyloP | -0.44 |

Cryptic acceptor

**12:103,248,546 A>G**

***PAH***

Squirls score:
**0.016**

The variant overlaps with 3
transcripts:

| Transcript Accession | CDS Change | Variant Effect | Squirls Score |
| --- | --- | --- | --- |
| *ENST00000307000.2* | c.691+368T>C | CODING\_TRANSCRIPT\_INTRON\_VARIANT | 0.009 |
| *ENST00000549111.1* | n.1170T>C | NON\_CODING\_TRANSCRIPT\_EXON\_VARIANT | 0.011 |
| *ENST00000553106.1* | c.706+368T>C | CODING\_TRANSCRIPT\_INTRON\_VARIANT | 0.009 |

Squirls features

| Feature | Value |
| --- | --- |
| *ΔRi* canonical acceptor | 0.00 |
| *ΔRi* cryptic acceptor | 0.00 |
| Creates AG in *AGEZ* | No |
| Acceptor offset | 22783 |
| Exon length | -1 |
| ESRSeq | 2.41 |
| SMS | 2.13 |
| phyloP | 1.92 |

Cryptic acceptor

**11:108,141,209 GGTAA>G**

***ATM***

Squirls score:
**0.016**

The variant overlaps with 4
transcripts:

| Transcript Accession | CDS Change | Variant Effect | Squirls Score |
| --- | --- | --- | --- |
| *ENST00000278616.4* | c.2839-581\_2839-578del | CODING\_TRANSCRIPT\_INTRON\_VARIANT | 0.016 |
| *ENST00000419286.1* | n.204-768\_204-765del | NON\_CODING\_TRANSCRIPT\_INTRON\_VARIANT | 0.016 |
| *ENST00000452508.2* | c.2839-581\_2839-578del | CODING\_TRANSCRIPT\_INTRON\_VARIANT | 0.016 |
| *ENST00000527805.1* | c.2839-581\_2839-578del | CODING\_TRANSCRIPT\_INTRON\_VARIANT | 0.016 |

Squirls features

| Feature | Value |
| --- | --- |
| *Ri wt* donor | 9.67 |
| *ΔRi* canonical donor | 0.00 |
| *ΔRi wt* closest donor | 0.00 |
| Donor offset | -867 |
| max *Ri* cryptic donor window | 7.28 |
| *ΔRi* cryptic donor | -2.39 |
| phyloP | 0.05 |

Cryptic donor

Using cryptic donor site at *11:108,141,210*
would lead to addition
of 1874
bases
to the coding sequence.

Canonical donor site

T
G
A
C
C
G
T
A
C
T
A
G
C
T
A
G
A
G
C
T
C
T
G
A
C
T
G
A
C
T
A
G
C
A
G
T
-3

-2

-1

1

2

3

4

5

6

a
t
g
g
t
g
a
g
t

Predicted cryptic donor site

T
G
A
C
C
G
T
A
C
T
A
G
C
T
A
G
A
G
C
T
C
T
G
A
C
T
G
A
C
T
A
G
C
A
G
T
1872

.

.

.

1875

.

.

.

.

.

1880

.

c
a
g
g
t
a
a
g
t
g
t
a

**X:33,032,666 A>T**

***DMD***

Squirls score:
**0.016**

The variant overlaps with 7
transcripts:

| Transcript Accession | CDS Change | Variant Effect | Squirls Score |
| --- | --- | --- | --- |
| *ENST00000288447.4* | c.69+5590T>A | CODING\_TRANSCRIPT\_INTRON\_VARIANT | 0.016 |
| *ENST00000357033.4* | c.93+5590T>A | CODING\_TRANSCRIPT\_INTRON\_VARIANT | 0.016 |
| *ENST00000378677.2* | c.81+5590T>A | CODING\_TRANSCRIPT\_INTRON\_VARIANT | 0.016 |
| *ENST00000420596.1* | c.93+5590T>A | CODING\_TRANSCRIPT\_INTRON\_VARIANT | 0.009 |
| *ENST00000448370.1* | c.93+5590T>A | CODING\_TRANSCRIPT\_INTRON\_VARIANT | 0.010 |
| *ENST00000472681.1* | n.138+5590T>A | NON\_CODING\_TRANSCRIPT\_INTRON\_VARIANT | 0.009 |
| *ENST00000488902.1* | n.335+5590T>A | NON\_CODING\_TRANSCRIPT\_INTRON\_VARIANT | 0.009 |

Squirls features

| Feature | Value |
| --- | --- |
| *ΔRi* canonical acceptor | 0.00 |
| *ΔRi* cryptic acceptor | 1.08 |
| Creates AG in *AGEZ* | No |
| Acceptor offset | 5652 |
| Exon length | -1 |
| ESRSeq | -1.90 |
| SMS | -2.56 |
| phyloP | 1.73 |

Cryptic acceptor

Using cryptic acceptor site at *X:33,032,668*
would lead to addition
of 164727
bases
to the coding sequence.

Canonical acceptor site

G
C
A
T
G
A
C
T
G
A
C
T
G
A
C
T
G
A
C
T
G
A
C
T
G
A
C
T
G
A
C
T
G
A
C
T
A
G
C
T
A
G
C
T
A
G
C
T
A
G
C
T
A
G
C
T
A
G
C
T
A
G
C
T
A
G
C
T
G
A
C
T
G
A
C
T
G
A
C
T
G
A
C
T
G
A
C
T
G
A
T
C
C
T
G
A
A
T
C
G
T
C
A
G
C
G
A
T
-25
.
.
.
.
-20
.
.
.
.
-15
.
.
.
.
.
-9
-8
-7
-6
-5
-4
-3
-2
-1
1
2
t
t
g
a
g
t
g
t
a
t
t
t
t
t
t
t
t
a
a
t
t
t
c
a
g
t
t

Predicted cryptic acceptor site

G
C
A
T
G
A
C
T
G
A
C
T
G
A
C
T
G
A
C
T
G
A
C
T
G
A
C
T
G
A
C
T
G
A
C
T
A
G
C
T
A
G
C
T
A
G
C
T
A
G
C
T
A
G
C
T
A
G
C
T
A
G
C
T
A
G
C
T
G
A
C
T
G
A
C
T
G
A
C
T
G
A
C
T
G
A
C
T
G
A
T
C
C
T
G
A
A
T
C
G
T
C
A
G
C
G
A
T
-164752
.
.
.
.
.
-164747
.
.
.
.
.
-164742
.
.
.
.
.
-164737
.
.
.
.
.
-164732
.
.
.
.
.
-164727
.
.
c
a
t
a
t
t
t
c
c
t
t
g
t
t
t
c
t
c
t
a
c
a
t
t
g
g
t
a

**1:216,039,721 G>A**

***USH2A***

Squirls score:
**0.016**

The variant overlaps with 2
transcripts:

| Transcript Accession | CDS Change | Variant Effect | Squirls Score |
| --- | --- | --- | --- |
| *ENST00000307340.3* | c.8845+628C>T | CODING\_TRANSCRIPT\_INTRON\_VARIANT | 0.012 |
| *ENST00000366943.2* | c.8845+628C>T | CODING\_TRANSCRIPT\_INTRON\_VARIANT | 0.012 |

Squirls features

| Feature | Value |
| --- | --- |
| *ΔRi* canonical acceptor | 0.00 |
| *ΔRi* cryptic acceptor | 0.00 |
| Creates AG in *AGEZ* | No |
| Acceptor offset | 217208 |
| Exon length | -1 |
| ESRSeq | -2.05 |
| SMS | -1.45 |
| phyloP | -1.28 |

Cryptic acceptor

**X:32,470,726 C>G**

***DMD***

Squirls score:
**0.016**

The variant overlaps with 5
transcripts:

| Transcript Accession | CDS Change | Variant Effect | Squirls Score |
| --- | --- | --- | --- |
| *ENST00000357033.4* | c.3603+2053G>C | CODING\_TRANSCRIPT\_INTRON\_VARIANT | 0.010 |
| *ENST00000378677.2* | c.3591+2053G>C | CODING\_TRANSCRIPT\_INTRON\_VARIANT | 0.010 |
| *ENST00000420596.1* | c.94-87410G>C | CODING\_TRANSCRIPT\_INTRON\_VARIANT | 0.008 |
| *ENST00000448370.1* | c.94-87899G>C | CODING\_TRANSCRIPT\_INTRON\_VARIANT | 0.008 |
| *ENST00000488902.1* | n.336-235546G>C | NON\_CODING\_TRANSCRIPT\_INTRON\_VARIANT | 0.008 |

Squirls features

| Feature | Value |
| --- | --- |
| *ΔRi* canonical acceptor | 0.00 |
| *ΔRi* cryptic acceptor | 0.00 |
| Creates AG in *AGEZ* | No |
| Acceptor offset | 121029 |
| Exon length | -1 |
| ESRSeq | 3.97 |
| SMS | 2.65 |
| phyloP | -0.55 |

Cryptic acceptor

Developed by *Daniel Danis* and *Peter N Robinson* at The Jackson Laboratory for Genomic Medicine.

Problems, suggestions, or comments? Feel free to submit an issue to our GitHub tracker.

© 2021 Daniel Danis, Peter N Robinson
