# Supplementary material for: Analyzing the performance of deep learning splice prediction algorithms
Source: PLoS One. 2026 May 13;21(5):e0348885. doi: 10.1371/journal.pone.0348885 (PMC13170886; doi:10.1371/journal.pone.0348885)
Supplement: Attachment — Submitted filename: ResponseToReviewers.pdf [file pone.0348885.s006.pdf]

We thank the editor and reviewers for their thoughtful and constructive comments. We have carefully considered each point raised and have revised the manuscript accordingly. Below we provide a point-by-point response to each comment.

Editor Comments

Comment 1: Improve benchmark independence by adding more recent, well-curated and/or high-throughput functional splicing datasets and clearly reporting canonical vs non-canonical variant composition.

Response: We have incorporated three additional benchmark datasets into the study: (1) the SPiP dataset (Leman et al., 2022), comprising 99,601 variants with experimentally validated splicing outcomes across 227 genes; (2) the Barbosa deep intronic benchmark (Barbosa et al., 2023), comprising 242 manually curated pathogenic deep intronic variants; and (3) the Barbosa ClinVar benchmark (Barbosa et al., 2023), comprising 53,600 intronic variants from ClinVar. We have also added explicit reporting of the proportion of variants altering the canonical splice site dinucleotides for each dataset. For the Barbosa deep intronic benchmark, canonical splice site variants are excluded by design, as all variants are required to be located more than 10 bp from the nearest annotated splice site.

Comment 2: Revise or justify any pre-filtering steps that may introduce bias.

Response: We have revised the Methods section to more explicitly acknowledge the potential bias introduced by the Legacy Ensemble pre-filtering step in the Extended ClinVar benchmark. Specifically, we now note that this filtering strategy may systematically exclude variants detectable only by deep learning models but not by any Legacy Ensemble algorithm, potentially conservatively underestimating the specificity advantage of deep learning approaches. We further note that this limitation is partially mitigated by the inclusion of the Barbosa deep intronic benchmark, which evaluates algorithm performance on deeply intronic variants without any pre-filtering based on legacy tool predictions.

Comment 3: The explanation for OpenSpliceAI's reduced performance on the Riepe dataset should be supported by direct empirical analysis and, where possible, validated on an expanded functional assay dataset.

Response: We have replaced the speculative explanation with a direct empirical comparison of the GENCODE v24 annotation used to train SpliceAI and the RefSeq MANE Select annotation used to train OpenSpliceAI at both the ABCA4 and MYBPC3 loci. This analysis revealed two key findings. First, ABCA4 resides on a held-out chromosome under the data split applied by both models, meaning neither was trained on ABCA4 splice sites, and any performance difference at this locus reflects generalization capacity rather than training data coverage. Second, while GENCODE v24 contributed three protein-coding isoforms for MYBPC3 encompassing 36 unique introns compared to the single MANE Select transcript with 34 introns, none of the Riepe intronic and splice-region variants in the MYBPC3 dataset fell within the GENCODE-exclusive introns. These findings rule out annotation coverage as an explanation for the observed performance gap. We further note that the convergence of this finding with the results on the Barbosa deep intronic benchmark, where OpenSpliceAI also underperformed relative to SpliceAI, suggests a more general difficulty with deeply intronic variant detection rather than a gene-specific effect.

Reviewer 1

Comment 1: The authors base one of their benchmarks on the same dataset used by CI-SpliceAI. Independent comparison should try to depart from the original advantageous datasets. Two examples of manually curated splice variant lists were suggested: https://pubmed.ncbi.nlm.nih.gov/36273432/ and https://pubmed.ncbi.nlm.nih.gov/37878682/.

Response: We thank the reviewer for this suggestion. We note that the two publications referenced correspond to the SPiP dataset (Leman et al., 2022) and the Barbosa benchmark (Barbosa et al., 2023), both of which have been incorporated into the revised manuscript as described above. We have retained the CI-SpliceAI benchmark dataset as it provides a useful point of comparison with the performance reported in the original CI-SpliceAI publication, but the addition of these independent datasets substantially strengthens the benchmark independence of our study.

Comment 2: The SpliceAI web interface reference should be reformulated.

Response: We have revised the text to explicitly identify this as the Broad Institute SpliceAI Lookup web interface and have included the URL directly in the manuscript text.

Comment 3: The pre-filtering step potentially masks true predictions unseen by legacy predictors and introduces bias toward the legacy ensemble.

Response: We have revised the Methods section to explicitly acknowledge this limitation, as described in our response to Editor Comment 2 above.

Comment 4: The proportion of canonical splice site variants should be reported for all datasets.

Response: We have added explicit reporting of canonical splice site dinucleotide-altering variant proportions for all datasets, as described in our response to Editor Comment 1 above.

Reviewer 2

Comment 1: The explanation for OpenSpliceAI's reduced performance on the Riepe dataset is speculative and inadequate without empirical backing.

Response: We have addressed this with direct empirical analysis as described in our response to Editor Comment 3 above.

Comment 2: The functional assay benchmarking should be expanded to include larger high-throughput datasets such as MFASS, MaPSy, and Vex-seq.

Response: We thank the reviewer for these suggestions. We have considered each of these resources carefully. However, these datasets present some limitations for our specific benchmarking goals. MaPSy (Soemedi et al., 2017) and MFASS (Chong et al., 2019) focus primarily on exonic variants, and MFASS and Vex-seq (Adamson et al., 2018) are based on minigene reporter systems that lack the native genomic context that SpliceAI and its reimplementations rely on for prediction, potentially limiting the interpretability of performance comparisons. Most importantly, all three datasets focus on exonic and near-exonic variants rather than the deeply intronic variants that represent the primary performance differentiator among the algorithms evaluated here. We have acknowledged these resources and their limitations in the Study Limitations section of the revised manuscript. The addition of the SPiP and Barbosa datasets, which include experimentally validated splicing outcomes across a broad range of variant types and genomic contexts, substantially expands the functional benchmarking scope of the study beyond the original Riepe dataset.

We believe these revisions comprehensively address all comments raised by the editor and reviewers and have substantially strengthened the manuscript. We thank the reviewers again for their constructive engagement with our work.
